# Genome-wide association studies in rice germplasm reveal significant genomic regions for root and yield-related traits under aerobic and irrigated conditions

**DOI:** 10.3389/fpls.2023.1143853

**Published:** 2023-07-18

**Authors:** Revadi Padmashree, Kalyani M. Barbadikar, Honnappa, Nakul D. Magar, Divya Balakrishnan, R. Lokesha, C. Gireesh, Anantha M. Siddaiah, Maganti Sheshu Madhav, Y. M Ramesha, Muralidhara Bharamappanavara, Amol S. Phule, P. Senguttuvel, J. R. Diwan, D. Subrahmanyam, Raman Menakshi Sundaram

**Affiliations:** ^1^Indian Council of Agricultural Research (ICAR)-Indian Institute of Rice Research (IIRR), Hyderabad, India; ^2^University of Agricultural Sciences (UAS), Raichur, India; ^3^Chaudhary Charan Singh University, Meerut, India; ^4^Agricultural Research Station (ARS) Dhadesugur, University of Agricultural Sciences (UAS), Raichur, India

**Keywords:** aerobic rice, marker–trait association, root traits, yield-related traits, general linear model, mixed linear model, seedling vigour index

## Abstract

The development of nutrient-use efficient rice lines is a priority amidst the changing climate and depleting resources viz., water, land, and labor for achieving sustainability in rice cultivation. Along with the traditional transplanted irrigated system of cultivation, the dry direct-seeded aerobic system is gaining ground nationwide. The root-related traits play a crucial role in nutrient acquisition, adaptation and need to be concentrated along with the yield-attributing traits. We phenotyped an association panel of 118 rice lines for seedling vigour index (SVI) traits at 14 and 21 days after sowing (DAS), root-related traits at panicle initiation (PI) stage in polythene bags under controlled aerobic condition, yield and yield-related traits under the irrigated condition at ICAR-IIRR, Hyderabad, Telangana; irrigated and aerobic conditions at ARS, Dhadesugur, Raichur, Karnataka. The panel was genotyped using simple sequence repeats (SSR) markers and genome-wide association studies were conducted for identifying marker–trait associations (MTAs). Significant correlations were recorded for root length, root dry weight with SVI, root volume at the PI stage, number of productive tillers per plant, spikelet fertility, the total number of grains per panicle with grain yield per plant under irrigated conditions, and the total number of grains per panicle with grain yield per plant under aerobic condition. The panel was divided into three sub-groups (K = 3) and correlated with the principal component analysis. The maximum number of MTAs were found on chromosomes 2, 3, and 12 with considerable phenotypic variability. Consistent MTAs were recorded for SVI traits at 14 and 21 DAS (RM25310, RM80, RM22961, RM1385), yield traits under irrigated conditions (RM2584, RM5179, RM410, RM20698, RM14753) across years at ICAR-IIRR, grain yield per plant (RM22961, RM1146) under the aerobic condition, grain yield per plant at irrigated ICAR-IIRR and SVI (RM5501), root traits at PI stage (RM2584, RM80, RM410, RM1146, RM18472). Functionally relevant genes near the MTAs through *in-silico* expression analysis in root and panicle tissues viz., *HBF2 bZIP* transcription factor, WD40 repeat-like domain, *OsPILS6a* auxin efflux carrier, *WRKY108, OsSCP42, OsMADS80*, nodulin-like domain-containing protein, amino acid transporter using various rice expression databases were identified. The identified MTAs and rice lines having high SVI traits (Langphou, TI-128, Mouli, TI-124, JBB-631-1), high yield under aerobic (Phouren, NPK-43, JBB-684, Ratnamudi, TI-112), irrigated conditions (KR-209, KR-262, Phouren, Keibi-Phou, TI-17), robust root traits like root length (MoirangPhou-Angouba, Wangoo-Phou, JBB-661, Dissi, NPK-45), root volume (Ratnachudi, KJ-221, Mow, Heimang-Phou, PUP-229) can be further employed in breeding programs for the targeted environments aimed at improving seedling vigour, yield-related traits under irrigated condition, aerobic condition as adaptability to water-saving technology.

## 1 Introduction

In the present scenario of changing climatic conditions, water scarcity poses a major challenge to Indian agriculture, especially for rice (*Oryza sativa* L.), a major staple food crop. The dry-direct seeded aerobic system of rice cultivation has been identified as an alternative system to the traditional transplanted irrigated system of rice cultivation (Chauhan and Abugho, 2013; Mahender et al., 2015). The seeds are directly sown in the non-puddled soil and moisture is maintained throughout the cultivation period without excess standing water. It is comparatively economic with a saving of approximately 73% in land preparation and 56% during crop growth (Kahani and Hittalmani, 2015; Mahender et al., 2015; Anandan et al., 2016). The establishment of a crop is critically dependent on seedling development and has been shown to correlate with yield. The seedling vigour, expressed in terms of the seedling vigour index (SVI) is majorly required for rapid and uniform emergence, especially under the declining moisture content of the upper soil layer. The root-related traits like deep rooting, branching, root volume, number of roots, root angle, root hair density, and the thickness of the xylem, contribute to the establishment and adaptation under a particular soil–water environment. The root system architecture (RSA) is a complex trait contributed by the root volume, root length, root mass, number of roots, angle of roots, number of lateral roots and root hair density etc. (Sandhu et al., 2019a; Sandhu et al., 2016). Rightly known as the hidden half, the root system architecture plays a major role in such adaptation by rapidly responding the external cues. The RSA has implications in plant development, anchorage, nutrient uptake and stress response to specific conditions based on its plasticity. The root system having robust roots and potential branching ability is crucial under the aerobic condition in rice, enabling the plants to extract water from deep soil layers (Khodaeiaminjan et al., 2023). Nevertheless, different soil conditions greatly affect the varietal ability to develop a deep root system (Sagare et al., 2020). The root dry weight, root length density, and percentage lateral roots have been found to relate with yield stability and thus its role in root adaptation to different systems of cultivation needs to be emphasized. Yield and yield-related traits are complex and depend on genetic and environmental factors (Xing and Zhang, 2010; Singh et al., 2017). The yield-related traits viz., panicle number, number of grains per panicle, grain weight, and harvest index are very crucial for selection as well as for understanding its association as per the genotype-environment interaction. There exists a correlation between nutrient uptake and root traits, yield, and yield-related traits in rice. Reports have mentioned that there exists a relationship and positive correlation between root growth parameters viz., root length, root number, root volume, root dry weight and shoot dry weight and yield of aerobic rice (Sandhu et al., 2013; Sunil and Shankaralingappa, 2014; Meena et al., 2019). Thus, the selection of rice lines having robust root systems, early seedling vigor and high yield potential under irrigated and direct seeded aerobic conditions is essential towards the climate-resilience breeding programmes.

Association mapping (AM) also known as linkage disequilibrium (LD) mapping is a rapid approach for the identification of quantitative trait loci (QTL), genes/alleles associated with agronomically economic traits. The LD is defined as non-random association of alleles at two or more different loci. This mapping is based on historic recombination events and LD by the strength of the correlation between a trait and a marker in natural populations or panels with diverse lines to identify marker–trait associations (MTAs) (Flint-Garcia et al., 2005; Zhu et al., 2008; Korte and Farlow, 2013). It has been widely deployed in various rice accessions, and core collections for the identification of MTAs for traits like yield, nutrient use efficiency, biotic/abiotic stress biofortification, nutritional parameters, etc. Genomic regions and QTLs have been identified in rice for early seedling vigor (Dixit et al., 2015; Zhang et al., 2017; Chen et al., 2019; Sandhu et al., 2019b), root traits (Sandhu et al., 2013; Sandhu et al., 2019a; Vinarao et al., 2021), yield under aerobic condition (Sandhu et al., 2015), yield under drought (Bernier et al., 2007; Venuprasad et al., 2012; Mishra et al., 2013) etc. Nevertheless, a consolidated study focusing on the seedling vigor, root system, and yield under aerobic and irrigated conditions for identification of common, consistent MTAs across years, crop growing seasons and locations along with lines suitable or adaptable for such traits needs to be investigated. In the present study, genome-wide association studies (GWAS) in a rice association panel consisting of 118 rice lines using the polymorphic simple sequence repeats (SSR) markers distributed across all the chromosomes were executed. We identified the significant marker-trait associations (MTAs) for root-related traits at seedling and panicle initiation stages under aerobic controlled conditions and yield-related traits at reproductive maturity under aerobic and irrigated field conditions at two locations and two seasons viz., wet season (*Kharif*) and dry season (*Rabi*).

## 2 Materials and methods

### 2.1 Plant material and experimental sites

**Figure 1:**
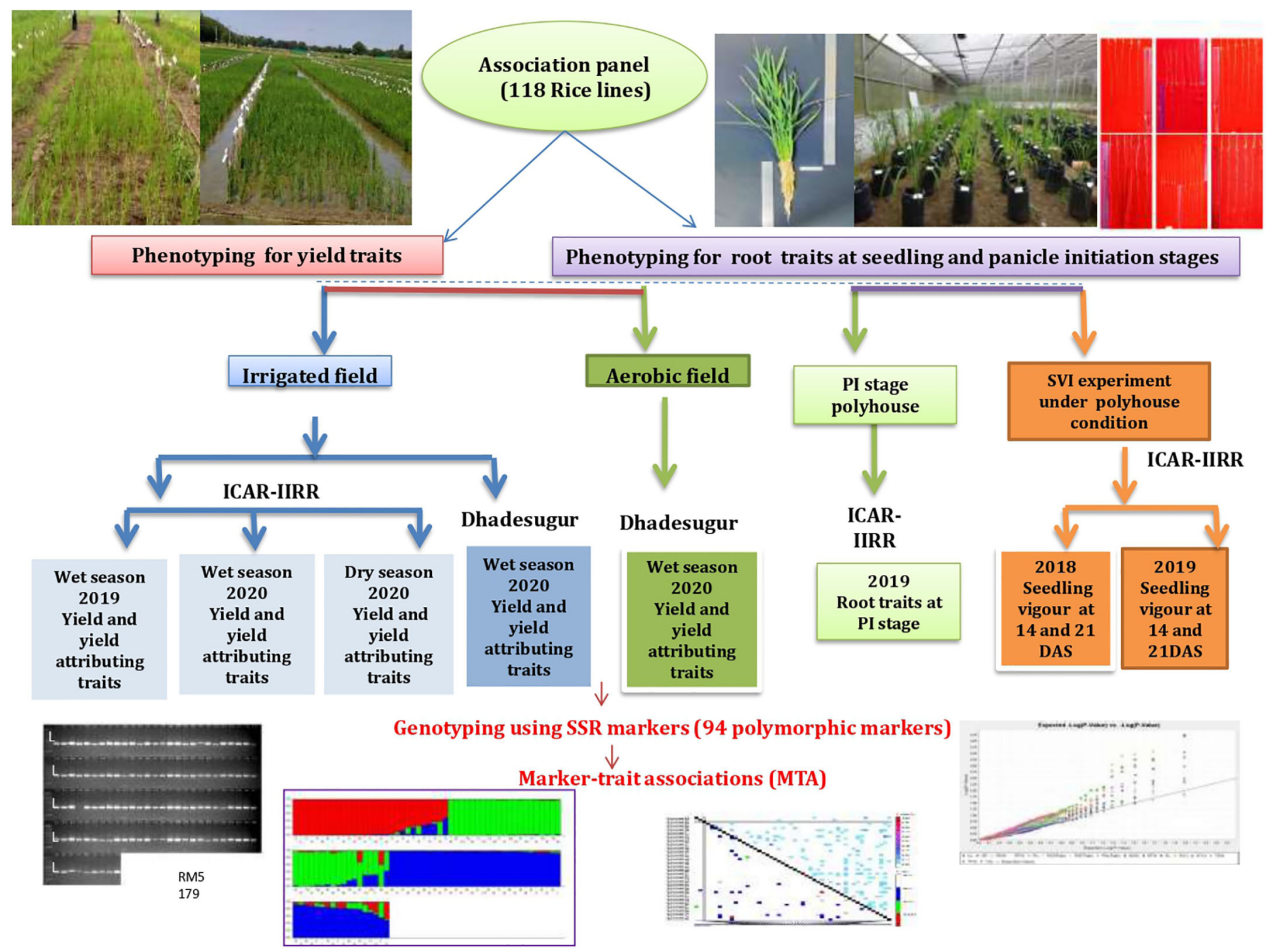
Experimental flow chart. The phenotypic traits recorded over seasons, locations, conditions, genotyping, and genome-wide association studies have been represented.

The experimental plant material for the study comprised 118 rice lines consisting of North-Eastern landraces, popularly cultivated varieties, aerobic released varieties, basmati rice, aromatic short grain lines, advanced breeding lines, introgression lines, soft rice lines, ethyl methanesulfonate (EMS) mutants of BPT-5204, and Nagina 22 (N22), wild introgression lines, *tropical japonica* accessions, *Oryza glaberrima* accessions. This panel was formed based on the genetic diversity among the lines and, prior information on yield-related traits and will be hereafter referred to as an association panel (AP). These lines were collected from the breeders of ICAR-Indian Institute of Rice Research (IIRR), Hyderabad (**Supplementary Table S1**). The experimental design and strategy along with locations have been illustrated in **Figure 1**.

### 2.2 Phenotyping

**Experiment 1: Evaluation of rice association panel for root-related traits in polyhouse under aerobic condition**

**a) At the seedling stage for seedling vigour traits (SVI_P)**

Phenotyping of seedling vigour traits at 14 and 21 days after sowing (DAS) was carried out in polyhouse under aerobic condition at ICAR-IIRR Hyderabad for two years 2018 and 2019 (**Supplementary Figure S1A**). The lines of the association panel were directly sown in black polythene covers of 80 cm length containing 15 Kg soil in two replications. To maintain the aerobic condition, need-based irrigation was given (water in a measured volume of 150 ml). The soil macro, micronutrients, pH were maintained throughout the experiment, and recommended dose of fertilizer was provided. The seedlings were removed carefully from each polythene cover in replications at two seedling growth stages (14^th^, 21^st^ DAS), to record seedling vigour traits viz., germination per cent (G %), shoot length (SL), root length (RL), total seedling length (TSL). The SL, RL, TSL were recorded manually using a centimeter scale. The shoot fresh weight (SFW), root fresh weight (RFW), total fresh weight (TFW), shoot dry weight (SDW), root dry weight (RDW), and total dry weight (TDW) were recorded using an electronic balance (iGene, India). The root to shoot length ratio (RSLR), root to shoot fresh weight and dry weight ratios (RSFWR and RSDWR) were derivative parameters calculated by division. The root surface area (RSA), root average diameter (RAD), root length per volume (RLPV) and root volume (RV) were recorded using WinRHIZO Pro software (*version 4*) (Arsenault et al., 1995). The Seedling Vigour Index-I (SVI-I) and Seedling Vigour Index-II (SVI-II) were calculated using the below-given formulae by Addanki et al., 2019 viz., Seedling Vigour Index-I = germination percentage × seedling length(cm), Seedling Vigour Index-II = germination percentage × total dry weight (mg). After 21 days single plant per/polythene bag was maintained till the panicle initiation (PI) stage (**Supplementary Figure S1B**).

**b) At the panicle initiation (PI) stage for root architecture traits (Root_PI)**

The panicle initiation stage differed considerably in the association panel for each line and according to the PI stage/booting stage of each line, the whole plant in each polythene bag was carefully removed by cutting the polythene cover without damaging the roots (**Supplementary Figure S2**). The roots were washed carefully using a high-pressure water pump (Barbadikar et al., 2016), and the same samples were phenotyped for traits like SL, RL, total plant length (TPL), SFW, RFW, TFW, SDW, RDW, TDW, RSLR, tiller number (TN), RSFWR and RSDWR were measured manually (**Supplementary Figure S3**). The RAD, RLPV and RV were analyzed and recorded in WinRHIZO Pro software (*version 4*) (Arsenault et al., 1995) (**Supplementary Figure S4**). Chlorophyll content was recorded using the Soil Plant Analysis Development (SPAD) chlorophyll meter (SPAD-502 plus Minolta, New Jersey, USA) in the morning hours between 7 to 9 am (Yugandhar et al., 2018).

**Experiment 2**

**Field evaluation under the irrigated condition at ICAR-IIRR, Rajendranagar Hyderabad for yield and yield-related traits (Irri_RJN))**

The seeds of the association panel were grown under irrigated conditions during the wet season 2019 and 2020 and dry season 2020 at ICAR-IIRR, Rajendranagar, Hyderabad. The experiment was laid out in Augmented Randomized Complete Block Design (RCBD) wherein, each block comprised of 23 rice lines along with checks (BPT-5204, Swarna, MTU-1010, RNR-15048) under irrigated conditions. The seeds were sown on the nursery beds and 30 days after sowing, the plants were transplanted under irrigated conditions (**Supplementary Figures S5A, B**). The agronomic practices were followed as recommended for irrigated rice cultivation. The data was recorded for three plants per line for the traits, viz., plant height (PH), panicle length (PL), number of tillers per plant (NTP), number of panicles per plant (NPP), the total number of grains per panicle (TNG), per cent spikelet fertility (SF), test weight (TW), grain yield per plant (GYP), straw weight (SW). The ratio of the number of filled spikelets per panicle to the total number of spikelets per panicle was expressed in percentage as spikelet fertility.

**Experiment 3**

**Field evaluation under irrigated and aerobic conditions at Dhadesugur for yield and yield-related traits (Irri_DSG-Irrigated, Dhadesugur, Aero_DSG, Dhadesugur)**

**Irrigated, Dhadesugur**

The rice association panel was evaluated for yield and yield-related traits under irrigated and aerobic conditions in the wet season 2020 at the Agricultural Research Station (ARS), Dhadesugur, University of Agricultural Sciences, Raichur. The irrigated field design and maintenance were similar to experiment 2 with a recommended package of practices.

**Aerobic, Dhadesugur**

For the aerobic condition, the experiment was laid out in Augmented Randomized Complete Block Design with five blocks; each block consisting of 30 diverse rice lines along with checks (viz., Sabita, Sahbhagi Dhan, MAS 946-1, DRR Dhan-41, DRR Dhan-42, DRR Dhan-44, CR Dhan-201, and CR Dhan-202). The seeds were dry directly sown (each line was sown in two rows of two-meter length at a spacing of 20 cm x 15 cm) and 15 days after sowing extra seedlings were thinned manually and a single plant per hill was maintained (**Supplementary Figures S6A, B**). Surface irrigation was provided need-based as per the soil and weather conditions. Agronomic practices were followed as recommended for aerobic rice cultivation along with timely weeding (Ramyajit et al., 2019). The traits were recorded as mentioned in experiment 2. Straw weight in three biological replications was measured on a balance and harvest index (HI) (ratio of economic yield to the biological yield) was calculated in percentage (Balakrishnan et al., 2016; Veronica et al., 2019).

### 2.3 Statistical analysis

The augmented analysis of variance (ANOVA) for yield-related traits and completely randomized design (CRD) ANOVA for root-related traits, estimation of the genotypic coefficient of variability, phenotypic coefficient of variability, broad-sense heritability and genetic advance as per cent mean were executed in R Studio (*version 4.0.2*) (https://cloud.r-project.org/package=augmented RCBD) using the *R* script. This package also provided histograms and box plots for each trait. Correlation analysis and correlation plots were plotted using past3 *V 3.22* (https://past.en.lo4d.com/windows).

### 2.4 Genotyping

Genomic DNA was isolated from the leaves at 21 days old seedlings using the CTAB method of the association panel (Murray and Thompson, 1980). The quality of DNA was assessed on agarose gel (0.8%) and quantified using a Nanodrop spectrophotometer (ND1000) (Thermo Scientific, USA). The association panel was genotyped using 154 SSR markers (84-hyper variable markers, 30-trait linked, 40-QTL linked markers) covering the 12 chromosomes of rice. Related information of 94 polymorphic SSR markers has been provided in **Supplementary Table S2**. The alleles were scored as ABCD, the lowermost or first allele is considered as A, the second as B, third allele as C and the uppermost as D, according to amplicon sizes and also in 1010 format for genetic diversity estimates and marker–trait associations.

The genetic diversity among the lines of the association panel was calculated using DARwin 5.0 (https://darwin.cirad.frunweightedeighted) Neighbor-joining (NJ) tree was constructed based on a dissimilarity matrix for a distance-based approach. The principal component analysis (PCA) bi-plot was generated based on the principal components and Eigen values in DARwin. The polymorphic information content (PIC) of each marker was calculated (Anderson and Sullivan, 1993). The hierarchical distribution of the molecular variance (AMOVA) within and between subgroups defined by Structure and Shannon’s information was executed using GenAlEx 6.503.

### 2.5 Population structure

The population structure of the association panel was determined using STRUCTURE *V.2.3.4* (Pritchard et al., 2000). A series of models with K values ranging from 1 to 10 with a burn-in period of 50,000 and a running length of 10,000 was executed for reliable results. For visualizing the results of STRUCTURE, the results were fed in the Structure Harvester (Earl and vonHoldt, 2012) to plot the mean likelihood values per K and exported as a tab-delimited table of the Evanno results. Two independent runs for each K were performed twice for the reproducibility and accuracy of the results. The K value with the maximum likelihood over the runs was considered as the most probable number of clusters i.e. sub-populations of the association panel. The pair-wise fixation index FST histograms were plotted for each of the clusters or the sub-populations according to the alpha values.

### 2.6 Genome-wide association studies for marker–trait associations

Association between the recorded phenotypic traits and SSR-derived genotypic data was carried out using TASSEL 4 (https://bitbucket.org/tasseladmAn/tassel-4source/wiki/UserManual) for seedling vigour traits at 14 and 21 DAS under polyhouse conditions, for root traits at PI stage, yield and yield-related traits under aerobic and irrigated conditions. The allelic data of the polymorphic SSR markers along with the phenotyping data were used as input in the TASSEL 4. The statistical models mixed linear model (MLM) that considers the kinship *K* and population structure Q (*K+Q*) and generalized linear model (GLM) were used for assessing the marker-trait associations. The corresponding p-values were corrected based on the marker F test; a false discovery rate (FDR) of 0.05 was selected as a threshold for significant associations, according to Benjamini and Hochberg (1995). The statistically significant marker–trait associations (MTAs) were considered at p-value of <0.05 and R^2^ value of >0.1. The corresponding markers pair-wise LD plots were plotted. The Manhattan plots were plotted for phenotypic traits based on the negative log value of p. The rooted tree diagram was plotted based on the genetic distance PCA in TASSEL 4 function. The markers having significant associations with the traits under both MLM and GLM were considered. A stringent criterion was followed for filtering the significant MTAs (R^2^>0.1 and p<0.05) by considering only the markers having significant association with more than two traits in all the data using Microsoft excel.

### 2.7 *In-silico* identification of genes

The significantly linked SSR markers associated with more than two phenotypic traits were selected for mining the nearby genes in the 1Mb spanning region using MSU v.7 rice genome browser (http://rice.plantbiology.msu.edu/cgi-bin/gbrowse/rice/#search). The genes were selected and thoroughly checked for their function, expression using the locus search and gene information available at the Rice Annotation Project Database (RAP-DB) (https://rapdb.dna.affrc.go.jp/) and Rice Genome Annotation Project (RGAP) (http://rice.plantbiology.msu.edu/cgi-bin/) data putative functions and gene annotation assignments (GO assignments). In depth *in silico* analysis of the genes associated with the marker was executed using the available expression databases viz., Rice Expression Database (RED) IC4R (http://expression.ic4r.org/), RiceXpro (https://ricexpro.dna.affrc.go.jp/), RGI Rice Gene Index (https://riceome.hzau.edu.cn), NetREx Network-based Rice Expression Analysis (https://bioinf.iiit.ac.in/netrex/index.html). The expression of the genes was checked in the specific databases based on the respective database’s specifications, tissues, experimental conditions, etc. The expression of genes was recorded specifically in the roots, and panicles at the seedling and reproductive stage according to the available information in the database. A set of specific genes expressed in the roots and panicles were further considered and an apparent score was given to each gene on the basis of the expression values in all the databases. The scores were represented as a heat map using Microsoft Excel.

## 3 Results

### 3.1 Phenotyping and trait correlation in polyhouse under aerobic condition

**At the seedling stage for seedling vigour traits (SVI_P)**

**Figure 2:**
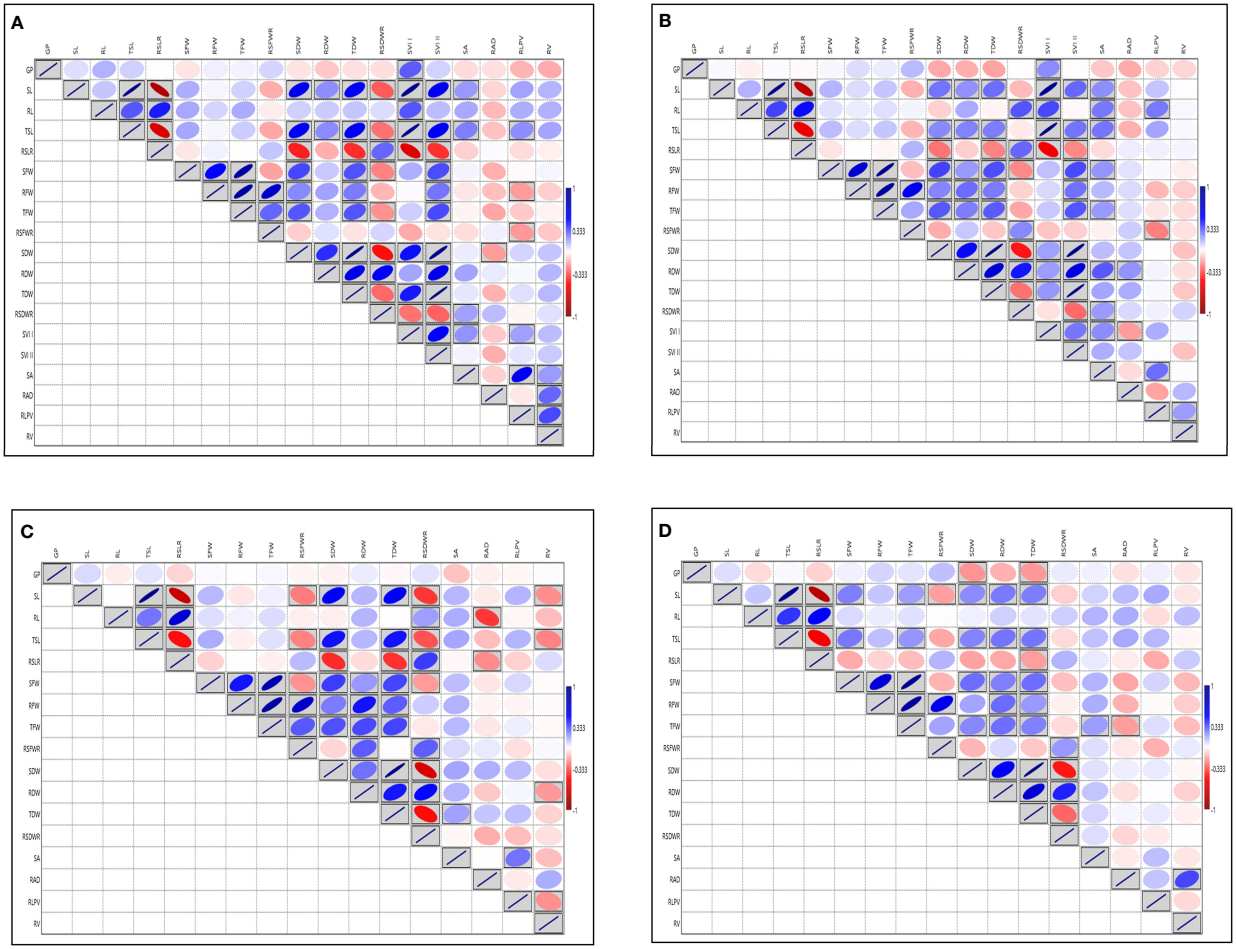
Graphical representation of correlation coefficients in rice association panel for seedling vigour index traits under polyhouse **(A)** 14 DAS 2018, **(B)** 21 DAS 2018, **(C)** 14 DAS 2019, **(D)** 21 DAS 2019. GP, Germination (%); SL, Shoot length (cm); RL, Root length (cm); TSL, Total seedling length (cm); RSLR, Root to shoot length ratio; SFW, Shoot fresh weight (mg); RFW, Root fresh weight (mg); TFW, Total fresh weight (mg); RSFWR, Root to shoot fresh weight ratio; SDW, Shoot dry weight (mg); RDW, Root dry weight (mg); TDW, Total dry weight (mg); RSDWR, Root to shoot dry weight ratio; SVI-I, Seedling vigour index-I and SVI-II, Seedling vigour index-II; RSA, Root surface area (cm^2^); RAD, Root average diameter (mm); RLPV, Root length per volume (cm/m^3^); RV, Root volume (cm^3^). Red box: Indicates negative correlation; Blue box: Indicates positive correlation; Clockwise direction of the boxes indicates the intensity of positive correlation; anticlockwise direction of the boxes indicates the intensity of negative correlation.

Analysis of variance for SVI traits viz., G (%), SL, RL, TSL, SFW, RFW, TFW, SDW, RDW, TDW, SRLR, RSFWR, RSDWR, RAD, RLPV, SVI-I, SVI-II showed significant variation except for the traits RSA, RV at 14 DAS during 2018 and G (%) at 14 DAS during 2019 respectively (**Supplementary Table S3A**). Root dry weight ranged from (1.24 to 9.57 mg) and (2.16 to 19.20 mg), (1.33 to 11.32 mg) and (2.50 to 21.92 mg) and TDW ranged from (4.48 to 48.20 mg) and (8.15 to 90 mg), (5.71 to 47.72 mg) and (10.42 to 88.08 mg) at 14 and 21 DAS during 2018 and 2019 respectively, RDW and TDW exhibited the highest GCV and PCV coupled with high heritability and high GAM, followed by RSA, RAD, RLPV, and RV. Moderate to high GCV and PCV with moderate heritability coupled with high GAM was exhibited by the trait RL at 14DAS consistently in both seasons (**Supplementary Table S3B**; **Additional File 1 Figures 1**, **2**). The RL also showed a significant positive correlation with RLPV (0.122 and 0.203) at 14 and 21 DAS during 2018, and it is positively correlated with SVI-1 in 2019 (**Figure 2**). Based on the mean performance of the early seedling vigour traits like SL, RL, RDW and RV, lines viz., Langphou, Mouli, TI-128, TI-124, JBB-631-1, Ratnamudi, Tellahamsa and KR-262 performed better as compared to the popular seedling vigour check Sabita.

**At the panicle initiation stage for root architecture traits**

**Figure 3:**
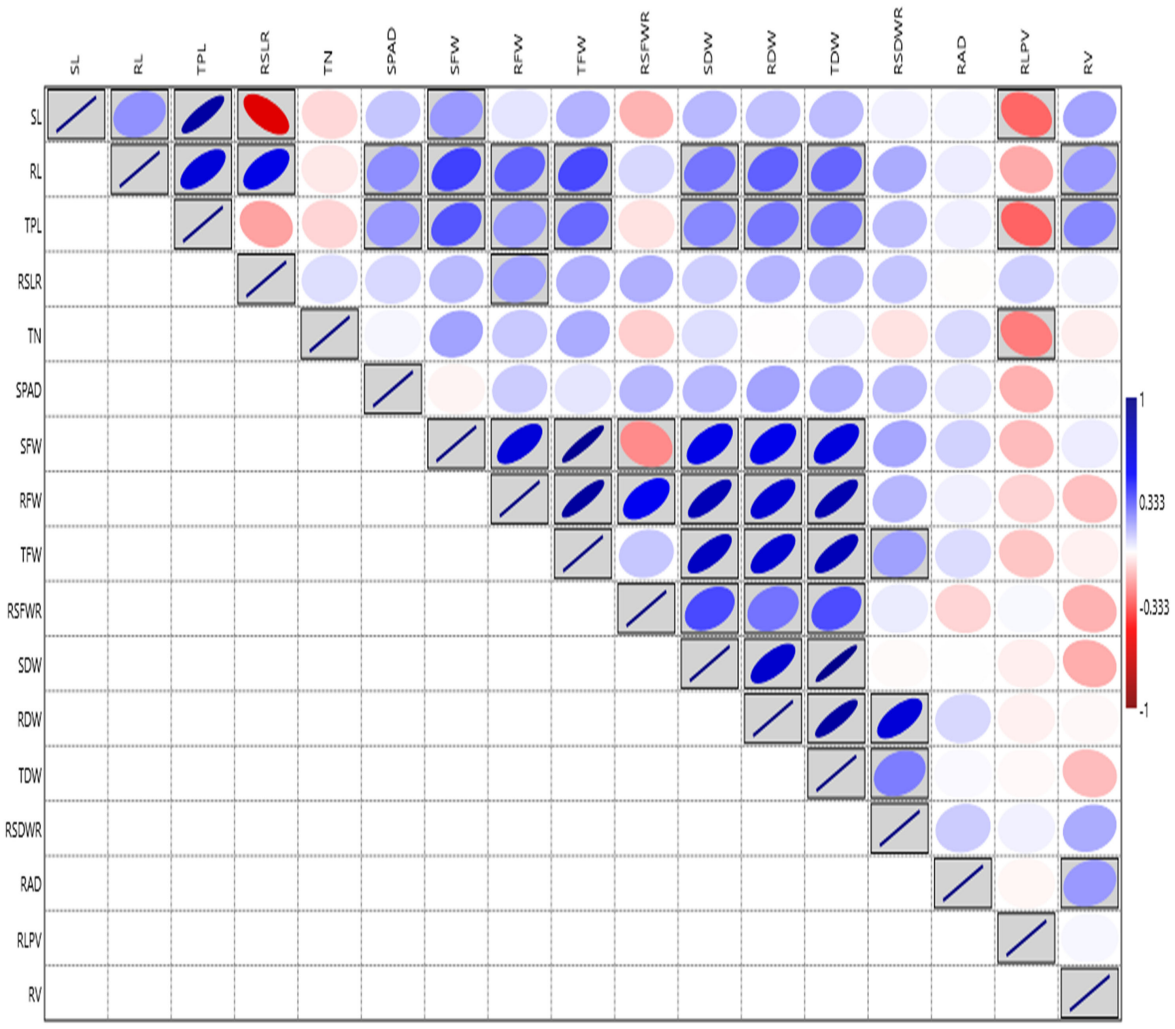
Graphical representation of correlation coefficients in rice association panel for root-related traits at panicle initiation stage under polyhouse condition. SL, Shoot length (cm); RL, Root length (cm); TPL, Total Plant length (cm); RSLR, Root to shoot length ratio; TN, Tiller number; SPAD, Soil Plant Analysis Development; SFW, Shoot fresh weight (g); RFW, Root fresh weight (g); TFW, Total fresh weight (g); RSFWR, Root to shoot fresh weight ratio; SDW, Shoot dry weight (g); RDW, Root dry weight (g); TDW, Total dry weight (g); RSDWR, Root to shoot dry weight ratio; RAD, Root average diameter (mm); RLPV, Root length per volume (cm/m^3^); RV, Root volume(cm^3^). Red box: Indicates negative correlation; Blue box: Indicates positive correlation; Clockwise direction of the boxes indicates the intensity of positive correlation; anticlockwise direction of the boxes indicates the intensity of a negative correlation.

Root architecture traits like SL, RL, TPL, RSLR, TN, SPAD, SFW, RFW, TFW, SDW, and TDW, displayed significant variation among the lines at the PI stage under aerobic condition during 2019 (**Supplementary Table S3C**). The RV ranged from (3.36 to 133.49 cm^3^) showed the highest GCV and PCV coupled with high heritability and high GAM, followed by RAD with the range 1.05 to 15.40 mm (**Supplementary Table S3D**, **Additional File 1 Figure 3**). The highest correlation was observed for SFW with TFW (0.946). The RL ranged from (21.50 to 73.60 cm) and exhibited the highest positive and significant correlation with TPL (0.659), high correlation with TDW, RFW, RDW and SPAD. The SPAD meter reading showed the highest correlation with RAD (0.135) and RDW (0.134 mg) (**Figure 3**). Based on the overall mean performances of traits viz., RL, RDW and RV the lines viz., NPK-45, MoirangPhou-Angouba, JBB-661, Wangoo-Phou, GNV-1109, NPK-13, Dissi, SM-686 and TI-166 performed better than aerobic adapted checks viz., MAS-946-1, CR Dhan-201 and CR Dhan-202 under aerobic condition.

### 3.2 Yield and yield-related traits under irrigated field conditions at ICAR-IIRR Hyderabad (Irri_RJN-Irrigated, Rajendranagar)

**Figure 4:**
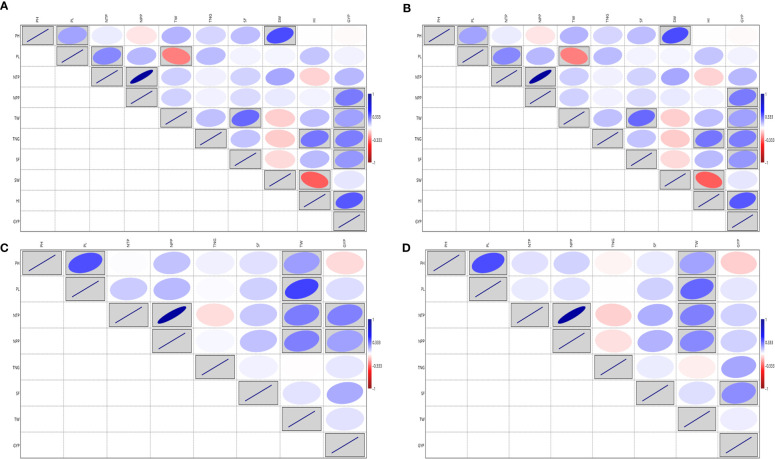
Correlation for the yield and yield-related traits in rice association panel. **(A)** ICAR-IIRR during *Wet season* 2020, **(B)** ICAR-IIRR during *Dry season* 2020, **(C)** Dhadesugur during *Wet season* 2020, **(D)** Aerobic condition Dhadesugur during *Wet season* 2020. PH, Plant height (cm); PL, Panicle length (cm); NTP, Number of tillers per plant; NPP, Number of panicle per plant; TNG, total number of grains per panicle; SF, Per cent spikelet fertility; TW, Test weight (g); SW, Straw weight (g); HI, Harvest index (%); GYP, Grain yield per plant (g). Red box: Indicates negative correlation and Blue box: Indicates positive correlation; Clockwise direction of the boxes indicates intensity of positive correlation; the anticlockwise direction of the boxes indicates intensity of negative correlation.

The analysis of variance for the traits viz., PH, PL, NTP, TNG, SF and TW showed a significant variation except for NPP in test *vs* check under irrigated conditions during the wet season 2019. Similarly, test lines exhibited significant variability for all the above-mentioned traits except for NPP (**Supplementary Table S3E**). High GCV and PCV coupled with high heritability and high GAM were exhibited by the traits GYP, PH, TW, SF and TNG (**Supplementary Table S3F** **Additional File 1 Figure 4**). The GYP exhibited a significant correlation with NTP (0.318), NPP (0.276) and SF (0.119). The NPP exhibited a significant correlation with the NTP (0.79). The analysis of variance for the traits viz., PH, PL, NTP, NPP, TNG, SF and TW under irrigated showed a significant variation except for PL in dry season 2020 and SF in both wet and dry seasons 2020 in the test vs check under irrigated condition. Similarly, test lines exhibited significant variability for all the above-mentioned traits except for NTP in both the conditions and the NPP only in the wet season 2020 (**Supplementary Table S3G**). High GCV, and PCV coupled with high heritability and high GAM were exhibited by the traits GYP, TW and TNG considering both the seasons (**Supplementary Table S3H** and **Additional File 1 Figure 5**). The NPP exhibited a significant correlation with the NTP (0.886 and 0.878) in both wet and dry season 2020 respectively, followed by GYP with TNG under both the seasons (**Figures 4A, B**). Based on the mean performance of GYP, TW, SF, and NPP the lines viz., KR-209, KR-262, Phouren, and TI-17 performed better than the checks namely BPT-5204, Swarna, MTU-1010, RNR-15048.

### 3.3 Yield and yield-related traits under irrigated and aerobic field conditions at Dhadesugur (Irri_DSG-Irrigated, Dhadesugur, Aero_DSG, Dhadesugur)

**Irrigated Dhadesugur**

The augmented ANOVA for yield and yield-related traits under the irrigated condition during wet season 2020 for the traits, PL, NTP, NPP, TNG, SF and TW exhibited high variability in test vs checks. All the traits recorded significant variation among the test lines except NTP and NPP (**Supplementary Table S3I**). The NPP exhibited the highest GCV and PCV coupled with high heritability and high GAM followed by NTP (**Supplementary Table S3J** and **Additional File 1 Figure 6**) and NPP also exhibited the highest positive and significant association with the NTP (0.882) and GYP (**Figure 4C**). Based on the mean performance of GYP, TW, SF and NPP the lines viz., Langphou, KR-209, JBB-631-1 and GNV-14-96-1 performed better than the irrigated checks BPT-5204, Swarna, MTU-1010, RNR-15048.

**Aerobic Dhadesugur**

The augmented analysis of variance for yield and yield-related traits during wet season 2020 for the traits viz., PH, PL, NTP, NPP, SF, TW, SW, HI and GYP, showed significant variability among the lines, similarly, significant variability was observed for test vs checks except for the SW under aerobic condition (**Supplementary Table S3K**). The HI exhibited the highest GCV and PCV coupled with high heritability and high GAM, followed by PL, GYP and TW (**Supplementary Table S3L** and **Additional File 1 Figure 7**). The highest significant correlation was observed for NPP with NTP (0.88). The GYP ranged from 3.32 to 21.12 g and exhibited the highest positive and significant correlation value with HI followed by the NPP, TNG and SF (**Figure 4D**). Based on the mean performance of GYP, TW, SF and NPP the lines viz., JBB-631-1, Phouren, Ratnamudi, NPK-43, TI-112, TI-87 and JBB-684 performed better under aerobic conditions than the aerobic adapted checks Sabita, Sahbhagi Dhan, MAS 946-1, DRR Dhan-41, DRR Dhan-42, DRR Dhan-44, CR Dhan-201, CR Dhan-202.

### 3.4 Genotyping and diversity analysis

**Figure 5:**
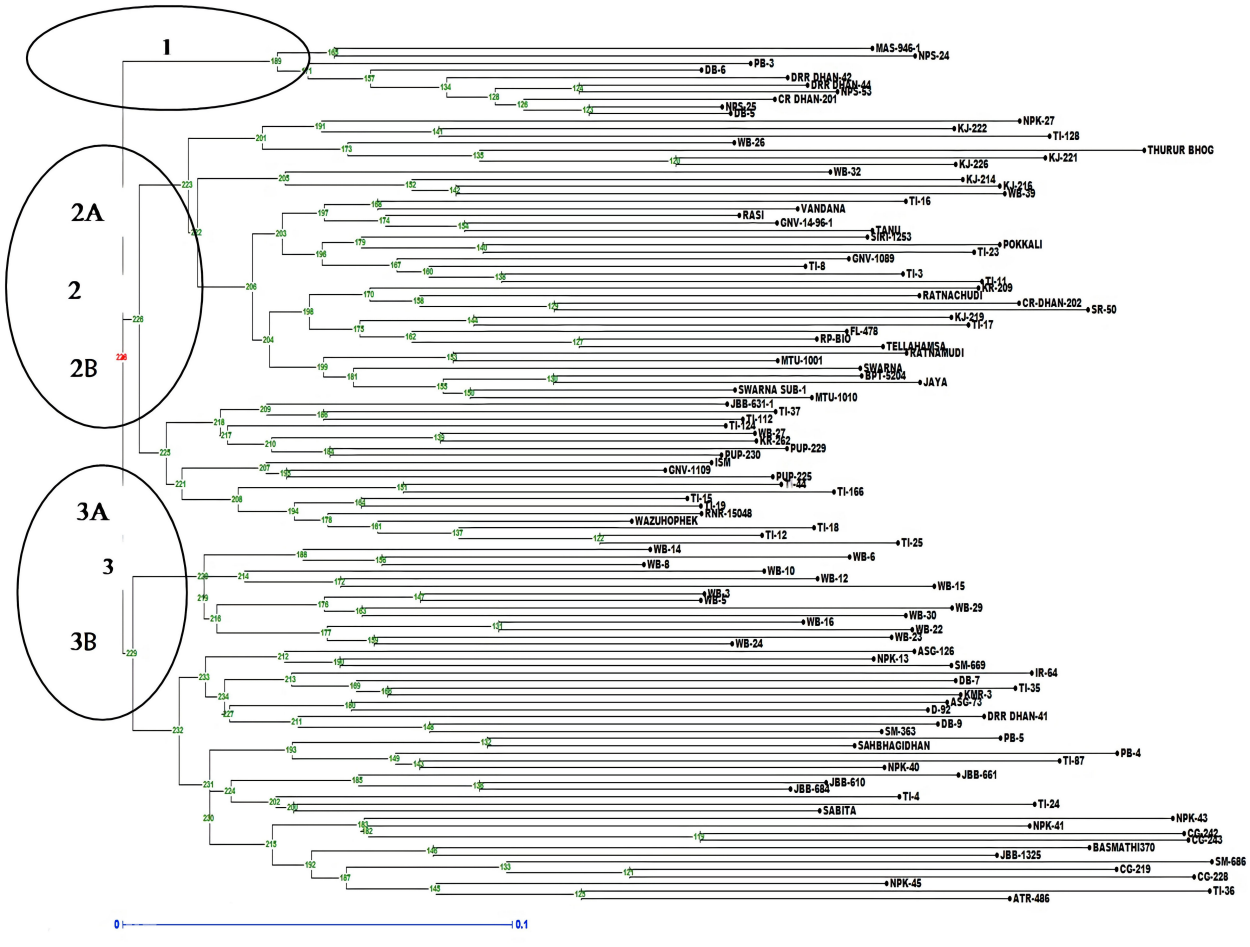
Dendrogram representing clustering of 118 lines constructed by using UPGMA in Darwin 6.0. The numbers above branches represent bootstrap values. The scale indicates the edge length.

The analysis of molecular variance (AMOVA) exhibited genetic variations of 2% among the populations and 98% among individuals of the population (**Supplementary Table S4A**). Shannon index was calculated and Shannon statistics summary is mentioned in **Supplementary Table S4B**. The PIC value in the association panel ranged from 0.0496 (RM280) to 0.7497(RM20698). The number of alleles per loci varied from two to four with an average of two alleles per locus. The highest number of alleles (tetra-allelic) was detected for the six loci viz., RM5933, RM14753, RM20698, RM25310, RM80 and RM13962. Out of the 94 polymorphic SSR markers, 21 were triallelic and 70 were biallelic. The highest PIC values were observed for the markers RM20698 (0.749), RM25310 (0.7480), RM14753 (0.7381) and RM5933 (0.7296) with the mean PIC value of 0.412, more than 37% of markers showed PIC value >0.5 (**Supplementary Table S5**). The diversity analysis clustered the panel into three major clusters having a different number of lines per cluster (**Figure 5**). The PCA and bi-plot also divided the whole population into three components with acceptable Eigen values and Eigen vectors (**Supplementary Figure S7**), which accounted for 31.50% of the total variance.

### 3.5 Population structure

**Figure 6:**
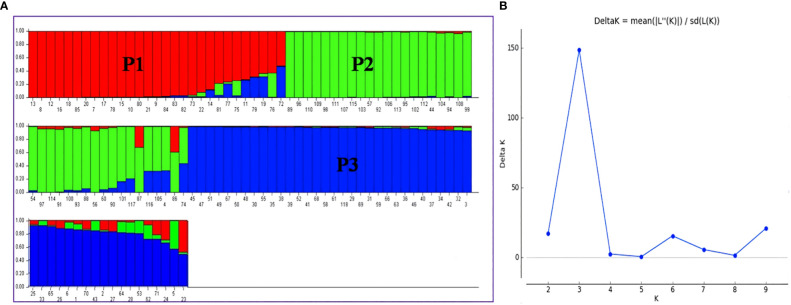
**(A)** Population structure of association mapping in a panel of 118 rice lines (K = 3), **(B)** Graph of ΔK value to the rate of change in the log probability between (−LnPK) successive K values (K = 3). P1, Population 1; P2, Population 2; P3, Population 3. The bar plot represents the sub-populations, where each bar indicates a single line and each color represents the ancestry or recombination. A single bar with single color indicates the same ancestry; a single bar with more than one color indicates admixture/two populations.

When the number of clusters was plotted against ΔK, Model-based population structure showed a sharp peak at K = 3, which displayed three sub-populations in the panel. The highest ΔK of 148.49 and LnP(K) of −13,265.40, as per the Evano table output was obtained in Structure harvester (**Figures 6A, B**). The composition of lines indicated that the association panel was divided into three subpopulations (P1, P2 and P3). Lines with a probability of 80% or higher were regarded pure and assigned to appropriate subgroups, whereas those with a probability of less than 80% were classified as admixture. The fixation index (Fst) for sub-populations P1, P2 and P3 was 0.34, 0.18 and 0.31 with an average value of 0.28 that indicated a moderate population structure with a mean alpha value of 0.14 (**Supplementary Figure S8**). These results coincided with the result of the un-weighted neighbour-joining (NJ) tree and PCA.

### 3.6 Common and consistent marker–trait associations across years and seasons

**Figure 7:**
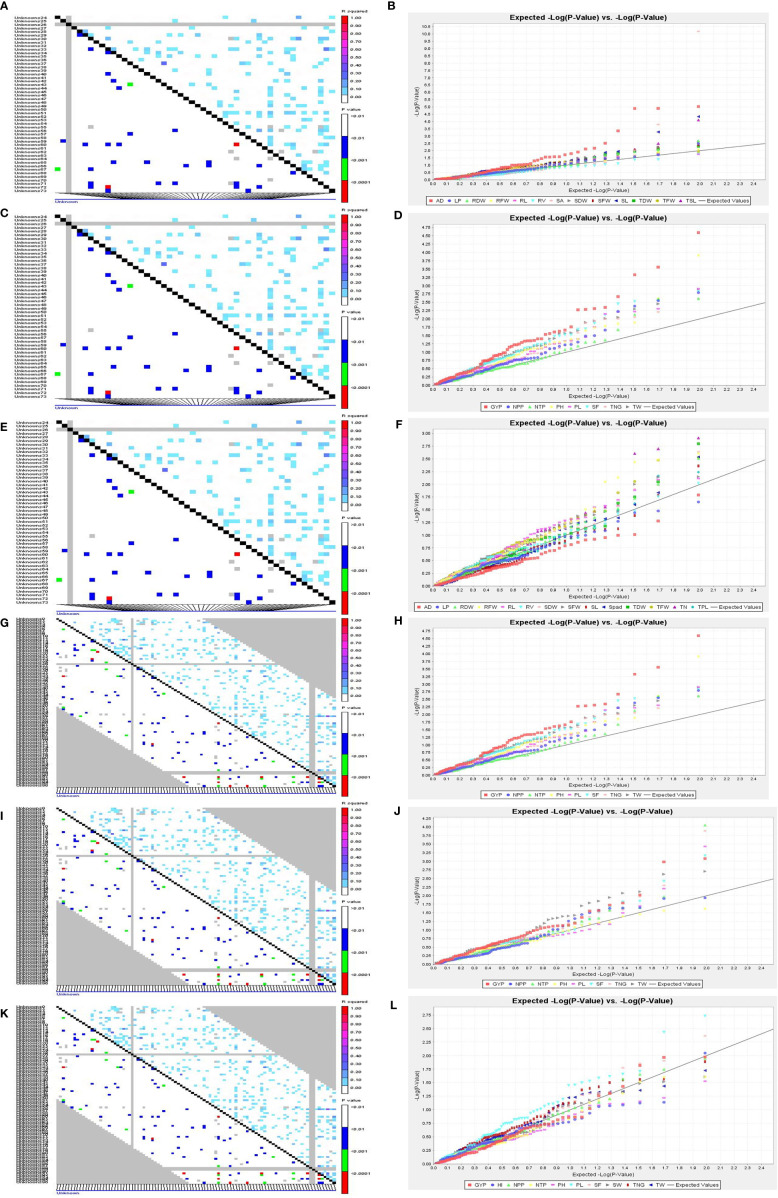
LD and QQ-Plot for SVI traits in the rice association panel. **(A)** LD plot 14 DAS polyhouse, **(B)** QQ-Plot 14 DAS polyhouse, **(C)** LD plot 21 DAS polyhouse, **(D)** QQ-Plot 21 DAS polyhouse, **(E)** LD-plot PI stage aerobic, **(F)** QQ-Plot PI stage aerobic, **(G)** LD-plot irrigated-IIRR, **(H)** QQ-plot irrigated-IIRR, **(I)** LD plot irrigated Dhadesugur, **(J)** QQ-plot irrigated Dhadesugur, **(K)** LD-plot aerobic Dhadesugur, **(L)** QQ-plot aerobic Dhadesugur.

A total of 59 common significant marker–trait associations (MTAs) (R^2^>0.1 with p<0.05) were detected when analyzed by both GLM and MLM, whereas 78 significant MTAs were detected when analyzed only in MLM, in all seedling traits, root- related traits, yield and yield related traits. For SVI with 19 seedling vigour traits at 14 and 21 DAS R^2^ and p-values are given in **Tables 1**, **2** and **Figures 7A–D**. The marker, RM3188 associated with the trait RSA showed the highest R^2^ (0.336), with the corresponding p-value of 2.83E-07 followed by RM80 with RSA (R^2^ = 0.232, p = 0.0019) and RM22961 with SL (R^2^ = 0.192, p = 7.94E-04) at 14 DAS. At 21DAS, RM25310 associated with TDW exhibited the highest R^2^ (0.224) and p (0.010), followed by RM25310 with SDW (R^2 ^ = 0.205, p = 0.018) and RM13962 with RL (R^2 ^= 0.181, p = 0.016). Three common significant MTAs were observed viz. RM22961 with SL and TSL, RM28157 with RLPV and RM1385 with SL consistently at both 14 and 21 DAS during 2018 and 2019.

**Table 1:** Marker–trait associations for SVI 14 DAS under polyhouse 2018 and 2019 (GLM and MLM) (SVI_P).

Sl. No.	GLM						Sl. No.	MLM					
	Trait	Marker	Marker F	Marker p	Perm p	MarkerR^2^		Trait	Marker	F	p	MarkerR^2^	GV
1	RSA	RM3188	21.13493	6.66E-11	0.328	0.35527	1	RAD	RM26558	3.21026	0.01556	0.10322	0.89931
2	RSA	RM80	4.3154	1.59E-04	0.764	0.24008	2	RLPV	RM22961	2.43445	0.0392	0.10085	19.20728
3	TDW	RM25310	2.2362	0.0177	0.789	0.18681	3	RLPV	RM28157	2.9616	0.01025	0.14723	19.20728
4	SL	RM22961	6.15086	4.65E-05	0.028	0.17854	4	RFW	RM14753	2.5216	0.02531	0.12732	499.6454
5	TSL	RM22961	5.85654	7.86E-05	0.027	0.17753	5	RL	RM80	2.21128	0.03211	0.14692	0.12944
6	TFW	RM25310	1.95771	0.0403	0.976	0.1723	6	RSA	RM80	3.33614	0.00192	0.23276	1.11338
7	SFW	RM25310	1.94556	0.04174	0.99	0.1713	7	RSA	RM3188	12.86317	2.83E-07	0.33654	1.11338
8	SDW	RM25310	2.03077	0.03257	0.964	0.16893	8	SDW	RM22961	2.52424	0.03338	0.11002	36.42262
9	RAD	RM25310	2.20081	0.01969	0.953	0.16272	9	SFW	RM18472	2.57904	0.02251	0.13552	252.4174
10	TSL	RM20698	2.65736	0.00813	0.626	0.15553	10	SL	RM20698	2.10208	0.03562	0.1589	2.83089
11	SFW	RM18472	3.226	0.00592	0.62	0.15134	11	SL	RM22961	4.57541	7.94E-04	0.19214	2.83089
12	RLPV	RM28157	3.519	0.00322	0.388	0.14976	12	SL	RM1385	3.39308	0.0069	0.14249	2.83089
13	SL	RM1385	4.80912	5.19E-04	0.142	0.14663	13	TSL	RM20698	2.21079	0.02682	0.16946	3.24901
14	SDW	RM22961	3.95693	0.00246	0.227	0.14561	14	TSL	RM22961	4.32294	0.00126	0.18409	3.24901
15	RL	RM80	2.4547	0.01768	0.863	0.14512	15	TSL	RM1385	2.72335	0.02333	0.11597	3.24901
16	RFW	RM80	2.37701	0.02142	0.778	0.14506							
17	SL	RM20698	2.52872	0.01151	0.746	0.14453							
18	SL	RM25310	1.92229	0.04464	0.99	0.13819							
19	RAD	RM6872	21.48052	9.65E-06	0.029	0.13657							
20	TDW	RM22961	3.55806	0.0051	0.397	0.13597							
21	RAD	RM109	20.86378	1.26E-05	0.034	0.13326							
22	RAD	RM7083	20.77292	1.32E-05	0.035	0.13277							
23	RFW	RM14753	2.87203	0.01233	0.577	0.13122							
24	TSL	RM1385	3.80625	0.00324	0.406	0.12461							
25	TFW	RM18472	2.54613	0.02407	0.917	0.12348							
26	SFW	RM26558	3.54375	0.00926	0.76	0.11367							
27	RAD	RM105	8.26362	4.49E-04	0.338	0.10995							
28	TFW	RM26558	3.36727	0.01219	0.712	0.1087							
29	RLPV	RM22961	2.82053	0.01957	0.852	0.10492							
30	SDW	RM6837	4.37969	0.00593	0.44	0.10032							

Where p<0.05 and R^2^>0.1, GLM, General linear model; MLM, Mixed linear model; Marker-F, F value from the F test on marker; Marker p, p-value from the F test on marker; Perm-p, Permutation p-value from marker; Marker R^2^, R-squared for the marker after fitting other model terms (population structure); GV, genetic variance.

**Table 2:** Marker–trait associations for SVI 21 DAS under polyhouse 2018 and 2019 (GLM and MLM) (SVI_P).

Sl. No.	GLM						Sl. No.	MLM					
	Trait	Marker	Marker F	Marker p	Perm p	MarkerR^2^		Trait	Marker	Marker F	Marker p	MarkerR^2^	GV
1	RLPV	RM28157	3.75069	0.00198	0.287	0.14488	1	RAD	RM18939	3.07787	0.01911	0.10639	0.53662
2	RDW	RM14753	2.49765	0.02657	0.941	0.11797	2	RAD	RM28157	2.2934	0.04015	0.11892	0.53662
3	RDW	RM21165	6.64738	0.00187	0.285	0.10273	3	RLPV	RM28157	3.27571	0.00534	0.16301	85.2746
4	RFW	RM25310	1.93633	0.04287	0.974	0.16062	4	RFW	RM80	2.38921	0.02078	0.16299	182.5298
5	RFW	RM80	2.752	0.00842	0.432	0.1612	5	RFW	RM3029	3.91858	0.01058	0.10024	182.5298
6	RL	RM2584	2.2964	0.03991	0.986	0.11135	6	RL	RM13962	2.38525	0.0169	0.18137	1.73793
7	RL	RM13962	2.13719	0.03251	0.971	0.15237	7	RSA	RM16030	3.81018	0.01212	0.10792	0.80339
8	RSA	RM16030	4.66891	0.00413	0.485	0.10801	8	SDW	RM25310	2.2129	0.01899	0.20552	61.70648
9	SDW	RM25310	2.4332	0.00975	0.638	0.19589	9	SFW	RM80	2.31909	0.02469	0.16188	1146.939
10	SFW	RM26558	4.28449	0.00293	0.225	0.13346	10	SL	RM22961	2.81647	0.01972	0.1201	15.72349
11	SFW	RM25310	2.31569	0.01393	0.694	0.19631	11	SL	RM1385	2.96892	0.01495	0.1266	15.72349
12	SFW	RM80	2.36836	0.02188	0.848	0.15014	12	SL	RM13962	2.22357	0.02594	0.17067	15.72349
13	SL	RM22961	3.84833	0.003	0.232	0.13024	13	TDW	RM25310	2.40735	0.01055	0.22466	84.44329
14	SL	RM25310	2.03078	0.03257	0.958	0.1547	14	TFW	RM80	2.46126	0.0174	0.17079	1702.306
15	SL	RM1385	3.60192	0.00471	0.338	0.12308	15	TSL	RM22961	2.96436	0.01508	0.12767	14.92959
16	SL	RM474	2.48068	0.0275	0.929	0.10514	16	TSL	RM1385	2.77362	0.02131	0.11945	14.92959
17	SL	RM13962	2.18735	0.02852	0.935	0.13705	17	TSL	RM13962	2.08974	0.03678	0.162	14.92959
18	TDW	RM25310	2.64151	0.00514	0.406	0.21028							
19	TFW	RM26558	3.39962	0.01159	0.63	0.10701							
20	TFW	RM25310	2.31806	0.01383	0.688	0.193							
21	TFW	RM80	2.70648	0.00944	0.563	0.16497							
22	TSL	RM22961	3.73787	0.00367	0.29	0.12974							
23	TSL	RM1385	3.09019	0.01199	0.67	0.11005							
24	TSL	RM13962	2.02025	0.04399	0.976	0.13084							

Where p<0.05 and R^2^>0.1, GLM, General linear model; MLM, Mixed linear model; Marker-F, F value from the F test on marker; Marker p, p-value from the F test on marker; Perm-p, Permutation p-value from marker; Marker R^2^, R-squared for the marker after fitting other model terms (population structure); GV, genetic variance.

At the PI stage association of molecular markers with root traits under the polyhouse, conditions were recorded (**Table 3** and **Figures 7E, F**). The marker RM80 associated with TN showed the highest R^2 ^= 0.177 and p = 0.009 followed by RM2584 with R^2 ^= 0.168 and the corresponding p = 0.003, followed by the marker RM410 associated with RL having R^2 ^= 0.121 and p = 0.03 (**Supplementary Figure S9**).

**Table 3:** Marker–trait associations for root traits at panicle initiation (PI) stage under aerobic condition 2019 (GLM and MLM) (Root_PI).

Sl. No.	GLM						Sl. No.	MLM					
	Trait	Marker	Marker F	Marker p	Perm p	MarkerR^2^		Trait	Marker	Marker F	Marker p	MarkerR^2^	GV
1	RLPV	RM1146	2.58457	0.02226	0.813	0.12435	1	RLPV	RM1146	2.21661	0.04682	0.11435	17938.02
2	RLPV	RM2584	2.34583	0.03613	0.935	0.11419	2	RFW	RM28157	2.32844	0.03742	0.12109	45.48182
3	RDW	RM6837	4.89161	0.00313	0.412	0.10347	3	RFW	RM1015	4.06193	0.00883	0.10562	45.48182
4	RDW	RM80	2.06846	0.04529	0.995	0.11965	4	RL	RM410	2.35716	0.03531	0.12124	34.85763
5	RFW	RM6837	4.85361	0.00328	0.431	0.10755	5	RV	RM18472	2.31236	0.03865	0.11688	284.7437
6	RFW	RM28157	3.64684	0.00246	0.371	0.15624	6	SPAD	RM3524	4.14661	0.00794	0.10731	1.67404
7	RFW	RM2584	4.76285	0.00367	0.451	0.10577	7	TN	RM80	2.72174	0.00909	0.17704	1.69878
8	RL	RM26558	3.66568	0.00766	0.513	0.11262	8	TN	RM7006	4.32216	0.00637	0.10543	1.69878
9	RL	RM410	2.6348	0.02009	0.833	0.12226	9	TN	RM2584	3.44641	0.00374	0.16814	1.69878
10	RL	RM80	2.07392	0.0447	0.987	0.12959	10	TPL	RM22961	2.8067	0.02007	0.11764	63.91463
11	SDW	RM6837	5.13683	0.0023	0.186	0.11605							
12	SDW	RM28157	2.86669	0.01247	0.642	0.13078							
13	SFW	RM6837	4.23828	0.00708	0.451	0.10046							
14	SFW	RM12434	4.2179	0.00726	0.46	0.10002							
15	SL	RM22961	2.91498	0.01649	0.748	0.10507							
16	SPAD	RM3524	4.94858	0.00291	0.283	0.11392							
17	SPAD	RM2584	2.52157	0.02531	0.913	0.11866							
18	TDW	RM6837	5.4369	0.00159	0.148	0.11812							
19	TDW	RM28157	2.69452	0.01777	0.821	0.12004							
20	TFW	RM6837	4.93279	0.00297	0.31	0.11372							
21	TFW	RM28157	2.20508	0.04791	0.99	0.1055							
22	TFW	RM2584	2.22918	0.04566	0.988	0.10653							
23	TFW	RM12434	4.83581	0.00335	0.343	0.11175							
24	TN	RM80	3.31911	0.002	0.103	0.18425							
25	TN	RM7006	5.0826	0.00246	0.133	0.11109							
26	TN	RM2584	3.98333	0.00122	0.067	0.16666							
27	TPL	RM22961	3.39601	0.00686	0.461	0.11951							

Where p<0.05 and R^2^>0.1, GLM, General linear model; MLM, Mixed linear model; Marker-F, F value from the F test on marker; Marker p, p-value from the F test on marker; Perm-p, Permutation p-value from marker; Marker R^2^, R-squared for the marker after fitting other model terms (population structure); GV, genetic variance.

The associations of molecular markers with yield and yield-related traits in rice under irrigated conditions at ICAR-IIRR (**Table 4** and **Additional File 2** and **Figures 7G, H**) have been mentioned. The marker RM20698 associated with PH exhibited the highest R^2^ (R^2^ = 0.22751, p = 0.0013), (R^2^ = 0.243, p = 0.0015) for the years 2019 and 2020 respectively, followed by PL with the R^2^ = 0.177 and p = 0.009, the markers RM13962 associated with PH and TNG also showed high R^2^ values 0.167 and 0.157, p-values of 0.025 and 0.038 respectively.

**Table 4:** Marker–trait associations for yield and yield-related traits under irrigated condition at ICAR-IIRR, *wet season* and *dry season* 2020 (GLM and MLM) (Irri_RJN).

Sl. No.	GLM						Sl. No.	MLM					
	Trait	Marker	Marker F	Marker p	Perm p	MarkerR^2^		Trait	Marker	Marker F	Marker p	MarkerR^2^	GV
1	GYP	RM6837	8.86706	2.56E-05	0.004	0.17821	1	GYP	RM410	2.39519	0.03271	0.11844	35.60364
2	GYP	RM5933	3.94436	0.00497	0.461	0.11564	2	GYP	RM5501	5.01414	0.00268	0.12397	35.60364
3	GYP	RM14753	2.29204	0.04026	0.978	0.10412	3	GYP	RM20069	7.57927	8.18E-04	0.12493	35.60364
4	GYP	RM20698	2.20068	0.02754	0.936	0.14629	4	GYP	RM3825	4.22464	0.0072	0.10445	35.60364
5	GYP	RM1385	3.5433	0.00524	0.48	0.12888	5	GYP	RM13962	1.9739	0.04953	0.14641	35.60364
6	GYP	RM410	3.35568	0.00452	0.442	0.14485	6	NPP	RM1388	4.28439	0.00668	0.1078	1.40989
7	GYP	RM5501	6.86326	2.77E-04	0.043	0.14424	7	NPP	RM2584	2.28469	0.04086	0.11497	1.40989
8	GYP	RM80	2.31903	0.02469	0.914	0.13731	8	NTP	RM1388	4.08396	0.00859	0.10362	1.9032
9	GYP	RM1141	8.21119	4.70E-04	0.071	0.11788	9	PH	RM3455	4.84003	0.00333	0.12078	273.0405
10	GYP	RM3825	5.20118	0.00213	0.258	0.11361	10	PH	RM14753	2.27828	0.04139	0.11371	273.0405
11	NPP	RM1388	5.42196	0.00162	0.153	0.10453	11	PH	RM18939	3.15382	0.01698	0.10494	273.0405
12	NTP	RM1388	5.08432	0.00246	0.204	0.10297	12	PH	RM20698	3.25677	0.00158	0.24382	273.0405
13	PH	RM20698	4.18155	1.23E-04	0.008	0.22744	13	PH	RM1385	3.24193	0.00909	0.13484	273.0405
14	PH	RM1385	3.96276	0.00243	0.159	0.13259	14	PH	RM13962	2.23318	0.02529	0.16719	273.0405
15	PH	RM13962	2.27604	0.02259	0.852	0.14072	15	PL	RM20698	2.61923	0.00902	0.1778	1.76131
16	PL	RM20698	2.70444	0.00716	0.53	0.13802	16	PL	RM2584	2.35175	0.0357	0.10643	1.76131
17	PL	RM2584	3.20855	0.00614	0.475	0.11095	17	SF	RM3455	4.03116	0.00918	0.10441	23.49171
18	SF	RM1385	3.0194	0.01364	0.832	0.10387	18	SF	RM16030	4.31541	0.00642	0.11177	23.49171
19	SF	RM16030	4.9981	0.00274	0.428	0.10161	19	TNG	RM229	4.17365	0.00767	0.10622	560.2655
20	SF	RM2584	2.38869	0.03314	0.966	0.10003	20	TNG	RM12434	4.13802	0.00803	0.10532	560.2655
21	TNG	RM410	2.83614	0.01328	0.668	0.11597		TNG	RM13962	2.06756	0.03895	0.15787	560.2655
22	TNG	RM474	2.50286	0.02629	0.897	0.104							
23	TNG	RM229	5.07743	0.00248	0.181	0.10281							

Where p<0.05 and R^2^>0.1, GLM, General linear model; MLM, Mixed linear model; Marker-F, F value from the F test on marker; Marker p, p-value from the F test on marker; Perm-p, Permutation p-value from marker; Marker R^2^, R-squared for the marker after fitting other model terms (population structure); GV, genetic variance.

The association of molecular markers with yield and yield-related traits in rice under irrigated conditions at Dhadesugur (**Table 5**; **Figures 7I, J**) showed the highest association for the traits. The highest association was observed for the traits NPP, SF, and TNT with the markers RM25310 (R^2^ = 0.189, p = 0.03), RM18472 (R^2^ = 0.174, p = 0.004) and RM2584 (R^2^ = 0.163, p = 0.005) respectively. The association of molecular markers with yield and yield-related traits in rice under aerobic conditions at Dhadesugur is given in **Table 6**; **Figures 7K, L**. A total of four significant MTAs were identified among them RM22961 and RM1146 were associated with GYP (R^2^ = 0.101, 0.128 and p-value = 0.042, 0.025 respectively), RM4455 with PL (R^2^ = 0.114 and p-value = 0.005) and RM3188 with SF (R^2^ = 0.101 and p-value = 0.01). The marker RM1146 showed the highest R^2^ value of (0.128) with GYP. The Q-Q plot, Manhattan plot and LD plots obtained from TASSEL 4 also confirmed the significant association of markers with the traits. The Q-Q plot distribution depicted that the data is symmetric and the distributions of p-values according to the traits were normally distributed and also considered the type of error (Type I and II) in deciphering the MTAs.

**Table 5:** Marker–trait associations for yield and yield-related traits under irrigated condition at Dhadesugur *wet season* 2020 (GLM and MLM) (Irri_DSG).

Sl. No.	GLM						Sl. No.	MLM					
	Trait	Marker	Marker F	Marker p	Perm p	MarkerR^2^		Trait	Marker	Marker F	Marker p	MarkerR^2^	GV
1	GYP	RM14472	7.56083	8.32E-04	0.284	0.10747	1	NPP	RM20698	1.98949	0.0476	0.15485	0.84387
2	GYP	RM105	7.30732	0.00104	0.323	0.10429	2	NPP	RM22961	3.29904	0.00819	0.14265	0.84387
3	GYP	RM1146	2.73283	0.01643	0.868	0.11909	3	NPP	RM25310	1.99429	0.03624	0.18972	0.84387
4	NPP	RM22961	3.10674	0.01164	0.792	0.11944	4	NPP	RM1015	4.44792	0.00544	0.1154	0.84387
5	NPP	RM25310	2.15676	0.02245	0.907	0.17926	5	NTP	RM2584	3.29247	0.00516	0.16356	2.27814
6	NPP	RM2584	2.87563	0.01224	0.805	0.1319	6	PL	RM22961	2.98303	0.01457	0.12913	0.42875
7	NTP	RM17263	2.74903	0.02227	0.924	0.1043	7	SF	RM18472	3.33162	0.00475	0.17432	1.0667
8	NTP	RM25310	1.90009	0.04759	0.995	0.15712	8	SF	RM1015	3.96768	0.00994	0.1038	1.0667
9	NTP	RM2584	5.25338	8.79E-05	0.14	0.21042	9	SF	RM2584	2.58309	0.02233	0.13515	1.0667
10	PH	RM25310	2.13132	0.02422	0.863	0.17923	10	TNG	RM5179	5.75065	0.00108	0.14754	96.61729
11	PL	RM22961	3.44388	0.00629	0.576	0.13344							
12	PL	RM105	8.48689	3.70E-04	0.058	0.12873							
13	SF	RM18472	4.26036	6.85E-04	0.085	0.18837							
14	SF	RM1015	4.73452	0.0038	0.324	0.11166							
15	SF	RM2584	2.57966	0.02248	0.884	0.12337							
16	TNG	RM5179	7.48957	1.30E-04	0.044	0.158							
17	TNG	RM229	4.50668	0.00506	0.561	0.10191							
18	TW	RM6837	5.26166	0.00197	0.145	0.10283							

Where p<0.05 and R^2^>0.1, GLM, General linear model; MLM,Mixed linear model;Marker-F, F value from the F test on marker; Marker p, p-value from the F test on marker; Perm-p, Permutation p-value from marker; Marker R^2^, R-squared for the marker after fitting other model terms (population structure); GV, genetic variance.

**Table 6:** Marker–trait associations for yield and yield-related traits under aerobic condition at Dhadesugur *wet season* 2020 (GLM and MLM) (Aero_DSG).

Sl. No.	GLM						Sl. No.	MLM					
	Trait	Marker	Marker F	Marker p	Perm p	MarkerR^2^		Trait	Marker	Marker F	Marker p	MarkerR^2^	GV
1	GYP	RM1146	2.9431	0.01065	0.613	0.12808	1	GYP	RM22961	2.39035	0.04241	0.10134	1.99231
2	PL	RM80	2.41233	0.01963	0.861	0.145	2	GYP	RM1146	2.52764	0.025	0.1286	1.99231
3	PL	RM4455	5.31786	0.00184	0.211	0.1183	3	PL	RM4455	4.44564	0.00546	0.11479	3.75344
4	SF	RM3188	4.63045	0.00433	0.31	0.1074	4	SF	RM3188	3.90219	0.0108	0.10113	38.55806
5	SW	RM2584	2.39296	0.03285	0.949	0.11677							
6	TNG	RM25310	2.33883	0.01299	0.674	0.19182							
7	TNG	RM410	2.25913	0.043	0.977	0.10703							
8	TW	RM474	2.21555	0.04692	0.978	0.10539							

Where p<0.05 and R^2^>0.1, GLM, General linear model; MLM,Mixed linear model; Marker-F, F value from the F test on marker; Marker p, p-value from the F test on marker; Perm-p, Permutation p-value from marker; Marker R^2^, R-squared for the marker after fitting other model terms (population structure); GV, Genetic variation.

Seven significant MTAs were consistent over the years 14 DAS in 2018 and 2019 for SVI under polyhouse conditions the marker RM25310 was significantly associated with the trait GP and TDW, RM2584 with GP, markers RM14753 and RM80 were significantly associated with the trait RFW, RM22961 was significantly associated with the traits SDW, SL, SVI, TSL, RM18472 with SFW, TFW and the marker RM1385 was significantly associated with the traits SVI-I, TSL. There were four significant MTAs which were consistent over the years for 21 DAS in 2018 and 2019 SVI-II under polyhouse conditions. The marker RM25310 was significantly associated with the traits GP, SDW, SVI-II, TDW and TFW. The marker RM80 was associated with the RFW, SFW and TFW, RM22961 with SL, SVI-I, TSL and RM1385 was significantly associated with the traits SL and TSL. Four significant MTAs were consistent over years both 14 and 21 DAS in 2018 and 2019 for SVI under polyhouse conditions. The marker RM25310 was significantly associated with the traits GP and TDW. The marker RM80 was associated with the RFW, RM22961 with SL, SVI-I, TSL and the marker RM1385 was significantly associated with TSL (**Additional File 2**).

Two MTAs viz., RM410 associated with GYP and RM20698 associated with PH were consistent over the years under irrigated conditions at IIRR. Two MTAs viz., RM2584 associated with spikelet fertility and RM5179 associated with TNG were consistent under irrigated conditions at IIRR and DSG. The marker RM2584 was significantly associated with the trait SW and GYP under aerobic and irrigated conditions at DSG respectively (**Additional File 2**).

### 3.7 *In silico* identification of genes spanning 1Mb of highly significant markers

In total eighteen markers associated with more than two traits in GLM and MLM were selected for *in-silico* identification of genes spanning the 1 Mb region upstream and downstream of the marker (**Table 7**). The maximum significant MTAs were found on the chromosomes number 2, 3 and 12.

**Table 7:** *In-silico* identification of genes spanning 1 Mb of highly significant marker (having two or more associations).

Sl. No.	Marker name	Chr no. and Marker position	Identified QTLs	Traits	Locus IDs Functional annotation
1	RM5501	1_35	*qRL*1.4-NERICA7	GYP_Irri_RJN, SFW_ 21SVI_P	LOC_Os01g59760.1 bZIP transcription factor, modulation of the floral transition, floral repressor; HBF2
					LOC_Os01g60200.1 WD40 repeat-like domain containing protein.
					LOC_Os01g60230.1 OsPILS6a Auxin efflux carrier domain containing protein
					LOC_Os01g60600.1 WRKY108 WRKY transcription factor, promotion of phosphate accumulation under Pi-replete conditions
					LOC_Os01g61010.1 Nodulin-like domain-containing protein.; Similar to nodulin-like protein.
					LOC_Os01g61044.1 Amino acid transporter, transmembrane domain containing protein.
2	RM1385	2_26.67		GYP, SF, PH_Irri_RJN, SL, TSL_14 and 21SVI_P	LOC_Os02g43510.1 GDP-L-fructose synthase 1, putative, expressed
					LOC_Os02g43960.1 Expressed protein
					LOC_Os02g44136.1 Retrotransposon protein, putative, Ty1-copia subclass, expressed
					LOC_Os02g44560.1 C2 domain containing protein, putative, expressed
3	RM13962	2_31.37	*qSLA*15-2,1	RL, SL, TSL_21SVI_P, PH, GYP, TNG_ Irri_RJN	LOC_Os02g51170.1 Expressed protein
					LOC_Os02g51200.1 Retrotransposon protein, putative, LINE subclass, expressed
					LOC_Os02g51230.1 Transposon protein, putative, CACTA, En/Spm sub-class, expressed
4	RM3188	2_3.45	Root length	RSA_14SVI_P, SF_Aero_DSG	LOC_Os02g06860.1 OsMADS80 - MADS-box family gene with M-alpha type-box, expressed
					LOC_Os02g06950.1 No apical meristem protein, putative, expressed
					LOC_Os02g06720.1 WD domain containing protein, putative, expressed
					LOC_Os02g06120.1 Leucine Rich Repeat family protein, expressed
5	RM14753	3_9.58	*qSH*45-3,1, *qAGR*45-3,1,*Qrgr*45-3-1	RFW_14SVI_P, GYP, PH_Irri_RJN	LOC_Os03g16320.1 Expressed protein
					LOC_Os03g17330.1 Expressed protein(Post-emergence inflorescence)
6	RM16030	3_32.7	*qDRD*3.1	RSA_21SVI_P, SF_Irri_RJN	LOC_Os03g57180.1 Expressed protein
					LOC_Os03g57360.1 Expressed protein
7	RM6837	3_24	*qRL*1.4-NERICA7	SDW_14SVI_P, RDW, RFW, SDW, SFW,TDW, TFW_Root_PI,TW-Irri_DSG,GYP_Irri_RJN	LOC_Os03g13640.1 Expressed protein
					LOC_Os03g13579.1 Expressed protein
					LOC_Os03g13690.1 Expressed protein
8	RM1388	4_25	Root length density	NPP, NTP_Irri_RJN	LOC_Os04g43590.1 Transposon protein, putative, CACTA, En/Spm sub-class
					LOC_Os04g42550.1 Expressed protein
					LOC_Os04g43324.1 G-patch domain containing protein, putative, expressed
					LOC_Os04g43450.1 Expressed protein
					LOC_Os04g43590.1 Transposon protein, putative, CACTA, En/Spm sub-class
					LOC_Os04g43630.1 Retrotransposon protein, putative, unclassified
					LOC_Os04g42350.1 Heavy metal-associated domain containing protein, expressed
9	RM18472	5_16.64		SFW, TFW_14SVI_P, SF_Irri_DSG	LOC_Os05g27700.1 Transposon protein, putative, CACTA, En/Spm sub-class, expressed
					LOC_Os05g28450.1 Minor ampullate silk protein MiSp1, putative, expressed
					LOC_Os05g28540.1 Retrotransposon protein, putative, Ty3-gypsy subclass, expressed
					LOC_Os05g26040.1 Pumilio-family RNA binding repeat containing protein, expressed
					LOC_Os05g28050.1 Expressed protein
10	RM2584	8_7.56	Root weight	TN,RLP,RFW,SPAD,TFW_ Root _PI,NPP,NTP,SF-Irri_DSG, PL,SF,NPP Irri_RJN	LOC_Os08g11640.1 Expressed protein
					LOC_Os08g12740.1 NB-ARC domain containing protein
					LOC_Os08g13390.1 DUF537 family protein
					LOC_Os08g13420.1 Domain receptor-like kinase 56
11	RM22961	8_16.6		TPL, SL _ Root _PI, TSL, SL-14 and 21SVI_P, SDW, TDW, RLP-14SVI_P, GYP-Aero_ DSG, NPP,PL-Irri_DSG	LOC_Os08g27870.1 EARLY flowering protein, putative, expressed
					LOC_Os08g27759.1 Expressed protein
					LOC_Os08g27500.1 Retrotransposon protein, putative, Ty1-copia subclass, expressed
					LOC_Os08g27674.1 LTPL130 - Protease inhibitor/seed storage/LTP family protein precursor, putative, expressed
12	RM80	8_24	Root length density	RDW,RL,TN,RDW_Root_PI, RFW,SFW,TFW_21SVI_P, RFW,RL,RSA-14SVI_P	LOC_Os08g38370.1 Zinc finger CCCH domain-containing protein 57
					LOC_Os08g38460.1 Ring-H2 finger protein Drought and salt stress tolerance
					LOC_Os08g38590.1 MADS BOX GENE 62
13	RM410	9_18	*qDRL*9	RL_Root_PI, TNG, GYP_Irri_RJN	LOC_Os09g28830.1 OsSCP42 - Putative Serine Carboxypeptidase homologue, expressed
					LOC_Os09g28860.1 Late embryogenesis abundant protein D-34, putative, expressed
					LOC_Os09g29550.1 dof zinc finger protein 2, putative
14	RM25310	10_11.78		SL TDW,TFW,RFW,SDW,SFW-21SVI_P, NPP,NTP,PH_Irri_ DSG	LOC_Os10g22960.1 Alpha/beta hydrolase fold-1 domain containing protein
					LOC_Os10g24370.1 Cyclin-like F-box domain-containing protein
					LOC_Os10g22930.1 Leucine-rich repeat domain-containing protein
15	RM229	11_18.4	*qDRL*11	RSA_SVI_P, TNG_Irri_RJN	LOC_Os12g30190.1 Expressed protein
					LOC_Os12g29450.1 Retrotransposon protein, putative, LINE subclass, expressed
16	RM3455	12_5	*qRL*1.4-NERICA7	PH, SF_Irri_RJN	LOC_Os12g09340.1 Hypothetical protein
					LOC_Os12g09370.1 Expressed protein
17	RM28157	12_17.44		RAD-21SVI_P, RLP_14 and 21SVI_P, SDW, TDW, RFW,TFW_Root_PI	LOC_ Os12g29330.1 No apical meristem (NAM) protein domain-containing protein
					LOC_ Os12g29330.1 NAC domain-containing protein 139
					LOC_Os12g29560.1 DHHC domain protein 30
18	RM1015	12_22	*qRL*1.4-NERICA7	RFW_Root_PI, SF, NPP_Irri_DSG	LOC_Os12g36530.1 Patatin, putative, expressed
					LOC_Os12g36590.1 Retrotransposon protein, putative, unclassified, expressed
					LOC_Os12g36710.1 Expressed protein

SVI_P-SVI under polyhouse, Root_PI-PI stage aerobic, Irri_RJN-Irrigated condition Rajendranagar,Irri_DSG- Irrigated condition Dhadesugur, Aero_DSG- Aerobic condition Dhadesugur.

**Figure 8:**
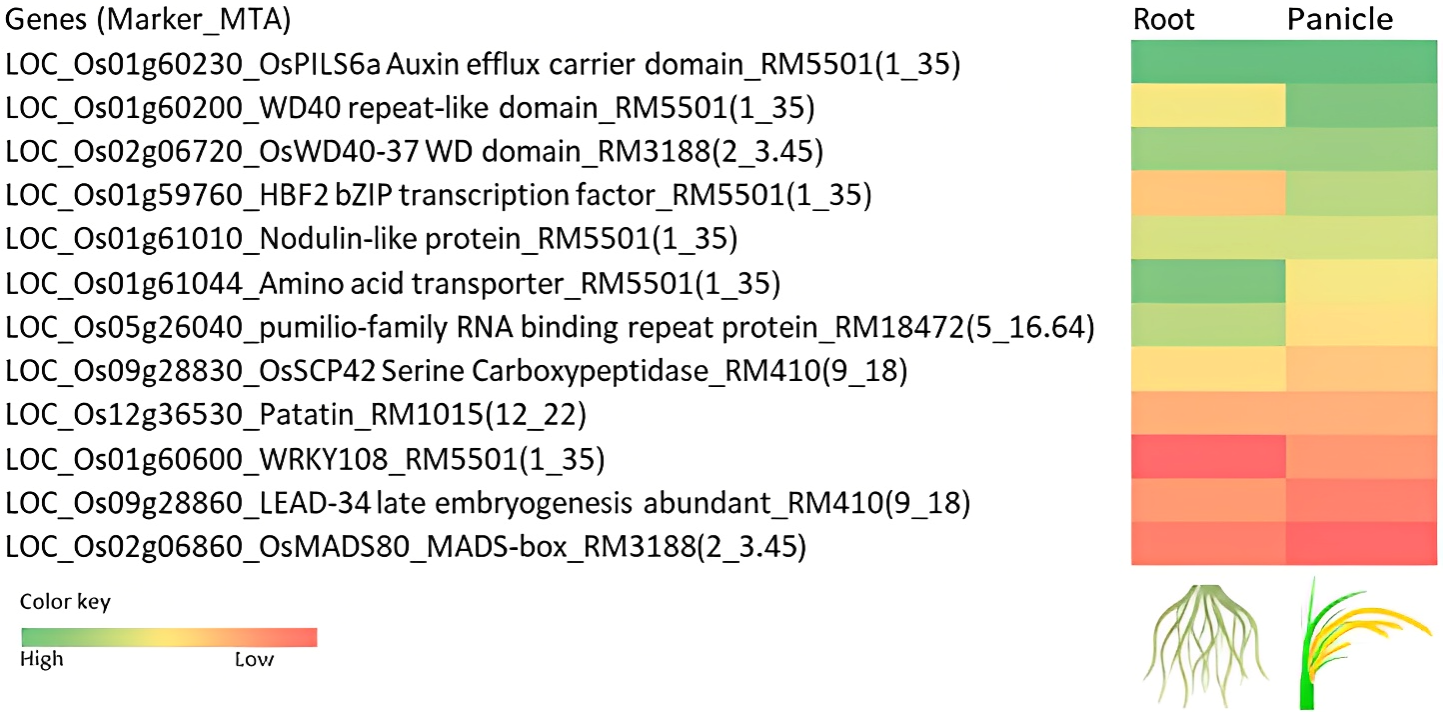
Heat map showing the scores based on the *in-silico* expression of genes associated with the marker–trait associations (MTAs) in the root and panicle tissues. The *in-silico* expression is based on the rice expression databases viz., Rice Expression Database (RED) IC4R (http://expression.ic4r.org/), RiceXpro (https://ricexpro.dna.affrc.go.jp/), RGAP Rice Genome Annotation Project (http://rice.uga.edu/cgi-bin/ORF_infopage.cgi), RGI Rice Gene Index (https://riceome.hzau.edu.cn), NetREx Network-based Rice Expression Analysis (https://bioinf.iiit.ac.in/netrex/index.html), RAPDB The Rice Annotation Project Database (https://rapdb.dna.affrc.go.jp/search/).

The gene information was fetched from all the databases for root and panicle tissues at seedling and reproductive stages (**Additional File 3**). Based on the functional relevance of the genes, twelve genes were shortlisted and proposed to be promising candidates associated with the traits recorded in the present study (**Figure 8**).

The marker RM5501(1_35) had six functionally significant genes encoding, *HBF2 bZIP* transcription factor involved in the modulation of the floral transition, *WD40* repeat-like domain-containing protein, *OsPILS6a* Auxin efflux carrier domain-containing protein, *WRKY108* WRKY transcription factor, Promotion of phosphate accumulation under Pi-replete conditions, nodulin-like domain-containing protein, amino acid transporter, transmembrane domain-containing protein. The gene *LOC_Os01g60230* encoding *OsPILS6a*, a *PIN*-like gene family of rice auxin and cytokinin has been reported as a floral transition in rice. It displayed upregulation upon stress induction, expressed in roots (in the elongation zone) after drought induction. The gene, *LOC_Os01g61044* encoding the amino acid transporter has been associated with the number of spikelets per panicle under direct seeding (Rebolledo et al., 2016). This gene has also been reported to express after drought and ABA, cytokinin treatment. The marker RM14753(3_9.58) associated with seedling vigor index had expressed protein and the *in silico* databases showed upregulation of the gene in root under abiotic stress. The pumilio-family RNA binding repeat-containing protein (RM18472(5_16.64), SFW, TFW_14SVI_P, SF_Irri_DSG), *OsSCP42* serine carboxypeptidase (RM410(9_18), RL_Root_PI, TNG, GYP_Irri_RJN), *patatin* (RM1015(12_22) (RFW_Root_PI, SF, NPP_Irri_DSG) have been reported to be upregulated upon ABA treatment, drought, cold, and osmotic stresses. These genes are associated with the root traits at seedling and reproductive stages and can be further explored for gene expression in roots. The amino acid transporter (GYP_Irri_RJN) has been recorded to be expressed in roots, panicle tissues, and upon induction of abiotic stresses. The *OsWD40-37 WD* domain-containing protein (RM3188(2_3.45)) is proposed to be a promising candidate for further studies related to yield under aerobic conditions.

## 4 Discussion

The present study indicated the presence of significant genetic variability and a strong positive correlation for seedling vigour traits, root traits, and yield and yield-related traits at all the studied environments and conditions, revealing that sufficient variability was present in the association panel (Suralta et al., 2010; Dhanwani et al., 2013; Chen et al., 2019). Highly variable traits are preferred in breeding programmes for maximizing the genetic base (Barde et al., 2021). High GCV and PCV coupled with high heritability and high GAM were observed for maximum studied traits, the heritability of broad-sense (*h^2^* bs) was divided into three categories: high (above 60%), medium (30%–60%), and low (less than 10%). (Robinson et al., 1949). GAM is also divided into three categories: high (>20%), medium (10–20%), and low) (below 10 %) (Johnson et al., 1955). Estimates of genetic advance as per cent of mean (GAM) provide more trustworthy information on the effectiveness of selection in trait improvement (Islam et al., 2015). Broad sense heritability contains both additive and epistatic effects; it will only be trustworthy when combined with a high level of genetic advance. In forecasting the efficiency of selection, heritability estimates combined with genetic advances are more useful than heritability alone (Dhanwani et al., 2013). High GCV and PCV coupled with high heritability and high GAM were observed for the traits like the number of tillers per plant, the number of panicles per plant and grain yield per plant, these traits were controlled by additive gene action, which could be improved through simple selection methods. The characters with a high GCV and PCV, as well as a high heritability and GAM, indicate that the character is driven by additive gene action and the selection could be beneficial in improving it (Amegan et al., 2020; Demeke et al., 2023). The narrow difference between GCV and PCV implies that most features are less influenced by the environment. The heritability estimates for a given feature determine the reliability of the phenotypic value. As a result, high heritability aids in the efficient selection of a specific trait, hence quantitative trait genetic analysis is critical for breeding programmes.

Breeding for seedling vigour has been a major breeding target in rice and other crops under DSR (Zhao et al., 2006). Relatively, till now, the breeding efforts for aerobic dry direct seeded rice is sparse as compared to other traits in rice (Xu et al., 2020). Hence, the rice breeders need to exploit appropriate genetic differences among rice germplasm to determine the magnitude of genetic variation and to dissect the genetic basis of seedling vigour. Earlier reports on seedling vigour used higher germination percentage as an indicator of good seedling vigour. Seedling vigour with quantitative inheritance traits is significantly influenced by morphological, physiological and biochemical traits (Mahender et al., 2015). Correlated traits such as seedling height, seedling dry weight, rapid shoot growth, shoot dry weight, shoot length, coleoptile length and root length have been identified (Fujino et al., 2008; Trachsel et al., 2010; Yang et al., 2010; Singh et al., 2017). The correlation study identified a positive association between shoot dry weight and root dry weight 14 and 28 days (Kato and Okami, 2010; Anandan et al., 2022). Germination rate, root activity, leaf area and chlorophyll content (Cairns et al., 2009; Wang et al., 2010; Dang et al., 2014) have been reported. Rapid germination (Xie et al., 2014), anaerobic germination (Septiningsih et al., 2013) and shoot development improves seedling establishment (Lee et al., 2012) under direct seeding situations. The relative water content (RWC) of the susceptible and shallow rooting genotype reduced to 72% as compared to the tolerant and deep rooting genotype Azucena which showed the RWC of 80% when exposed to the 14 days water stress treatment (Abdirad et al., 2022).

Based on our findings, the lines viz., Langphou, Mouli, TI-128, JBB-631-1, Ratnachudi and Tellahamsa showed promising performance for seedling vigour traits. Lines namely, NPK-45, Moirangphou-Angouba, JBB-661, Wangoo-Phou, GNV-1109, NPK-13 and Dissi showed promising performance for root-related traits. Similarly, lines viz., KR-209, Pat-Phou, JBB-631-1, SM-363, Wangoo-Phou, PB-3 and MTU1010 showed promising performance for yield and yield-related traits under irrigated conditions. The lines Phouren, Ratnamudi, NPK-43, TI-112, TI-87, JBB-631-1 and JBB-684 showed promising performance for yield traits under aerobic conditions. From the overall field experiments considering all the environments and all the locations the lines Langphou, KR-209, JBB-631-1, Akut-Phou, TI-36, GNV-14-96-1 and NPK-43 exhibited consistent performance for yield and yield-related traits like GYP, TW, SF and NPP over the checks.

Genetic diversity is the key determinant of germplasm utilization in crop improvement. Population with a high level of genetic variation is a valuable resource for broadening the genetic base in any breeding program. The lines used in this study should have a complex breeding history, from diverse genetic backgrounds (Pandit et al., 2017; Sahu et al., 2020; Yadav et al., 2021). In this study, the landraces from North-Eastern India showed considerable features for root length, root volume and yield. Such Indian landraces harbour alleles of economic importance and can be explored further. The landraces are considered gold mines for improving economically important agronomic traits as they are adapted to local environments without any additional input while cultivating. They harbour regions that have not been utilized in the breeding programs and whatever association is expected is because of their evolutionary history and recombination (Rao et al., 2018). The North-Eastern landraces have been phenotyped for the very first time for the root traits under aerobic conditions and present a novel genetic source for utilizing in breeding programs for developing climate-resilient rice varieties.

The mutant lines, and introgression lines identified for yield (based on earlier reports) were selected for exploring the traits desirable for the aerobic system of cultivation, those lines also had desirable traits as well as co-relation with yield. The marker–trait associations reveal that robust MTAs identified could serve as a valuable resource for enhancing seedling vigour, root traits and yield-related traits indicating their feasibility to use in the marker-assisted breeding programme. We considered majorly the associations identified by the MLM over the associations identified by the GLM (Yu et al., 2006). The MLM considers both population structure and kinship, which is more suitable over GLM, where only population structure is considered for analyses especially when there is no prior information regarding the lines in the association panel (Rao et al., 2018). The false positives were well controlled by the MLM model which indicated the robustness of results obtained through statistical analysis. The significant SSR markers associated with the (R^2^ of >0.1 and p-value < 0.05) with more than two phenotypic traits were selected for searching the nearby genes in 1Mb spanning region. The genes identified were consistent with the associated phenotypes. Among the significant MTAs, six hypervariable markers viz., RM1385, RM18472, RM18939, RM22961, RM25310 and RM28157 on the chromosome numbers 2, 5, 5, 8, 10 and 12 respectively were found to be novel for the identified genes. Our results correlate with earlier report by Xu et al. (2020) and Zhang et al. (2020) where four QTLs have been identified on chromosomes 3, 6, and 8 whereas, two QTLs (*LOC_Os03g08880* and *LOC_Os06g13060*) were linked to maximum root length and two were related to total root weight. The marker RM22961 associated with traits TSL and SL at the seedling stage at 14 and 21 DAS and also associated with TPL and SL at the PI stage and with grain yield per plant under aerobic conditions. Khodaeiaminjan et al., 2023 identified the MTAs viz., ‘BOPA1_1582-63’ on chromosome 5H for root/shoot ratio and ‘SCRI_RS_192761’ on chromosome 3H for shoot dry weight for control and osmotic stress conditions. In phenotypic correlation analysis also the trait total seedling length showed a significant correlation with the seedling vigour index traits. The marker RM22961 with the seedling height and grain yield trait under aerobic conditions can be taken up as a strong marker–trait association of this marker. In our study markers RM2069 associated with PL and NPP, and RM2584 with PL, NPP and NTP and SF for yield-related traits under the irrigated condition at two different locations at ICAR-IIRR and Dhadesugur respectively. However, consistent expression of these two markers can be strong evidence for association with the particular yield contributing traits hence these markers can be directly used for the selection of yield traits under irrigated conditions. The marker RM25310 was significantly associated with GP and TDW, RM2584 with GP, RM14753 and RM80 with RFW, RM22961 with SDW, SL, SVI-I, TSL, RM18472 with SFW, TFW and RM1385 with the traits SVI-II, TSL were consistent over year for seedling traits at 14DAS. The marker RM25310 was significantly associated with GP and TDW, RM80 with the RFW, RM22961 with SL, SVI-II, TSL and the marker RM1385 with the traits TSL were consistent over the year for seedling traits at 21DAS. The marker RM410 was significantly associated with the trait GYP and RM20698 with PH were consistent over the year for yield traits under irrigated conditions, whereas the marker RM2584 was significantly associated with the trait SW and GYP under the aerobic and irrigated condition at DSG respectively. The *in-silico* analysis identified abiotic stress-related genes viz., NAM, LEA, MADS, NAC, bZIP, WRKY, RING finger, spanning the nearby regions of the markers implying high co-relation of the marker with the root and yield traits contributing to aerobic adaptation, whereas Abdirad et al. (2022), identified the genes involved in root development and drought tolerance were identified viz., *OsbHLH120* for root thickness, *OsNAC10* for root thickness and drought tolerance, *OsPHR3* for LR development, *PIP1;3/RWC3* for root length and water stress avoidance, *OsMADS18* for root elongation, and *OsNLA1* for root length and growth through transcriptome study. Anandan et al., 2022., identified two important peptide transporters (PTR5 and PTR6) involved in mobilizing nitrogen in the root during the early vegetative stage on chromosome 4, (30 Mb), and at 14 days. A novel QTL from 21.12 to 21.46 Mb on chromosome 7 with two linkage disequilibrium (LD) blocks governing root length was identified. Rebolledo et al. (2016) identified six MTAs associated with panicle architecture and the number of spikelets per panicle, *NECKLEAF1 (nl1)* associated with *NSPexp1*. *OsMADS6-5* was present within the LD block of q-25 which controls the floral organ identity and flowering time. Further linked markers can be utilized for aerobic breeding programs for the selection of SVI, root yield and yield-related traits under aerobic and irrigated conditions. The shortlisted promising candidates genes associated with the yield and root-related traits can be further analysed for quantitative gene expression and functional cloning.

## 5 Conclusion

The present study is novel and the first of its kind in India wherein the association panel has been studied concomitantly for seedling vigour, root traits, as well as yield and yield-related traits under aerobic conditions. The conglomeration of these traits at the seedling stage, panicle stage as well as reproductive stages in different environments have given key insights on the association of SSR markers with highly variable and correlated phenotypic traits and the identification of novel MTAs and regions. Consistent MTAs were identified for RM25310 (GP and TDW), RM2584 (GP), RM14753 and RM80 (RFW), RM22961 (SDW, SL, SVI, TSL), RM18472 (SFW, TFW), RM1385 (SVI-II, TSL), RM25310 (GP, SDW, SVI-II, TDW and TFW), RM80 (RFW, SFW and TFW), RM22961 (SL, SVI-I, TSL), RM1385 (SL, TSL), RM410 (GYP), RM20698 (PH), RM2584 (SF), RM5179 (TNG), RM2584 (SW and GYP) under aerobic conditions. Rice lines having better early seedling vigour, robust root traits, yield and yield-related traits can be utilized for improving early seedling vigour, root, yield and yield related traits in breeding programmes as a pre-breeding material and donor lines for the aerobic system of rice cultivation. The stable introgression lines and mutant lines identified for robust root traits, and can be proposed for further evaluation in the multi-location rice trials of All India Coordinated Research Project on Rice (AICRPR) trials. The novel marker traits associations identified in this study can be validated in mapping populations to deploy in the marker-assisted breeding programs. Identification of candidate genes in the MTA region needs to be validated using gene expression studies and can be functionally validated through over-expression or new breeding technologies like gene. The association panel used in this study can be a basis for haplotype breeding for developing climate-resilient rice lines. The development of high yield potential, seedling vigor lines suitable for the aerobic system of cultivation is a need of the hour and will be in line with the government policy to double farmer’s income and livelihood with low input costs and maximum yield.

## Data availability statement

The original contributions presented in this study are included in the article/**Supplementary Material**; further inquiries can be directed to the corresponding author.

## Author contributions

The study was conceptualized and planned by KB; phenotyping of the panel was executed by RP, H, NM, AP; phenotypic and genotypic data analysis were done by RP, MB, DB, KB, GC, AS, YR, PS, JD; supervision and critical editing were done by KB, MM, LR, DS and RS. All authors contributed to the article and approved the submitted version.
