# Supplementary material for: Genome-wide association studies in rice germplasm reveal significant genomic regions for root and yield-related traits under aerobic and irrigated conditions
Source: Front Plant Sci. 2023 Jul 18;14:1143853. doi: 10.3389/fpls.2023.1143853 (PMC10395336; doi:10.3389/fpls.2023.1143853)
Supplement: Supplementary file 1 [file DataSheet_1.zip › Supplementary Tables.docx]

**Supplementary Tables**

**Supplementary Table S1. List of plant material (rice association panel) investigated in the present study.**

| **Sl. No.** | **Rice lines** | **Biological status of accession** | **Attributes** |
| --- | --- | --- | --- |
| 1 | KJ-214 | IRGC25892 | Tropical *japonica* accessions |
| 2 | KJ-216 | IRGC25660 |  |
| 3 | KJ-219 | IRGC25214 |  |
| 4 | KJ-221 | IRGC24528 |  |
| 5 | KJ-222 | IRGC24273 |  |
| 6 | KJ-226 | IRGC23351 |  |
| 7 | WB-3 | Wangoo-Phou | North-Eastern landraces |
| 8 | WB-5 | Phouren |  |
| 9 | WB-6 | Chakhao |  |
| 10 | WB-8 | Moirangphou-Yenthik |  |
| 11 | WB-10 | Langphou |  |
| 12 | WB-12 | Langphou-Chakao |  |
| 13 | WB-14 | Ayangleima |  |
| 14 | WB-15 | Heimang-Phou |  |
| 15 | WB-16 | Phouoibi |  |
| 16 | WB-22 | Moirang-Phou-Khokngangbi |  |
| 17 | WB-23 | Kakcheng-Phou |  |
| 18 | WB-24 | Pat-Phou |  |
| 19 | WB-26 | Taothabi |  |
| 20 | WB-27 | Langmanbi |  |
| 21 | WB-29 | Akut-Phou |  |
| 22 | WB-30 | MoirangPhou-Angouba |  |
| 23 | WB-32 | Keibi-Phou |  |
| 24 | WB-39 | Phouren-Amubi |  |
| 25 | GNV-1109 | Selection from PR 35887-1-21-2-1 | Advanced breeding lines |
| 26 | GNV-1089 | GGV-05-01 X NES-07-03 |  |
| 27 | RNR-15048 | MTU1010 X JGL3855 | Popular mega variety |
| 28 | Pokkali | Cultivated variety | Cultivated variety |
| 29 | SIRI-1253 | BPT-5204 X Kavya | Cultivated variety |
| 30 | GNV-14-96-1 | BPT-5204 X Nerica line | Advanced breeding line |
| 31 | RP-Bio | Samba Mahsuri*4 X SS1113 | Cultivated variety |
| 32 | Tellahamsa | HR12 X TN1 | Popular mega variety |
| 33 | FL-478 | IR29 X Pokkali | Cultivated variety |
| 34 | Ratnamudi | - | Karnataka landrace |
| 35 | Ratnachudi | - | Karnataka landrace |
| 36 | Tanu | MandyaVijaya X CTH-3 | Popular mega variety |
| 37 | Rasi | TN1 X C029 | Popular mega variety |
| 38 | Swarna Sub-1 | Swarna*3 X IR49830-7-1-2-3 | Popular mega variety |
| 39 | MTU-1010 | Krishnaveni X IR-64 | Popular mega variety |
| 40 | BPT-5204 | GEB-24 X TN1 X Mahsuri | Popular mega variety |
| 41 | Jaya | TN1 X T141 | Popular mega variety |
| 42 | MTU-1001 | Krishnaveni X IR-64 | Popular mega variety |
| 43 | TI-3 | Ethyl methanesulfonate (EMS) mutants of BPT-5204 | Mutant of BPT-5204 |
| 44 | TI-4 |  | Mutant of BPT-5204 |
| 45 | TI-8 |  | Mutant of BPT-5204 |
| 46 | TI-11 |  | Mutant of BPT-5204 |
| 47 | TI-12 |  | Mutant of BPT-5204 |
| 48 | TI-15 |  | Mutant of BPT-5204 |
| 49 | TI-16 |  | Mutant of BPT-5204 |
| 50 | TI-17 |  | Mutant of BPT-5204 |
| 51 | TI-18 |  | Mutant of BPT-5204 |
| 52 | TI-19 |  | Mutant of BPT-5204 |
| 53 | TI-23 |  | Mutant of BPT-5204 |
| 54 | TI-24 |  | Mutant of BPT-5204 |
| 55 | TI-25 |  | Mutant of BPT-5204 |
| 56 | TI-35 |  | Mutant of BPT-5204 |
| 57 | TI-36 |  | Mutant of BPT-5204 |
| 58 | TI-37 |  | Mutant of BPT-5204 |
| 59 | TI-44 |  | Mutant of BPT-5204 |
| 60 | TI-87 |  | Mutant of BPT-5204 |
| 61 | TI-112 |  | Mutant of BPT-5204 |
| 62 | TI-128 |  | Mutant of BPT-5204 |
| 63 | TI-166 |  | Mutant of BPT-5204 |
| 64 | TI-124 |  | Mutant of BPT-5204 |
| 65 | Swarna | Drought tolerant line | Cultivated variety |
| 66 | Vandana | C 22 / Kalakeri | Cultivated variety |
| 67 | Wazuhophek | North-Eastern landrace | Landrace |
| 68 | Improved Samba Mahsuri (ISM) | Parental line | Cultivated variety |
| 69 | PUP-225 | ISM × Vandana | Near isogenic line |
| 70 | PUP-229 | MTU1010 × Vandana | Near isogenic line |
| 71 | PUP-230 | MTU1010 × Vandana | Near isogenic line |
| 72 | KR-209 | Wazuhophek × ISM | Recombinant inbreed line |
| 73 | KR-262 | Wazuhophek × ISM | Recombinant inbreed line |
| 74 | CR Dhan-202 | IRRI 148/IR 78877-208-B-1-1 | Aerobic adapted cultivar |
| 75 | SR-50 | Gomiri Bora | Short rice, Nagaon landrace, Assam |
| 76 | MAS 946-1 | Sharada | Aerobic adapted cultivar |
| 77 | PB-3 | Pusa Basmati | Pusa Basmati |
| 78 | CR Dhan-201 | IRRI 76569-259-1-2-1/ CT 6510-24-1-2 | Aerobic adapted cultivar |
| 79 | DRR Dhan-42 | **IR-64 (**Drt 1**)** | Drought tolerant variety |
| 80 | DRR Dhan-44 | IET 22081 | Aerobic adopted cultivar |
| 81 | NPS-24 | Swarna × *Oryza nivara* (IRGC81848) | Wild introgression line |
| 82 | NPS-53 |  | Wild introgression line |
| 83 | NPS-25 |  | Wild introgression line |
| 84 | DB-5 | Swarna × *Oryza nivara* (IRGC81848) | Wild introgression line |
| 85 | DB-6 |  | Wild introgression line |
| 86 | DB-7 |  | Wild introgression line |
| 87 | DB-9 |  | Wild introgression line |
| 88 | NPK-13 | Swarna × *Oryza nivara* (IRGC81832) | Wild introgression line |
| 89 | NPK-27 |  | Wild introgression line |
| 90 | NPK-40 |  | Wild introgression line |
| 91 | NPK-41 |  | Wild introgression line |
| 92 | NPK-43 |  | Wild introgression line |
| 93 | NPK-45 |  | Wild introgression line |
| 94 | SM-363 | Ethyl methanesulfonate (EMS) mutants of Nagina 22 (N22) | Mutant of N22 |
| 95 | SM-669 |  | Mutant of N22 |
| 96 | SM-686 |  | Mutant of N22 |
| 97 | PB-4 | Pusa Basmati | Pusa Basmati |
| 98 | PB-5 | Pusa Basmati | Pusa Basmati |
| 99 | Sahbhagi Dhan | IR74371-70-1-1 (IR5541-04 × WayRarem) | Drought tolerant variety |
| 100 | Sabita | IET-8970 | *Oryza sativa* |
| 101 | DRR Dhan-41 | IET 22729 (RP 5311-PR26703-3B-PJ7) | Drought tolerant variety |
| 102 | KMR-3 | IR58025A | Restorer line |
| 103 | IR-64 | IR 5657-33-2-1 | *Oryza sativa* |
| 104 | ATR-486 | Azucena × Dular | Introgression line |
| 105 | ASG-73 | Aromatic short grain Seeta Bhog | West Bengal landrace |
| 106 | ASG-126 | Aromatic short grain line | Uttar Pradesh landrace |
| 107 | CG-219 | Saali | *Oryza glaberrima* accessions |
| 108 | CG-228 | Dissi |  |
| 109 | CG-242 | Mow |  |
| 110 | CG-243 | Mouli |  |
| 111 | Basmati-370 | Basmati selection | North-Eastern landrace |
| 112 | Thurur Bhog | Uttar Pradesh landrace | Uttar Pradesh landrace |
| 113 | D-92 | North-Eastern landrace | North-Eastern landrace |
| 114 | JBB-661 | IET 27934  (RPbio226/IRGC 39050//MTU1081) (RP 5364-B-24-2-1-1-1-1) | Tropical *japonica*× *indica* |
| 115 | JBB-610 | IET 27742, Swarna/IRGC 63246//DSB3 (RP 5401-B-22-6-5-1-1-1) |  |
| 116 | JBB-684 | Swarna/IRGC 63248//Swarna Sub-1  (RP 5507-684-40-1-1-1-1) |  |
| 117 | JBB-1325 | IET 29287 (Swarna*2/ IRGC 4105)  (RP 5405-13-1-3-1-1-1-1) |  |
| 118 | JBB-631-1 | Swarna*2/ IRGC 4105 (RP 5405-JBB-631-1-1-1-1-1-1) |  |

**Supplementary Table S2. List of polymorphic markers used in the study.**

| **Sl. No.** | **Marker name** | **Chr. No.**  **_Physical position(Mb)** | **Forward sequence** | **Reverse sequence** | **Expected size (bp)** | **Reported QTL (if applicable)** | **Marker type** | **Reference (if applicable)** |
| --- | --- | --- | --- | --- | --- | --- | --- | --- |
| 1 | RM5933 | 8_27.47 | AGCGATTCAGAACGAATCAACG | TGCCAAAGCTACACAAATCTGACC | 220 |  | Random markers |  |
| 2 | RM16283 | 4_0.42 | CTCTTGGGCCCACCTATCATACC | GCATCTTGCCTGAGCATGTACG | 287 |  | Random markers |  |
| 3 | RM3524 | 4_22.87 | CTGTCTCCGTCTTCCTCACTCG | TGGAGAAATCTCCCTTCCTGAGC | 90 |  | Random markers |  |
| 4 | RM17900 | 5_2.78 | CCTCGAGGATGTTCTTCAGC | CCTCAACTTGTCATCAGTCTGC | 251 |  | Random markers |  |
| 5 | RM18182 | 5_8.8 | AGCCATGTAGCAACAGACCAGTTTCG | ACGGGCAGGCTTACTTTCTTCTGG | 278 |  | Random markers |  |
| 6 | RM18472 | 5_16.64 | AAAGTTCGGCATCCTAAACCCTAACC | CTACTGAAAGGGAGGGCGTTGC | 261 |  | Random markers |  |
| 7 | RM20023 | 6_15.62 | CTGACCTGACGGCTGACATGACC | CAAGCAACCTTTCGGGATTTGC | 242 |  | Random markers |  |
| 8 | RM18939 | 5_24.61 | CCAATATACGGGTGAAATCC | AGCTAGCTACGTGTGTGACG | 203 |  | Random markers |  |
| 9 | RM27879 | 12_9.28 | GCTGTAATTGTACGGCTCCAACC | ATAACGAGCTGATCGCAATGTGG | 332 |  | Random markers |  |
| 10 | RM26558 | 11_12.61 | GTTCAAATGAGGTCAGCCTATGG | AGAGTCAATGCATGCTCAAACC | 255 |  | Random markers |  |
| 11 | RM21749 | 7_20.49 | TAGCTTCCTACTTCCGCCTTCTATCG | ACAGTGGGAGGGAGTACATACACTGG | 393 |  | Random markers |  |
| 12 | RM16582 | 4_10.94 | AAGGCACAAAGGAACAGTGC | TTTCCATACACCCAAAGTCG | 213 |  | Random markers |  |
| 13 | RM17263 | 4_26.28 | GGCTCCCAAGTGTAGTATACGG | TCTGTAATCGACTGATGTCTCG | 169 |  | Random markers |  |
| 14 | RM20948 | 7_2.53 | GCAAGCTGGAAGAACATCGTACC | TGCTTATGGTTCTGGTCACTTCG | 395 |  | Random markers |  |
| 15 | RM21165 | 7_5.58 | AGTTGCTACTGGCAGTTTGTTTGG | CAAGGGTATGTGACCCTTATTGAGC | 317 |  | Random markers |  |
| 16 | RM22031 | 7_26.34 | GACTCACGGAACAGTATTAAGGTTGG | CACAATCCATGCACGTAAACTCG | 372 |  | Random markers |  |
| 17 | RM22763 | 8_10.34 | GGATCTTCTCCCTAAGATCCAACG | CAGGACACCAACCAAACAGACC | 258 |  | Random markers |  |
| 18 | RM22961 | 8_16.6 | CATAACACCGGCGTCAAAGTCG | TTATTTGCAAGGCCCTGTTTGC | 184 |  | Random markers |  |
| 19 | RM25310 | 10_11.78 | CACGTAATTGCCCGCGTTGC | GATACGGTGATCTGGGAGGAATCG | 444 |  | Random markers |  |
| 20 | RM28157 | 12_17.44 | GCTTAATTTCTGACAGACCAGTGC | GATCTAAACACAGCCTTCCTTGG | 349 |  | Random markers |  |
| 21 | RM14472 | 3_3.91 | TCCTGCAGTCTTTGCATATTGTCTCG | ATCACAATCCGGTCCATGACAGC | 498 |  | Random markers |  |
| 22 | RM24842 | 9_22.54 | CGTCATCTGAATTGTTGCTTACCC | TATGCACAGCCGGGTACATAATCC | 259 |  | Random markers |  |
| 23 | RM38 | 8_2.11 | ACGAGCTCTCGATCAGCCTAGC | CACTCCATGGAAGAGGCAAGC | 472 |  | Random markers |  |
| 24 | RM1201 | 1_7.16 | GCTACGTACGAGCCCTAGTTACCG | TACCGCGCCACATATACACAACC | 117 |  | Random markers |  |
| 25 | RM1385 | 2_26.67 | GACAGGTAAGGTGTGGTGGTAAGG | AAACCTTTCTCAAACGCACACG | 189 |  | Random markers |  |
| 26 | RM5179 | 2_13.98 | TCCCAAACACAATCCTTTGACC | ACACGATTAACCTTCGTGAGCTACC | 222 |  | Random markers |  |
| 27 | RM6100 | 10_18.37 | TTCCCTGCAAGATTCTAGCTACACC | TGTTCGTCGACCAAGAACTCAGG | 387 |  | Random markers |  |
| 28 | RM7097 | 3_26.68 | GGCCATTATGTGCATCTCTCAGC | GGATCGATCGACATCAATCTTGG | 173 |  | Random markers |  |
| 29 | RM10039 | 1_0.61 | TCTCTCAGGTCTCCATCACTCC | GATGAGGTAGGTAATTGGTACACG | 177 |  | Random markers |  |
| 30 | RM10149 | 1_3.04 | AGAAGCCCGTGGAGTTTGTGG | AAACGAGCACTCGCGGAAGC | 271 |  | Random markers |  |
| 31 | RM15981 | 3_31.64 | GGTTAAAGGGAGGACACCACTCG | TCTAGCCAGGCATGACAAGAACC | 164 |  | Random markers |  |
| 32 | RM242 | 9_18.64 | AAACACATGCTGCTGACACTTGC | TTACTAGATTTACCACGGCCAACG | 259 |  | Random markers |  |
| 33 | RM474 | 10_1.8 | TACACGAGGGAGTACTCGAATGG | CATGGAGGTATAGAAGAGCATTGG | 265 |  | Random markers |  |
| 34 | RM17377 | 4_29.12 | ATATTACTTCGACGCTGGATCAGG | GTCAGTTCGTCAGGCACAACG | 192 |  | Random markers |  |
| 35 | RM284 | 8_21.14 | TCTCTGATACTCCATCCATCC | CCTGTACGTTGATCCGAAGC | 169 |  | Random markers |  |
| 36 | RM25756 | 10_20.24 | CCTCTGACGCCTTGAATGAGG | CAGAAATAGCCAATGGAACTGAGG | 141 |  | Random markers |  |
| 37 | RM27499 | 12_1.93 | TTACAGAAGCAGGAGGGAAGTCG | ACAATCATCGCCGGCATAGC | 133 |  | Random markers |  |
| 38 | RM20698 | 6_29.8 | ACGGTCGTAGCAATAACTAGC | GGTCATAGGTCATAACTAGTCTGC | 636 |  | Random markers |  |
| 39 | RM3187 | 6_20.92 | TCCCCACATCGTGTCGTC | TTTTTCCCCTTCTACCCTCG | 169 |  | Random markers |  |
| 40 | RM1149 | 5_28.42 | GCGCTGATCCTGCCTAGTAC | TTCTCCTCCTCCTCCTCCTC | 142 |  | Random markers |  |
| 41 | RM12434 | 2_2.07 | TTGGTCATCTTTGGTTGGTTCAGG | GGACAATTCACACAGCAGAATTGG | 150 |  | Random markers |  |
| 42 | RM14753 | 3_9.58 | TGGAAGATGGGAGAAAGCATGG | AACGCTCTTAAGCCACCACATACC | 188 | *qSH30-2,1* | SVI | Muralidhara, et al. (2020) |
| 43 | RM467 | 10_13.04 | TGTTGTCACATGAGATGGCTATGC | GCTGACCTTGTGAGACGTTTAGACC | 648 | *qSH45-3,1, qAGR45-3,1,Qrgr45-3-1* | SVI | Muralidhara, et al. (2020) |
| 44 | RM13155 | 2_15.37 | TACCAACAGGGAGTTGTCTCTCG | AGCGACGGTGTAAAGAATAAGTCG | 299 | *qNLA45-10,qNT45-10,1, qSLA15-10,1, qLAR15-10,1* | SVI | Muralidhara, et al. (2020) |
| 45 | RM3029 | 3_6.68 | GACGAACACACAGTGACATGAGG | GCCACGACTCCTGATCATATTCC | 479 | *qSDW30-2,1* | SVI | Muralidhara, et al. (2020) |
| 46 | RM566 | 9_14.65 | AATATGGTGGCGCGTACATCC | TGATCGAGCCAACAACAACTGG | 694 | *qSH45-3,1,Qagr45-3,1* | SVI | Muralidhara, et al. (2020) |
| 47 | RM13962 | 2_31.37 | ACGACGGAAACACAATTCACTGG | GAGCCATATTTAGTCCCGGTTGG | 143 | *qLA30-9,1,qLA45-9,1* | SVI | Muralidhara, et al. (2020) |
| 48 | RM19179 | 5_29 | TCAGTACACTGTGGCGCTGTAGG | GACGGATCTAGGTTTCCGTTAAGC | 400 | *qSLA15-2,1* | SVI | Muralidhara, et al. (2020) |
| 49 | RM7083 | 6_0.45 | TGTGTTTTGGTGTGCCTGAC | ACTACCGTGGTACCAAACGG |  | *Trait linked* |  |  |
| 50 | RM6837 | 3,12_7 | ACCTGGTGCAAGAACCTGAC | CGGTAGAGGACGTCCATGTC | 155 | *qRL12-3* | Root and shoot weight | Zhao et al. (2019) |
| 51 | RM7086 | 1_6 | TATTTTGCCTCCAAGAGGCC | GGTGCATGGTTCTGAGGAAC | 160 | *qRL1.4-NERICA7* | Root length | Zhao et al. (2019) |
| 52 | RM3455 | 12_5 | TGAATCCACACTCGCAGATC | GCCAGTCCACGATTGGTC | 140 | *qRL12-3* | Root length | Zhao et al. (2019) |
| 53 | RM6107 | 2_25 | CTACTCCCTAGTTGGCAGCG | TCTCCTGCAGGTACGTGCC | 92 | *qRL1.4-NERICA7* | Root length | Zhao et al. (2019) |
| 54 | RM7009 | 2_33 | GGGATTTATTGGTCGGACTG | GTAAGGCGGCACAAAGAATC | 199 | *qRO1* | Maximum root length and quick rooting | Kitomi et al. (2019) |
| 55 | RM202 | 11_18 | CAGATTGGAGATGAAGTCCTCC | CCAGCAAGCATGTCAATGTA | 99 | *qRL1.4-NERICA7* | Root length | Zhao et al. (2019) |
| 56 | RM1015 | 12_22 | TGTATGACTTTTTAGCATTG | CCACATTCATTTAGATGTTA | 189 | *qDRL11* | Deep root length | Sabar et al. (2019) |
| 57 | RM262 | 2_25.825 | CATTCCGTCTCGGCTCAACT | CAGAGCAAGGTGGCTTGC | 145 | *qRL1.4-NERICA7* | Root length | Zhao et al. (2019) |
| 58 | RM105 | 9_8.025 | GTCGTCGACCCATCGGAGCCAC | TGGTCGAGGTGGGGATCGGGTC | 154 |  | Root length density | Dharmappa et al. (2019) |
| 59 | RM168 | 3_28 | TGCTGCTTGCCTGCTTCCTTT | GAAACGAATCAATCCACGGC | 134 | *qDRL9* | Deep root length | Sabar et al. (2019) |
| 60 | RM252 | 4_24.75 | TTCGCTGACGTGATAGGTTG | ATGACTTGATCCCGAGAACG | 116 | *qTRDW3* | Total root dry weight | Sabar et al. (2019) |
| 61 | RM410 | 9_18 | GCTCAACGTTTCGTTCCTG | GAAGATGCGTAAAGTGAACGG | 216 |  | Root thickness | Zhang et al. (2016) |
| 62 | RM520 | 3_31 | AGGAGCAAGAAAAGTTCCCC | GCCAATGTGTGACGCAATAG | 183 | *qDRL9* | Deep root length | Sabar et al. (2019) |
| 63 | RM1146 | 10_20 | ACCCCGATGATCGATTGTAC | CCCTATTCCCGTGTAAATCG | 247 | *qTRDW3* | Total root dry weight | Sabar et al. (2019) |
| 64 | RM1388 | 4_25 | TTCAATGAGGCAAAGGTAAG | ATTGTAGCTTGGACTAGGGG | 183 |  | Root weight | Zhao et al. (2019) |
| 65 | RM109 | 2_0.183 | GCCGCCGGAGAGGGAGAGAGAG | CCCCGACGGGATCTCCATCGTC | 236 |  | Root length density | Dharmappa et al. (2019) |
| 66 | RM229 | 11_18.4 | CACTCACACGAACGACTGAC | CGCAGGTTCTTGTGAAATGT | 97 |  | Root weight | Zhao et al. (2019) |
| 67 | RM106 | 2_30.725 | CGTCTTCATCATCGTCGCCCCG | GGCCCATCCCGTCGTGGATCTC | 116 | *qDRL11* | Deep root length | Sabar et al. (2019) |
| 68 | RM3117 | 3_4 | GCCATCTCTCTCTCTCTCTCTC | CCTTAGCTCATCAAGCGAGG | 297 |  | Root weight | Zhao et al. (2019) |
| 69 | RM5300 | 2_34 | CCACCCCATCATTATTGAGG | AAGCTGAGGTTGGTTGCTTG | 111 |  | Root length | Zhao et al. (2019) |
| 70 | RM5501 | 1_35 | GCGCTTCTACTTCCACAAGG | GGTTGGCGTACGTAGAGAGG | 188 |  | Root length | Zhao et al. (2019) |
| 71 | RM16 | 3_23 | CGCTAGGGCAGCATCTAAA | AACACAGCAGGTACGCGC | 135 | *qRL1.4-NERICA7* | Maximum root length | Obara et al*.* (2019) |
| 72 | RM80 | 8_24 | TTGAAGGCGCTGAAGGAG | CATCAACCTCGTCTTCACCG | 181 |  | Root volume | Dharmappa et al. (2019) |
| 73 | RM3430 | 6_27 | ACGACGACGATCAAGAAC | CGAGAGCCACCTAATCTTG | 142 |  | Root length density | Dharmappa et al. (2019) |
| 74 | RM7006 | 2_6.13 | CTCGTTTATCCTCCCAGTGC | CACTTGTATCCAGAAGCAGG | 214 | *qRO2* | Maximum root length and quick rooting | Kitomi, et al. (2019) |
| 75 | RM2584 | 8_7.56 | AAGATGAAGTTCACTCTGGA | ACTTGAGATGATCAGATTGC | 236 |  | Root length density | Dharmappa et al. (2019) |
| 76 | RM5944 | 3_8.81 | GAGCCGCATCAACCAGTTAC | CAGTACAGCGCGCACTACAC | 133 |  | Root weight | Dharmappa et al. (2019) |
| 77 | RM8111 | 1_6.27 | AGGTAACTAAGCTAGGTGTT | TAGGTACAGTAATACCAAGC | 213 |  | Root length | Zhao et al. (2019) |
| 78 | RM6849 | 3_3.28 | CGTCAACTGCATCACCACC | TCCGACTGATCATCATCGAC | 149 | *qRL3-NERICA7* | Maximum root length | Obara, et al. (2019) |
| 79 | RM7075 | 1_15.11 | TATGGACTGGAGCAAACCTC | GGCACAGCACCAATGTCTC | 124 | *qRT5-1* | Shoot length, shoot and root weight | Zhao *et al*.(2019) |
| 80 | RM6283 | 3_16.96 | TGGAGACTGAGCTGATGCC | TCAGGTGGTCGGTTCCTTAC | 155 |  | Root length | Zhao et al. (2019) |
| 81 | RM20069 | 6_16.54 | GCGAGCGAGAGGAGAGATAGACG | CGAATTCGGCACGAGTAATAGGG | 93 |  | Root length | Zhao et al. (2019) |
| 82 | RM16030 | 3_32.7 | GCGAACTATGAGCATGCCAACC | GGATTACCTGGTGTGTGCAGTGTCC | 157 | *qRDWN6xB* | Root dry weight | Anis et al. (2019) |
| 83 | RM3183 | 6_12.44 | GCTCCACAGAAAAGCAAAGC | TGCAACAGTAGCTGTAGCCG | 99 | *qDRD3.1* | Deep root diameter | Sabar, et al. (2019) |
| 84 | RM6144 | 10_15.6 | TGGAACTCAACGGGAGTCTC | GAAGTAGTGGAATCGGCGAG | 140 | *qRT12-2* | Shoot length, shoot and root weight | Zhao et al. (2019) |
| 85 | RM5 | 1_23.97 | TGCAACTTCTAGCTGCTCGA | GCATCCGATCTTGATGGG | 143 |  | Root weight | Zhao et al. (2019) |
| 86 | RM224 | 11_30.02 | ATCGATCGATCTTCACGAGG | TGCTATAAAAGGCATTCGGG | 113 | *qRT1-2* | Shoot length, shoot and root weight | Zhao et al. (2019) |
| 87 | RM3825 | 1_36.47 | AAAGCCCCCAAAAGCAGTAC | GTGAAACTCTGGGGTGTTCG | 157 |  | Root length | Zhao et al. (2019) |
| 88 | RM3217 | 4_30.11 | GTTGCAAGGTTGCAACACAG | GTGGCAGCCAAGATGGAC | 147 |  | Root volume | Dharmappa et al. (2019) |
| 89 | RM3709 | 1_31.94 | TATATTGAGGGAGCAAGCCG | CATCCCAAAGCTAGAACCCC | 194 |  | Root length | Zhao et al. (2019) |
| 90 | RM6872 | 7_4.65 | GGATGAACACTGATGATGGC | ACCTCCACCACGATATCCAC | 113 | *qRL1.4-NERICA7* | Maximum root length | Obara, et al. (2019) |
| 91 | RM4455 | 10_11.66 | CTCTCAAAGAACTAGGACTC | GAGAAGGTATGATAACCAAT | 95 |  | Root length | Zhao et al. (2019) |
| 92 | RM1112 | 4_34.23 | TCAGGACACATGGCCCTTAC | CAGCTCCTGACAGAGCACAC | 116 |  | Root volume | Dharmappa et al. (2019) |
| 93 | RM1141 | 1_1.62 | TGCATTGCAGAGAGCTCTTG | CAGGGCTTTGTAAGAGGTGC | 133 |  | Root weight | Dharmappa et al. (2019) |
| 94 | RM3188 | 2_3.45 | TCACGAGTCGTTCGTTCTTG | CTTGCTGCTCAAGTGGTGAG | 100 |  | Root length | Zhao et al. (2019) |

**Supplementary Table S3 A. Analysis of variance for seedling vigour index traits in rice association panel at 14 and 21 DAS under polyhouse condition during 2018 and 2019.**

| **Traits** | **Year** | **Days** | **Mean sum of squares** | **Error** | **CD** | **CV (%)** |
| --- | --- | --- | --- | --- | --- | --- |
|  |  |  | **Lines** |  |  |  |
| ***d*. *f.*** |  | | 117 | 118 |  |  |
| **G%** | **2018** | 14DAS | 97.83** | 8.47 | 4.69 | 2.98 |
|  |  | 21DAS | 92.95** | 5.93 | 3.92 | 2.49 |
|  | **2019** | 14DAS | 35.45ns | 30.93 | N/A | 5.68 |
|  |  | 21DAS | 85.55** | 8.75 | 4.76 | 3.02 |
| **SL** | **2018** | 14DAS | 59.54** | 2.28 | 2.43 | 1.30 |
|  |  | 21DAS | 135.55** | 3.30 | 2.93 | 7.23 |
|  | **2019** | 14DAS | 3,249.66** | 12.51 | 7.014 | 6.07 |
|  |  | 21DAS | 96.26** | 4.28 | 3.33 | 8.22 |
| **RL** | **2018** | 14DAS | 3.61** | 0.89 | 1.52 | 1.21 |
|  |  | 21DAS | 8.17** | 0.64 | 1.30 | 9.36 |
|  | **2019** | 14DAS | 15.35** | 5.01 | 4.44 | 4.93 |
|  |  | 21DAS | 9.01** | 1.78 | 2.15 | 1.84 |
| **TSL** | **2018** | 14DAS | 65.09** | 2.85 | 2.72 | 4.77 |
|  |  | 21DAS | 154.28** | 3.89 | 3.18 | 5.84 |
|  | **2019** | 14DAS | 2.87** | 1.60 | 2.51 | 1.64 |
|  |  | 21DAS | 113.53** | 6.88 | 4.23 | 7.53 |
| **RSLR** | **2018** | 14DAS | 0.04** | 0.01 | 0.13 | 2.4 |
|  |  | 21DAS | 0.043** | 0.01 | 0.08 | 13.39 |
|  | **2019** | 14DAS | 180.97** | 6.82 | 5.17 | 6.73 |
|  |  | 21DAS | 0.04** | 0.01 | 0.11 | 6.19 |
| **SFW** | **2018** | 14DAS | 5721.20** | 16.01 | 6.44 | 3.82 |
|  |  | 21DAS | 14,119.76** | 5.87 | 3.90 | 1.24 |
|  | **2019** | 14DAS | 20.90** | 10.07 | 6.29 | 1.54 |
|  |  | 21DAS | 12,837.61** | 82.21 | 14.59 | 4.63 |
| **RFW** | **2018** | 14DAS | 2961.91** | 3.55 | 3.04 | 3.34 |
|  |  | 21DAS | 5,550.38** | 4.44 | 3.39 | 2.13 |
|  | **2019** | 14DAS | 0.03** | 0.01 | 0.16 | 1.99 |
|  |  | 21DAS | 5,184.80** | 7.94 | 4.54 | 2.87 |
| **TFW** | **2018** | 14DAS | 13009.60** | 19.63 | 7.13 | 2.75 |
|  |  | 21DAS | 31,741.35** | 9.30 | 4.91 | 1.04 |
|  | **2019** | 14DAS | 8,515.81** | 355.77 | 37.40 | 3.02 |
|  |  | 21DAS | 28,470.66** | 86.37 | 14.96 | 3.16 |
| **RSFWR** | **2018** | 14DAS | 0.43** | 0.01 | 0.11 | 1.11 |
|  |  | 21DAS | 0.09** | 0.01 | 0.03 | 3.94 |
|  | **2019** | 14DAS | 2,389.64** | 606.38 | 48.82 | 4.14 |
|  |  | 21DAS | 0.09** | 0.01 | 0.06 | 6.59 |
| **SDW** | **2018** | 14DAS | 229.21** | 2.04 | 2.30 | 2.80 |
|  |  | 21DAS | 575.32** | 8.78 | 4.77 | 8.95 |
|  | **2019** | 14DAS | 1,615.28** | 342.33 | 36.68 | 3.87 |
|  |  | 21DAS | 539.23** | 8.82 | 4.78 | 4.66 |
| **RDW** | **2018** | 14DAS | 13.31** | 0.60 | 1.25 | 1.80 |
|  |  | 21DAS | 36.03** | 1.24 | 1.80 | 13.89 |
|  | **2019** | 14DAS | 9,531.79** | 752.88 | 54.4 | 3.47 |
|  |  | 21DAS | 39.07** | 2.19 | 2.38 | 1.62 |
| **TDW** | **2018** | 14DAS | 283.06** | 4.19 | 3.30 | 2.11 |
|  |  | 21DAS | 759.19** | 9.80 | 5.04 | 7.61 |
|  | **2019** | 14DAS | 6,136.31** | 1,552.50 | 78.12 | 4.47 |
|  |  | 21DAS | 735.15** | 10.47 | 5.21 | 7.58 |
| **RSDWR** | **2018** | 14DAS | 0.27** | 0.01 | 0.17 | 3.76 |
|  |  | 21DAS | 0.06** | 0.01 | 0.08 | 10.77 |
|  | **2019** | 14DAS | 0.09** | 0.01 | 0.27 | 2.52 |
|  |  | 21DAS | 0.04** | 0.04 | 0.10 | 2.64 |
| **SVI-1** | **2018** | 14DAS | 675,494.98** | 27,698.07 | 267.89 | 1.81 |
|  |  | 21DAS | 1,664,521.39** | 38,161.52 | 314.44 | 5.90 |
|  | **2019** | 14DAS | 238.16** | 13.83 | 7.37 | 4.92 |
|  |  | 21DAS | 1,294,624.83** | 67,272.07 | 417.48 | 7.59 |
| **SVI-11** | **2018** | 14DAS | 2,719,149.52** | 40,018.61 | 322 | 2.12 |
|  |  | 21DAS | 7,403,836.43** | 88,243.77 | 478.15 | 7.39 |
|  | **2019** | 14DAS | 98.10** | 19.60 | 8.78 | 2.63 |
|  |  | 21DAS | 7,088,521.39** | 102,652.49 | 515.71 | 7.68 |
| **RSA** | **2018** | 14DAS | 44.03ns | 40.03 | N/A | 2.43 |
|  |  | 21DAS | 14.23** | 0.76 | 1.41 | 1.15 |
|  | **2019** | 14DAS | 3.36** | 0.95 | 1.93 | 2.62 |
|  |  | 21DAS | 15.60** | 1.09 | 1.69 | 2.23 |
| **RAD** | **2018** | 14DAS | 6.53** | 0.10 | 0.52 | 3.85 |
|  |  | 21DAS | 11.31** | 0.68 | 1.33 | 2.13 |
|  | **2019** | 14DAS | 237.34** | 17 | 8.17 | 2.79 |
|  |  | 21DAS | 4.11** | 0.15 | 0.64 | 2.60 |
| **RLPV** | **2018** | 14DAS | 459.18 | 15.81 | 6.40 | 1.43 |
|  |  | 21DAS | 299.12** | 92.67 | 15.50 | 4.09 |
|  | **2019** | 14DAS | 116.14** | 24.14 | 9.74 | 2.14 |
|  |  | 21DAS | 321.81** | 6.47 | 4.10 | 8.44 |
| **RV** | **2018** | 14DAS | 0.19ns | 0.18 | N/A | 1.60 |
|  |  | 21DAS | 34.85** | 0.08 | 0.46 | 4.09 |
|  | **2019** | 14DAS | 0.03** | 0.01 | 0.22 | 4.02 |
|  |  | 21DAS | 1.04** | 0.08 | 0.48 | 1.87 |

Significance levels: * and ** indicate the mean sum of squares are significant at p<0.05 and p<0.01 respectively, N/A: Not answered, ns: Non-significant, *d. f*.: degrees of freedom

G%: Germination (%); SL: Shoot length (cm); RL: Root length (cm); TSL: Total seedling length (cm); RSLR: Root to shoot length ratio; SFW: Shoot fresh weight (mg); RFW: Root fresh weight (mg); TFW: Total fresh weight (mg); RSFWR: Root to shoot fresh weight ratio; SDW: Shoot dry weight (mg); RDW: Root dry weight (mg); TDW: Total dry weight (mg); RSDWR: Root to shoot dry weight ratio; SVI-I: Seedling vigour index-I and SVI-II: Seedling vigour index-II; RSA: Root surface area (cm^2)^; RAD: Root average diameter (mm); RLPV: Root length per volume (cm/m^3^); RV: Root volume (cm^3^)

**Supplementary Table S3 B. Estimation of genetic variability parameters for seedling vigour index traits in rice association panel at 14 and 21 DAS under polyhouse condition during 2018 and 2019.**

| **Traits** |  | **Days** | **Min.** | **Max.** | **Mean** | **Genetic variability** | | ***h^2^* (bs)** (%) | **GAM** |
| --- | --- | --- | --- | --- | --- | --- | --- | --- | --- |
|  |  |  |  |  |  | **GCV** (%) | **PCV** (%) |  |  |
| **G%** | **2018** | 14DAS | 80.00 | 100.00 | 97.86 | 5.53 | 6.21 | 79.24 | 10.13 |
|  |  | 21DAS | 90.00 | 100.00 | 99.12 | 5.26 | 6.14 | 73.30 | 9.28 |
|  | **2019** | 14DAS | 90.00 | 100.00 | 98.52 | 4.76 | 5.94 | 64.21 | 7.86 |
|  |  | 21DAS | 80.00 | 100.00 | 97.86 | 5.18 | 6.00 | 74.47 | 9.20 |
| **SL** | **2018** | 14DAS | 9.23 | 30.30 | 16.32 | 26.88 | 28.43 | 89.4 | 52.35 |
|  |  | 21DAS | 11.50 | 45.30 | 25.22 | 26.39 | 27.34 | 93.17 | 52.48 |
|  | **2019** | 14DAS | 9.33 | 30.37 | 16.17 | 23.74 | 26.12 | 82.59 | 44.44 |
|  |  | 21DAS | 10.20 | 39.62 | 25.20 | 22.04 | 23.32 | 89.32 | 42.91 |
| **RL** | **2018** | 14DAS | 2.80 | 8.69 | 5.49 | 17.34 | 24.41 | 50.46 | 25.37 |
|  |  | 21DAS | 5.86 | 13.70 | 8.62 | 18.40 | 20.65 | 79.46 | 33.80 |
|  | **2019** | 14DAS | 3.02 | 8.91 | 6.56 | 14.44 | 23.50 | 45.36 | 18.29 |
|  |  | 21DAS | 4.48 | 14.73 | 9.65 | 16.21 | 20.98 | 59.71 | 25.81 |
| **TSL** | **2018** | 14DAS | 13.60 | 38.00 | 21.82 | 20.95 | 22.33 | 88.02 | 40.48 |
|  |  | 21DAS | 19.30 | 53.80 | 33.82 | 20.97 | 21.75 | 92.99 | 41.67 |
|  | **2019** | 14DAS | 15.58 | 36.21 | 22.73 | 16.39 | 20.48 | 64.01 | 27.01 |
|  |  | 21DAS | 18.88 | 51.42 | 34.87 | 17.21 | 18.49 | 86.63 | 32.99 |
| **RSLR** | **2018** | 14DAS | 0.11 | 0.84 | 0.36 | 30.49 | 37.85 | 64.89 | 50.60 |
|  |  | 21DAS | 0.18 | 0.87 | 0.36 | 31.82 | 34.54 | 84.85 | 60.38 |
|  | **2019** | 14DAS | 0.18 | 0.77 | 0.43 | 27.98 | 30.74 | 82.81 | 52.45 |
|  |  | 21DAS | 0.18 | 0.85 | 0.40 | 28.40 | 32.72 | 75.32 | 50.77 |
| **SFW** | **2018** | 14DAS | 11.00 | 263.00 | 105.30 | 41.63 | 41.80 | 99.17 | 85.40 |
|  |  | 21DAS | 39.40 | 374.00 | 195.60 | 35.10 | 35.12 | 99.88 | 72.26 |
|  | **2019** | 14DAS | 15.94 | 241.94 | 107.47 | 38.57 | 38.66 | 99.58 | 79.30 |
|  |  | 21DAS | 42.26 | 376.9 | 195.99 | 33.31 | 33.62 | 98.17 | 67.99 |
| **RFW** | **2018** | 14DAS | 10.50 | 113.00 | 56.51 | 55.66 | 55.76 | 99.65 | 89.46 |
|  |  | 21DAS | 19.90 | 187.00 | 99.17 | 43.39 | 43.44 | 99.76 | 89.27 |
|  | **2019** | 14DAS | 11.60 | 112.29 | 56.84 | 52.76 | 52.90 | 99.49 | 56.41 |
|  |  | 21DAS | 18.51 | 183.34 | 98.15 | 42.34 | 42.44 | 99.54 | 87.03 |
| **TFW** | **2018** | 14DAS | 24.90 | 351.00 | 161.60 | 40.83 | 40.92 | 99.56 | 83.92 |
|  |  | 21DAS | 68.70 | 536.00 | 294.60 | 34.92 | 34.94 | 99.91 | 71.91 |
|  | **2019** | 14DAS | 27.54 | 337.37 | 164.17 | 38.17 | 38.23 | 99.67 | 78.49 |
|  |  | 21DAS | 75.78 | 514.34 | 293.88 | 33.10 | 33.25 | 99.13 | 67.89 |
| **RSFWR** | **2018** | 14DAS | 0.12 | 3.32 | 0.60 | 64.85 | 65.79 | 97.16 | 73.69 |
|  |  | 21DAS | 0.18 | 1.29 | 0.52 | 34.84 | 35.06 | 98.74 | 71.31 |
|  | **2019** | 14DAS | 0.14 | 2.00 | 0.56 | 49.77 | 50.10 | 98.70 | 46.86 |
|  |  | 21DAS | 0.17 | 1.17 | 0.51 | 34.62 | 35.22 | 96.61 | 70.09 |
| **SDW** | **2018** | 14DAS | 2.35 | 40.90 | 18.41 | 47.39 | 48.04 | 97.34 | 96.33 |
|  |  | 21DAS | 4.93 | 79.60 | 33.25 | 41.52 | 42.46 | 95.63 | 83.64 |
|  | **2019** | 14DAS | 3.45 | 40.73 | 18.45 | 40.62 | 41.65 | 95.08 | 81.59 |
|  |  | 21DAS | 06.20 | 78.39 | 34.44 | 38.78 | 39.66 | 95.63 | 78.12 |
| **RDW** | **2018** | 14DAS | 1.24 | 9.57 | 4.14 | 49.91 | 53.35 | 87.51 | 96.17 |
|  |  | 21DAS | 2.16 | 19.20 | 8.07 | 42.40 | 44.63 | 90.26 | 82.98 |
|  | **2019** | 14DAS | 1.33 | 11.32 | 4.00 | 38.50 | 44.15 | 76.07 | 69.18 |
|  |  | 21DAS | 2.50 | 21.92 | 8.47 | 41.69 | 45.28 | 84.79 | 79.08 |
| **TDW** | **2018** | 14DAS | 4.48 | 48.20 | 22.55 | 42.88 | 43.84 | 95.65 | 86.38 |
|  |  | 21DAS | 8.15 | 90.00 | 41.26 | 38.43 | 39.16 | 96.29 | 77.68 |
|  | **2019** | 14DAS | 5.71 | 47.72 | 22.43 | 35.70 | 36.78 | 94.23 | 71.40 |
|  |  | 21DAS | 10.42 | 88.08 | 42.82 | 36.40 | 37.12 | 96.16 | 73.54 |
| **RSDWR** | **2018** | 14DAS | 0.06 | 2.38 | 0.30 | 28.36 | 31.42 | 88.32 | 61.48 |
|  |  | 21DAS | 0.09 | 0.88 | 0.27 | 50.65 | 54.04 | 87.84 | 97.78 |
|  | **2019** | 14DAS | 0.07 | 0.98 | 0.25 | 58.40 | 65.34 | 79.88 | 53.52 |
|  |  | 21DAS | 0.10 | 0.72 | 0.27 | 42.8 | 48.39 | 78.25 | 77.99 |
| **SVI-I** | **2018** | 14DAS | 1091.00 | 3800.00 | 2135.00 | 21.82 | 23.16 | 88.75 | 42.35 |
|  |  | 21DAS | 1545.00 | 5379.00 | 3309.00 | 22.25 | 23.00 | 93.59 | 44.34 |
|  | **2019** | 14DAS | 1517.33 | 3500.3 | 2222.83 | 16.96 | 20.96 | 65.49 | 28.28 |
|  |  | 21DAS | 1888.00 | 5142.00 | 3420.16 | 18.81 | 20.01 | 88.38 | 36.42 |
| **SVI-II** | **2018** | 14DAS | 359.00 | 4818.00 | 2207.00 | 43.06 | 44.02 | 95.68 | 86.76 |
|  |  | 21DAS | 652.00 | 8999.00 | 4038.00 | 38.87 | 39.55 | 96.57 | 78.68 |
|  | **2019** | 14DAS | 571.06.00 | 4772.00 | 2194.16 | 36.19 | 37.25 | 94.36 | 72.41 |
|  |  | 21DAS | 1042.67 | 8808.67 | 4185.48 | 36.61 | 37.35 | 96.09 | 73.93 |
| **RSA** | **2018** | 14DAS | 0.41 | 33.20 | 3.02 | 40.83 | 41.23 | 92.15 | 14.91 |
|  |  | 21DAS | 0.53 | 13.70 | 4.61 | 46.31 | 50.14 | 85.30 | 88.10 |
|  | **2019** | 14DAS | 0.25 | 7.15 | 2.31 | 51.27 | 58.24 | 77.52 | 93.00 |
|  |  | 21DAS | 1.12 | 13.31 | 4.37 | 50.85 | 56.37 | 81.37 | 94.49 |
| **RAD** | **2018** | 14DAS | 0.03 | 8.13 | 0.88 | 31.69 | 33.15 | 89.12 | 52.31 |
|  |  | 21DAS | 0.23 | 8.33 | 2.00 | 49.72 | 51.24 | 84.06 | 40.07 |
|  | **2019** | 14DAS | 0.08 | 2.55 | 0.48 | 56.72 | 59.76 | 82.26 | 41.25 |
|  |  | 21DAS | 0.20 | 6.30 | 1.37 | 85.50 | 90.43 | 89.38 | 25.51 |
| **RLPV** | **2018** | 14DAS | 8.18 | 70.20 | 32.10 | 38.02 | 39.98 | 90.44 | 74.48 |
|  |  | 21DAS | 14.40 | 84.40 | 40.11 | 30.62 | 31.85 | 92.39 | 60.62 |
|  | **2019** | 14DAS | 5.13 | 44.27 | 23.95 | 37.04 | 38.76 | 91.35 | 72.93 |
|  |  | 21DAS | 8.46 | 60.14 | 30.21 | 34.01 | 35.04 | 94.22 | 68.01 |
| **RV** | **2018** | 14DAS | 0.01 | 2.66 | 0.09 | 33.26 | 34.51 | 93.46 | 24.81 |
|  |  | 21DAS | 0.01 | 3.04 | 0.38 | 34.11 | 39.75 | 78.50 | 25.25 |
|  | **2019** | 14DAS | 0.008 | 4.17 | 0.17 | 54.66 | 56.23 | 88.39 | 49.87 |
|  |  | 21DAS | 0.03 | 3.71 | 0.43 | 37.19 | 43.99 | 78.34 | 25.14 |

PCV and GCV: Phenotypic and genotypic coefficient of variation**;** *h^2^*(bs): Heritability (broad sense); GAM: Genetic advance as *per cent* of mean

G%: Germination (%); SL: Shoot length (cm); RL: Root length (cm); TSL: Total seedling length (cm); RSLR: Root to shoot length ratio; SFW: Shoot fresh weight (mg); RFW: Root fresh weight (mg); TFW: Total fresh weight (mg); RSFWR: Root to shoot fresh weight ratio; SDW: Shoot dry weight (mg); RDW: Root dry weight (mg); TDW: Total dry weight (mg); RSDWR: Root to shoot dry weight ratio; SVI-I: Seedling vigour index-I and SVI-II: Seedling vigour index-II; RSA: Root surface area (cm^2^); RAD: Root average diameter (mm); RLPV: Root length per volume (cm/m^3^); RV: Root volume (cm^3^)

**Supplementary Table S3 C. Analysis of variance for root traits in rice association panel at panicle initiation stage under polyhouse condition during 2019.**

| **Traits** | **Mean sum of squares** | **Error** | **CD** | **CV (%)** |
| --- | --- | --- | --- | --- |
|  | **Lines** |  |  |  |
| ***d*. *f*.** | 117 | 118 |  |  |
| **SL** | 615.35** | 4.14 | 4.03 | 2.92 |
| **RL** | 248.66** | 8.99 | 5.94 | 6.40 |
| **TPL** | 1,033.59** | 12.29 | 6.95 | 3.01 |
| **RSLR** | 0.09** | 0.02 | 0.09 | 7.07 |
| **TN** | 19.68** | 6.10 | 4.90 | 23.91 |
| **SPAD** | 27.66** | 7.21 | 5.32 | 6.55 |
| **SFW** | 270.42** | 142.86 | 23.70 | 26.38 |
| **RFW** | 116.02** | 75.16 | 17.19 | 33.27 |
| **TFW** | 602.15** | 384.65 | 38.88 | 27.50 |
| **RSFWR** | 9.68NS | 9.77 | N/A | 396.61 |
| **SDW** | 73.99** | 45.88 | 13.43 | 35.30 |
| **RDW** | 26.53ns | 21.17 | N/A | 56.37 |
| **TDW** | 161.29** | 103.80 | 20.20 | 37.32 |
| **RSDWR** | 1.42ns | 1.39 | N/A | 235.40 |
| **RAD** | 9.17ns | 13.03 | N/A | 77.44 |
| **RLPV** | 64,799.40ns | 68,078.78 | N/A | 60.99 |
| **RV** | 1,069.01ns | 813.44 | N/A | 64.90 |

Significance levels: * and ** indicate the mean sum of squares are significant at p<0.05 and p<0.01 respectively, N/A: Not answered, ns: Non-significant, *d*. *f*.: degrees of freedom

SL: Shoot length (cm); RL: Root length (cm); TPL: Total Plant length (cm); RSLR: Root to shoot length ratio; TN: Tiller number; SPAD: Soil Plant Analysis Development; SFW: Shoot fresh weight (g); RFW: Root fresh weight (g); TFW: Total fresh weight (g); RSFWR: Root to shoot fresh weight ratio; SDW: Shoot dry weight (g); RDW: Root dry weight (g); TDW: Total dry weight (g); RSDWR: Root to shoot dry weight ratio; RAD: Root average diameter (mm); RLPV: Root length per volume (cm/m^3^); RV: Root volume(cm^3^)

**Supplementary Table S3 D. Estimation of genetic variability parameters for root traits in rice association panel at panicle initiation stage under polyhouse condition during 2019.**

| **Traits** | **Min.** | **Max.** | **Mean** | **Genetic variability** | | ***h^2^*(bs)** (%) | **GAM** |
| --- | --- | --- | --- | --- | --- | --- | --- |
|  |  |  |  | **GCV** (%) | **PCV** (%) |  |  |
| **SL** | 39.22 | 117.00 | 69.73 | 25.12 | 25.28 | 98.67 | 51.40 |
| **RL** | 21.50 | 73.60 | 46.83 | 23.39 | 24.23 | 93.19 | 46.52 |
| **TPL** | 67.40 | 173.50 | 116.48 | 19.41 | 19.63 | 97.75 | 39.54 |
| **RSLR** | 0.24 | 1.59 | 0.70 | 30.48 | 31.28 | 94.97 | 61.20 |
| **TN** | 3.50 | 17.00 | 10.33 | 25.16 | 34.78 | 52.35 | 37.51 |
| **SPAD** | 31.00 | 51.55 | 41.02 | 7.79 | 10.17 | 58.67 | 12.30 |
| **SFW** | 13.48 | 84.05 | 45.45 | 30.46 | 31.10 | 95.93 | 61.47 |
| **RFW** | 7.02 | 59.36 | 26.26 | 36.56 | 37.67 | 94.20 | 73.10 |
| **TFW** | 21.31 | 143.42 | 71.73 | 30.36 | 30.85 | 96.89 | 61.57 |
| **RSFWR** | 0.27 | 1.18 | 0.59 | 27.33 | 29.88 | 83.67 | 51.51 |
| **SDW** | 3.98 | 42.80 | 19.31 | 38.19 | 39.82 | 91.97 | 75.45 |
| **RDW** | 1.73 | 23.62 | 8.19 | 57.51 | 60.16 | 91.36 | 32.24 |
| **TDW** | 6.08 | 58.34 | 27.44 | 40.52 | 41.81 | 93.91 | 80.90 |
| **RSDWR** | 0.09 | 1.00 | 0.42 | 37.22 | 43.00 | 74.92 | 66.37 |
| **RAD** | 1.05 | 15.40 | 4.72 | 66.23 | 71.45 | 85.92 | 26.47 |
| **RLPV** | 109.61 | 931.49 | 430.09 | 56.47 | 60.02 | 88.52 | 21.46 |
| **RV** | 3.36 | 133.49 | 43.66 | 66.44 | 67.35 | 97.32 | 35.02 |

PCV and GCV: Phenotypic and genotypic coefficient of variation**;** *h^2^*(bs): Heritability (broad sense); GAM: Genetic advance as *per cent* of mean

SL: Shoot length (cm); RL: Root length (cm); TPL: Total Plant length (cm); RSLR: Root to shoot length ratio; TN: Tiller number; SPAD: Soil Plant Analysis Development; SFW: Shoot fresh weight (g); RFW: Root fresh weight (g); TFW: Total fresh weight (g); RSFWR: Root to shoot fresh weight ratio; SDW: Shoot dry weight (g); RDW: Root dry weight (g); TDW: Total dry weight (g); RSDWR: Root to shoot dry weight ratio; RAD: Root average diameter (mm); RLPV: Root length per volume (cm/m^3^); RV: Root volume (cm^3^)

**Supplementary Table S3 E. Analysis of variance for yield and yield-related traits in rice association panel under irrigated condition at ICAR-IIRR during *wet season* 2019.**

| **Source** | ***d. f.*** | **PH** | **PL** | **NTP** | **NPP** | **TW** | **TNG** | **SF** | **GYP** |
| --- | --- | --- | --- | --- | --- | --- | --- | --- | --- |
| **Treatment** | 117 | 301.45 ** | 5.14 ** | 4.69 ** | 3.3 ns | 15.46 ** | 869.38 ** | 19.67 ** | 15.68 ** |
| **Check** | 3 | 26.03 * | 1.14 ns | 5.56 ** | 5.4 ns | 39.98 ** | 1415.79 ** | 12.27 ** | 11.96 ** |
| **Test vs. Check** | 1 | 3470.72 ** | 8.16 * | 10.9 ** | 3.29 ns | 33.35 ** | 3815.17 ** | 85.93 ** | 85.52 ** |
| **Test lines** | 113 | 280.71 ** | 5.22 ** | 4.62 ** | 3.24 ns | 14.65 ** | 828.8 ** | 19.28 ** | 15.16 ** |
| **Block** | 5 | 4.77 ns | 2.08 ns | 11.59 ** | 7.28 * | 3.27 * | 45.5 ns | 15.32 ** | 10.88 ** |
| **Residuals** | 15 | 6.96 | 1.48 | 1.01 | 1.77 | 0.95 | 16.68 | 1.78 | 1.13 |

Significance levels: * and ** indicate the mean sum of squares are significant at p<0.05 and p<0.01 respectively, ns: Non-significant, *d*. *f*. : degrees of freedom

PH- Plant height(cm); PL- Panicle length(cm); NTP- Number of tillers per plant; NPP-Number of panicles per plant; TNG- per plant; SF- Per cent spikelet fertility; TW-Test weight (g); GYP- Grain yield per plant (g)

**Supplementary Table S3 F. Estimation of genetic variability parameters for yield and yield-related traits in rice association panel at ICAR-IIRR under irrigated condition during *wet season* 2019.**

| **Trait** | **Range** | |  | **Genetic variability** | | ***h^2^*(bs)** (%) | **GAM** |
| --- | --- | --- | --- | --- | --- | --- | --- |
|  | **Min** | **Max** | **Mean** | **GCV** | **PCV** |  |  |
| **PH** | 64.64 | 142.44 | 96.05 | 17.23 | 17.44 | 97.52 | 35.09 |
| **PL** | 15.03 | 26.43 | 20.74 | 9.32 | 11.02 | 71.62 | 16.28 |
| **NTP** | 2.39 | 17.57 | 10.77 | 17.64 | 19.95 | 78.2 | 32.18 |
| **NPP** | 1.95 | 14.05 | 9.35 | 12.98 | 19.25 | 45.5 | 18.07 |
| **TW** | 10.57 | 29.12 | 18.45 | 20.06 | 20.74 | 93.53 | 40.02 |
| **TNG** | 75.37 | 198.19 | 130.49 | 21.84 | 22.06 | 97.99 | 44.6 |
| **SF** | 70.78 | 96.16 | 87.69 | 4.77 | 5.01 | 90.77 | 9.38 |
| **GYP** | 4.26 | 26.6 | 14.89 | 25.16 | 26.16 | 92.52 | 49.93 |

PCV and GCV: Phenotypic and genotypic coefficient of variation**;** *h^2^*(bs): Heritability (broad sense); GAM: Genetic advance as *per cent* of mean

PH- Plant height (cm); PL- Panicle length (cm); NTP- Number of tillers per plant; NPP- Number of panicles per plant; TNG-Total number of grains per panicle; SF- Per cent spikelet fertility; TW-Test weight (g); GYP- Grain yield per plant (g)

**Supplementary Table S3 G. Analysis of variance for yield and yield-related traits in rice association panel under irrigated condition at ICAR-IIRR during *wet season* and *dry season* 2020.**

| **Source** | **Year** | ***d*. *f*.** | **PH** | **PL** | **NTP** | **NPP** | **TNG** | **SF** | **TW** | **GYP** |
| --- | --- | --- | --- | --- | --- | --- | --- | --- | --- | --- |
| **Treatment** | *wet season* | 117 | 340.33 ** | 5.89 ** | 3.69 ns | 2.73 ns | 905.85 ** | 19.34 ** | 18.59 ** | 29.38 ** |
|  | *dry season* |  | 320.12 ** | 5.75 ** | 3.22 ns | 2.13 ns | 821.69 ** | 19.57 ** | 16.77 ** | 22.91 ** |
| **Check** | *wet season* | 3 | 635.35 ** | 0.58 ns | 14.18 * | 8.93 * | 1830.88 ** | 100.09 ** | 60.84 ** | 34.4 ** |
|  | *dry season* |  | 582.77 ** | 0.18 * | 16.08 * | 9.80 * | 1944.85 ** | 137.26 ** | 59.34 ** | 21.46 * |
| **Test vs. Check** | *wet season* | 1 | 4017.48 ** | 31.03 ** | 42.00 ** | 37.63 ** | 7216.64 ** | 4.84 ns | 119.79 ** | 438.68 ** |
|  | *dry season* |  | 2421.83 ** | 1.19 ns | 36.35 ** | 33.61 ** | 6033.81 ** | 0.03 ns | 88.04 ** | 210.66 ** |
| **Test lines** | *wet season* | 113 | 299.95 ** | 5.81 ** | 3.07 ns | 2.26 ns | 825.45 ** | 17.32 ** | 16.58 ** | 25.63 ** |
|  | *dry season* |  | 294.55 ** | 5.94 ** | 2.58 ns | 1.65 * | 745.74 ** | 16.62 ** | 15.01 ** | 21.29 ** |
| **Block** | *wet season* | 5 | 8.51 ns | 3.33 ns | 4.20 ns | 1.71 ns | 11.47 ns | 4.15 ns | 0.49 ns | 0.86 ns |
|  | *dry season* |  | 4.60 ns | 3.30 * | 3.05 ns | 1.56 ns | 15.53 ns | 6.48 * | 0.55 ns | 6.20 ns |
| **Residuals** | *wet season* | 15 | 7.16 | 1.38 | 3.69 | 2.57 | 7.79 | 2.26 | 0.83 | 1.58 |
|  | *dry season* |  | 9.76 | 1.03 | 3.55 | 2.19 | 15.51 | 2.10 | 1.07 | 4.29 |

Significance levels: * and ** indicate the mean sum of squares are significant at p<0.05 and p<0.01 respectively, ns: Non-significant, *d*. *f*. : degrees of freedom

PH- Plant height(cm); PL- Panicle length(cm); NTP- Number of tillers per plant; NPP-Number of panicles per plant; TNG-Total number of grains per panicle; SF- Per cent spikelet fertility; TW-Test weight (g); GYP- Grain yield per plant (g)

**Supplementary Table S3 H. Estimation of genetic variability parameters for yield and yield-related traits in rice association panel at ICAR-IIRR under irrigated condition during the *wet season* and *dry season* 2020.**

| **Traits** | **Year**  **Season** | **Range** | |  | **Genetic variability** | | ***h^2^*(bs)** (%) | **GAM** |
| --- | --- | --- | --- | --- | --- | --- | --- | --- |
|  |  | **Min** | **Max** | **Mean** | **GCV** (%) | **PCV** (%) |  |  |
| **PH** | *wet season* | 62.55 | 146.94 | 100.13 | 17.09 | 17.30 | 97.61 | 34.83 |
|  | *dry season* | 61.07 | 145.82 | 97.65 | 17.28 | 17.58 | 96.69 | 35.06 |
| **PL** | *wet season* | 16.00 | 26.93 | 21.29 | 9.89 | 11.32 | 76.29 | 17.82 |
|  | *dry season* | 13.06 | 26.53 | 20.30 | 10.91 | 12.01 | 82.58 | 20.46 |
| **NTP** | *wet season* | 5.57 | 16.56 | 10.91 | 10.21 | 16.06 | 61.96 | 12.75 |
|  | *dry season* | 5.76 | 16.10 | 10.54 | 12.48 | 15.24 | 78.12 | 22.63 |
| **NPP** | *wet season* | 5.17 | 15.58 | 9.42 | 13.69 | 15.95 | 69.55 | 19.46 |
|  | *dry season* | 5.00 | 12.67 | 9.21 | 13.20 | 13.93 | 91.07 | 20.18 |
| **TNG** | *wet season* | 80.76 | 211.20 | 134.00 | 21.34 | 21.44 | 99.06 | 43.82 |
|  | *dry season* | 76.11 | 195.54 | 132.13 | 20.45 | 20.67 | 97.92 | 41.75 |
| **SF** | *wet season* | 74.76 | 96.22 | 89.07 | 4.36 | 4.67 | 86.98 | 8.38 |
|  | *dry season* | 77.89 | 95.49 | 87.75 | 4.34 | 4.65 | 87.37 | 8.37 |
| **TW** | *wet season* | 10.63 | 26.53 | 18.59 | 21.34 | 21.90 | 94.97 | 42.91 |
|  | *dry season* | 10.58 | 27.66 | 18.24 | 20.47 | 21.24 | 92.84 | 40.69 |
| **GYP** | *wet season* | 8.14 | 25.93 | 17.80 | 27.54 | 28.43 | 93.82 | 55.03 |
|  | *dry season* | 6.60 | 23.60 | 16.67 | 24.73 | 27.68 | 79.83 | 45.59 |

PCV and GCV: Phenotypic and genotypic coefficient of variation**;** *h^2^*(bs): Heritability (broad sense); GAM: Genetic advance as *per cent* of mean

PH- Plant height (cm); PL- Panicle length (cm); NTP- Number of tillers per plant; NPP- Number of panicles per plant; TNG-Total number of grains per panicle; SF- Per cent spikelet fertility; TW-Test weight (g); GYP- Grain yield per plant (g)

**Supplementary Table S3 I. Analysis of variance for yield and yield-related traits in rice association panel under irrigated condition at Dhadesugur during *wet season* 2020.**

| **Source of variation** | ***d*. *f*.** | **PH** | **PL** | **NTP** | **NPP** | **TNG** | **SF** | **TW** | **GYP** |
| --- | --- | --- | --- | --- | --- | --- | --- | --- | --- |
| **Treatment** | 117 | 193.52 ** | 3.75 ** | 4.61 ns | 3.23 ns | 404.18 ** | 20.34** | 16.83 ** | 12.3 ** |
| **Check** | 3 | 452.74 ** | 0.53 ns | 15.82 * | 13.8 0** | 1917.36 ** | 137.59** | 64.82 ** | 33.88 ** |
| **Test vs. Check** | 1 | 2815.22 ** | 58.55 ** | 35.93 ** | 16.42 * | 2611.36 ** | 6.13* | 188.26 ** | 9.39 * |
| **Test lines** | 113 | 163.44 ** | 3.35 ** | 4.04 ns | 2.83 ns | 344.48 ** | 18.19* | 14.04 ** | 11.75 ** |
| **Block** | 5 | 5.73 * | 5.34 ** | 8.80 * | 6.23 * | 16.14 ** | 9.82* | 3.90 ns | 0.58 ns |
| **Residuals** | 15 | 20.94 | 1.06 | 3.47 | 2.51 | 11.53 | 3.18 | 0.99 | 1.60 |

Significance levels: * and ** indicate the mean sum of squares are significant at p<0.05 and p<0.01 respectively, ns: Non-significant, *d*. *f*.: degrees of freedom

PH- Plant height (cm), PL- Panicle length (cm); NTP- Number of tillers per plant; NPP- Number of panicle per plant; TNG-total number of grains per panicle; SF-Per cent spikelet fertility; TW-Test weight (g); SW-Straw weight (g); GYP- Grain yield per plant (g)

**Supplementary Table S3 J. Estimation of genetic variability parameters for yield and yield-related traits in rice association panel under irrigated condition at Dhadesugur during *wet season* 2020.**

| **Trait** | **Range** | | **Mean** | **Genetic variability** | |  | |
| --- | --- | --- | --- | --- | --- | --- | --- |
|  | **Min** | **Max** |  | **GCV** | **PCV** | ***h^2^*(bs)** (%) | **GAM** |
| **PH** | 69.07 | 126.54 | 96.70 | 12.35 | 13.22 | 87.19 | 23.78 |
| **PL** | 16.15 | 27.82 | 21.99 | 6.87 | 8.32 | 68.26 | 11.72 |
| **NTP** | 5.05 | 19.13 | 10.16 | 28.36 | 29.47 | 93.14 | 24.62 |
| **NPP** | 4.59 | 15.91 | 9.20 | 31.47 | 33.15 | 91.42 | 29.21 |
| **TNG** | 83.44 | 201.87 | 127.66 | 14.29 | 14.54 | 96.65 | 28.99 |
| **SF** | 80.17 | 94.12 | 88.17 | 12.31 | 13.89 | 86.13 | 31.34 |
| **TW** | 12.25 | 26.17 | 19.65 | 18.38 | 19.07 | 92.93 | 36.56 |
| **GYP** | 7.29 | 26.01 | 13.68 | 23.29 | 25.06 | 86.36 | 44.65 |

PCV and GCV: Phenotypic and genotypic coefficient of variation**;** *h^2^*(bs): Heritability (broad sense); GAM: Genetic advance as *per cent* of mean

PH- Plant height (cm), PL- Panicle length (cm); NTP- Number of tillers per plant; NPP- Number of panicle per plant; TNG-total number of grains per panicle; SF-Per cent spikelet fertility; TW-Test weight (g); SW-Straw weight (g); HI- Harvest index (%); GYP- Grain yield per plant (g)

**Supplementary Table S3 K. Analysis of variance for yield and yield-related traits in rice association panel under the aerobic condition at Dhadesugur during *wet season* 2020.**

| **Source of variation** | ***d*. *f*.** | **PH** | **PL** | **NTP** | **NPP** | **TNG** | **SF** | **TW** | **SW** | **HI** | **GYP** |
| --- | --- | --- | --- | --- | --- | --- | --- | --- | --- | --- | --- |
| **Treatment** | 116 | 294.32 ** | 9.06 ** | 10.34 ** | 5.44 ** | 1085.39 ** | 161.40 ** | 19.33 ** | 98.27 ** | 73.00 ** | 15.15 ** |
| **Check** | 7 | 159.06 ** | 4.54 ns | 4.28 * | 2.29 ns | 995.32 ** | 211.05 ** | 3.12 ns | 102.42 ** | 62.93 ** | 2.60 * |
| **Test vs. Check** | 1 | 456.82 ** | 0.02 * | 70.65 ** | 80.49 ** | 1224.7 0** | 907.94 ** | 340.56 ** | 2.17 ns | 877.27 ** | 420.31 ** |
| **Test lines** | 108 | 301.59 ** | 9.44 ** | 10.18 ** | 4.95 ** | 1089.94 ** | 151.27 ** | 17.41 ** | 98.89 ** | 66.20 ** | 12.21 ** |
| **Block** | 4 | 15.55 ** | 0.59 * | 6.58 * | 2.23 * | 25.13 * | 9.30 ns | 4.81 ns | 5.25 ns | 43.83 * | 18.70 ** |
| **Residuals** | 29 | 3.06 | 3.62 | 2.25 | 1.86 | 110.26 | 26.22 | 3.78 | 13.24 | 11.84 | 3.55 |

Significance levels: * and ** indicate the mean sum of squares are significant at p<0.05 and p<0.01 respectively, ns: Non-significant, *d*. *f*.: degrees of freedom

PH- Plant height(cm); PL- Panicle length(cm); NTP- Number of tillers per plant; NPP- Number of panicle per plant; TNG-Total number of grains per panicle; SF- Per cent spikelet fertility; TW-Test weight (g); SW- Straw weight (g); HI- Harvest index (%); GYP-Grain yield per plant (g)

**Supplementary Table S3 L. Estimation of genetic variability parameters for yield and yield-related traits in rice association panel at Dhadesugur under aerobic condition during *wet season* 2020.**

| **Traits** | **Range** | | **Mean** | **Genetic variability** | | ***h^2^*(bs)** (%) | **GAM** |
| --- | --- | --- | --- | --- | --- | --- | --- |
|  | **Min** | **max** |  | **GCV** | **PCV** |  |  |
| **PH** | 50.78 | 122.45 | 72.43 | 23.85 | 23.98 | 98.98 | 48.96 |
| **PL** | 12.40 | 26.15 | 21.14 | 11.41 | 14.53 | 61.61 | 18.47 |
| **NTP** | 3.44 | 18.13 | 12.59 | 22.36 | 25.34 | 77.85 | 40.69 |
| **NPP** | 3.16 | 13.78 | 10.43 | 16.85 | 21.32 | 62.48 | 27.48 |
| **TNG** | 33.67 | 185.83 | 125.98 | 24.84 | 26.21 | 89.88 | 48.59 |
| **SF** | 30.89 | 88.93 | 67.30 | 16.61 | 18.27 | 82.67 | 31.16 |
| **TW** | 9.75 | 26.21 | 17.27 | 21.37 | 24.16 | 78.26 | 39.01 |
| **SW** | 14.53 | 64.80 | 32.21 | 28.73 | 30.87 | 86.61 | 55.16 |
| **HI** | 9.57 | 44.30 | 25.78 | 28.60 | 31.56 | 82.11 | 53.46 |
| **GYP** | 3.32 | 21.12 | 10.81 | 27.21 | 32.32 | 70.91 | 47.28 |

PCV and GCV: Phenotypic and genotypic coefficient of variation**,** *h^2^*(bs): Heritability (broad sense), GAM: Genetic advance as *per cent* of mean

PH- Plant height (cm); PL- Panicle length (cm); NTP- Number of tillers per plant; NPP- Number of panicle per plant; TNG-total number of grains per panicle; SF-Per cent spikelet fertility; TW-Test weight (g); SW-Straw weight (g); HI- Harvest index (%); GYP- Grain yield per plant (g)

**Supplementary Table S4. A. Analysis of molecular variance (AMOVA) of the rice association panel.**

| **Source** | ***d*. *f*.** | **SS** | **MS** | **Est. Var.** | **%Var.** |
| --- | --- | --- | --- | --- | --- |
| **Among Pops** | 7.00 | 4.09 | 0.58 | 0.009 | 2.00% |
| **Within Pops** | 110.00 | 50.88 | 0.46 | 0.463 | 98.00% |
| **Total** | 117.00 | 54.98 |  | 0.472 | 100% |

*d*. *f*.: Degrees of freedom; SS: Sum of squares; MS: Mean sum of squares; Var: variance; %Var: *Per cent* variance

**Supplementary Table S4. B. Shannon statistics summary.**

| **Shannon Statistics Summary Table** | | | | | | | | |
| --- | --- | --- | --- | --- | --- | --- | --- | --- |
| **Source of Information** | ***d. f.*** | **Log-Like. Chi-Sq. G-Test** | **Shannon Information sH** | **Percent of Total Inform.** | **Diversity Estimate exp (sH)** | **[0,1] Scaled Diversity D'** | **[0,1] Scaled Overlap O'=1-D'** | **Estimated Probability P(rand >= data)** |
| **Among Pops** | 2 | 14.084 | 0.036 | 4.333 | 1.037 | 0.054 | 0.946 | 0.001 |
| **Within Pops** | 233 | 323.740 | 0.797 | 95.667 | 2.219 | 0.558 | 0.442 | 1.000 |
| **Total** | 235 | 337.824 | 0.833 | 100.000 | 2.301 | 0.568 | 0.432 |  |
| Based on Smouse, Whitehead & Peakall (2015) Mol Ecol Res | | | | |  |  |  |  |

**Supplementary Table S5. Polymorphic information content (PIC) of the polymorphic SSR markers.**

| **Sl. No.** | **Markers** | **Chr. No** | **Number of alleles** | **PIC** |
| --- | --- | --- | --- | --- |
| 1 | RM5933 | 8 | 4 | 0.7296 |
| 2 | RM16283 | 4 | 2 | 0.0780 |
| 3 | RM3524 | 4 | 2 | 0.4948 |
| 4 | RM17900 | 5 | 2 | 0.2466 |
| 5 | RM18182 | 5 | 3 | 0.5449 |
| 6 | RM18472 | 5 | 3 | 0.5256 |
| 7 | RM20023 | 6 | 2 | 0.2319 |
| 8 | RM18939 | 5 | 3 | 0.6390 |
| 9 | RM27879 | 12 | 2 | 0.4621 |
| 10 | RM26558 | 11 | 3 | 0.6626 |
| 11 | RM21749 | 7 | 2 | 0.4973 |
| 12 | RM16582 | 4 | 2 | 0.3569 |
| 13 | RM17263 | 4 | 3 | 0.6131 |
| 14 | RM20948 | 7 | 3 | 0.6620 |
| 15 | RM21165 | 7 | 3 | 0.4834 |
| 16 | RM22031 | 7 | 2 | 0.5000 |
| 17 | RM22763 | 8 | 3 | 0.3814 |
| 18 | RM22961 | 8 | 3 | 0.6520 |
| 19 | RM25310 | 10 | 4 | 0.7480 |
| 20 | RM28157 | 12 | 3 | 0.6593 |
| 21 | RM14472 | 3 | 2 | 0.4972 |
| 22 | RM24842 | 9 | 2 | 0.5000 |
| 23 | RM38 | 8 | 2 | 0.4548 |
| 24 | RM1201 | 1 | 2 | 0.4386 |
| 25 | RM1385 | 2 | 3 | 0.4214 |
| 26 | RM5179 | 2 | 2 | 0.5000 |
| 27 | RM6100 | 10 | 3 | 0.6000 |
| 28 | RM7097 | 3 | 2 | 0.4072 |
| 29 | RM10039 | 1 | 2 | 0.4444 |
| 30 | RM10149 | 1 | 2 | 0.3641 |
| 31 | RM15981 | 3 | 2 | 0.2612 |
| 32 | RM242 | 9 | 2 | 0.4444 |
| 33 | RM474 | 10 | 3 | 0.6485 |
| 34 | RM17377 | 4 | 2 | 0.4535 |
| 35 | RM284 | 8 | 2 | 0.0496 |
| 36 | RM25756 | 10 | 2 | 0.5000 |
| 37 | RM27499 | 12 | 2 | 0.5000 |
| 38 | RM20698 | 6 | 4 | 0.7497 |
| 39 | RM17377 | 4 | 2 | 0.3912 |
| 40 | RM3187 | 6 | 2 | 0.1264 |
| 41 | RM1149 | 5 | 2 | 0.3691 |
| 42 | RM12434 | 2 | 2 | 0.4999 |
| 43 | RM14753 | 3 | 4 | 0.7381 |
| 44 | RM467 | 10 | 2 | 0.3772 |
| 45 | RM13155 | 2 | 2 | 0.5000 |
| 46 | RM3029 | 3 | 2 | 0.3074 |
| 47 | RM566 | 9 | 2 | 0.0504 |
| 48 | RM13962 | 2 | 4 | 0.5447 |
| 49 | RM19179 | 5 | 2 | 0.5000 |
| 50 | RM7083 | 6 | 2 | 0.2702 |
| 51 | RM6837 | 3/12 | 3 | 0.3508 |
| 52 | RM7086 | 1 | 2 | 0.0496 |
| 53 | RM3455 | 12 | 3 | 0.1251 |
| 54 | RM6107 | 2 | 3 | 0.2289 |
| 55 | RM7009 | 2 | 2 | 0.4994 |
| 56 | RM202 | 11 | 2 | 0.4718 |
| 57 | RM1015 | 12 | 2 | 0.4972 |
| 58 | RM262 | 2 | 2 | 0.4032 |
| 59 | RM105 | 9 | 2 | 0.4932 |
| 60 | RM168 | 3 | 2 | 0.4950 |
| 61 | RM252 | 4 | 3 | 0.5277 |
| 62 | RM410 | 9 | 3 | 0.6409 |
| 63 | RM520 | 3 | 2 | 0.4940 |
| 64 | RM1146 | 10 | 2 | 0.4737 |
| 65 | RM1388 | 4 | 2 | 0.4718 |
| 66 | RM109 | 2 | 2 | 0.0655 |
| 67 | RM229 | 11 | 2 | 0.3488 |
| 68 | RM106 | 2 | 2 | 0.1576 |
| 69 | RM3117 | 3 | 2 | 0.4555 |
| 70 | RM5300 | 2 | 2 | 0.1490 |
| 71 | RM5501 | 1 | 2 | 0.4989 |
| 72 | RM16 | 3 | 2 | 0.3530 |
| 73 | RM80 | 8 | 4 | 0.6336 |
| 74 | RM3430 | 6 | 2 | 0.0868 |
| 75 | RM7006 | 2 | 2 | 0.0812 |
| 76 | RM1388 | 4 | 2 | 0.4352 |
| 77 | RM2584 | 8 | 2 | 0.4982 |
| 78 | RM5944 | 3 | 2 | 0.0846 |
| 79 | RM8111 | 1 | 2 | 0.1431 |
| 80 | RM6849 | 3 | 3 | 0.4799 |
| 81 | RM7075 | 1 | 2 | 0.4215 |
| 82 | RM6283 | 3 | 2 | 0.3367 |
| 83 | RM20069 | 6 | 2 | 0.4816 |
| 84 | RM16030 | 3 | 2 | 0.4744 |
| 85 | RM3183 | 6 | 2 | 0.1528 |
| 86 | RM6144 | 10 | 2 | 0.0555 |
| 87 | RM5 | 1 | 2 | 0.1993 |
| 88 | RM224 | 11 | 2 | 0.4581 |
| 89 | RM3825 | 1 | 2 | 0.4002 |
| 90 | RM3217 | 4 | 2 | 0.4912 |
| 91 | RM3709 | 1 | 2 | 0.3307 |
| 92 | RM6872 | 7 | 2 | 0.0655 |
| 93 | RM4455 | 10 | 2 | 0.4902 |
| 94 | RM1112 | 4 | 2 | 0.4997 |
| 95 | RM2584 | 8 | 3 | 0.5738 |
| 96 | RM1141 | 1 | 2 | 0.3846 |
| 97 | RM3188 | 2 | 2 | 0.1944 |
