# Supplementary material for: Genome-wide association studies in rice germplasm reveal significant genomic regions for root and yield-related traits under aerobic and irrigated conditions
Source: Front Plant Sci. 2023 Jul 18;14:1143853. doi: 10.3389/fpls.2023.1143853 (PMC10395336; doi:10.3389/fpls.2023.1143853)
Supplement: Supplementary file 1 [file DataSheet_1.zip › Additional File 1 Box Plots.docx]

Additional File 1


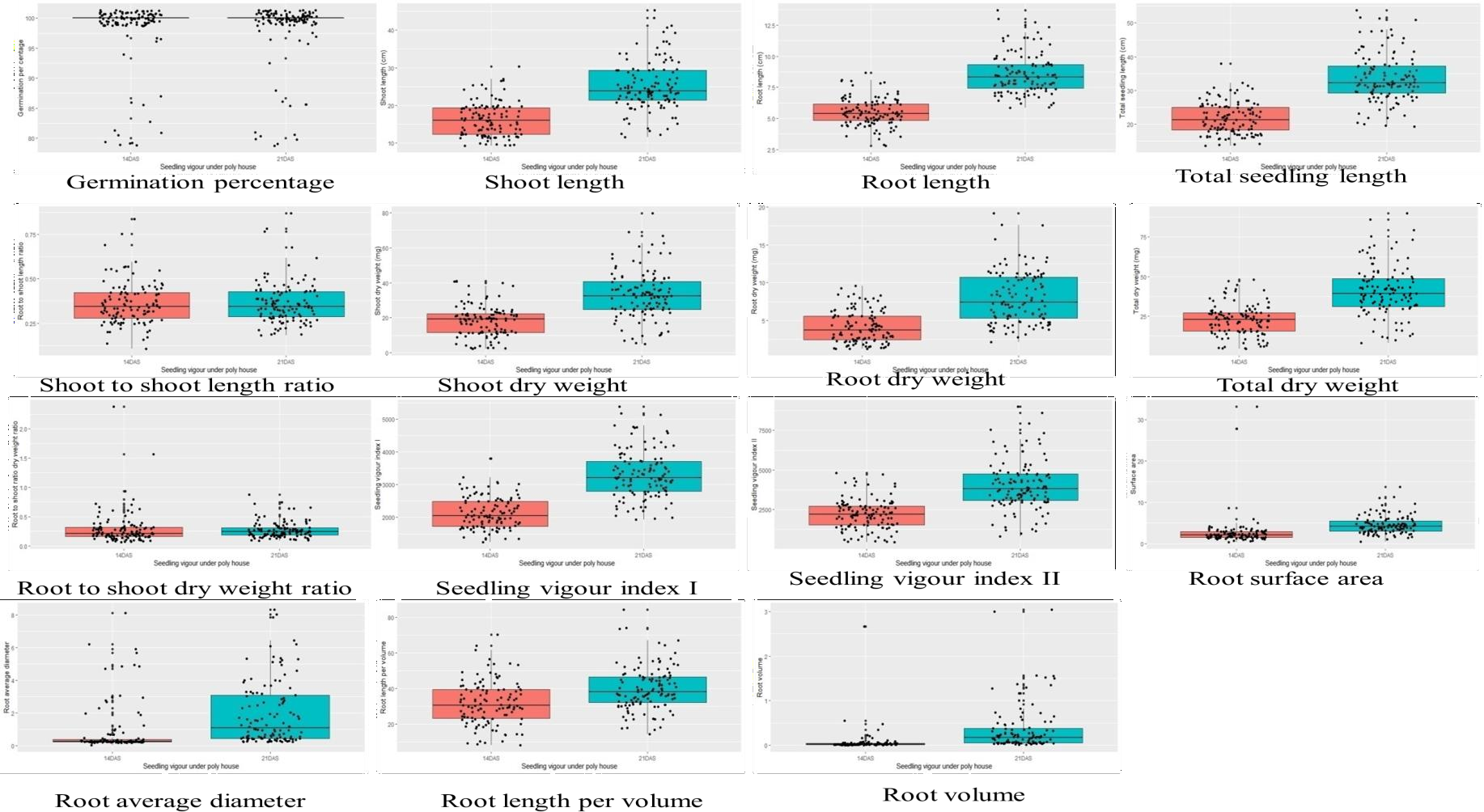


Additional Fig 1. Box plots showing frequency distribution for seedling vigour index traits in rice association panel at 14 and 21 DAS under poly house condition during 2018.


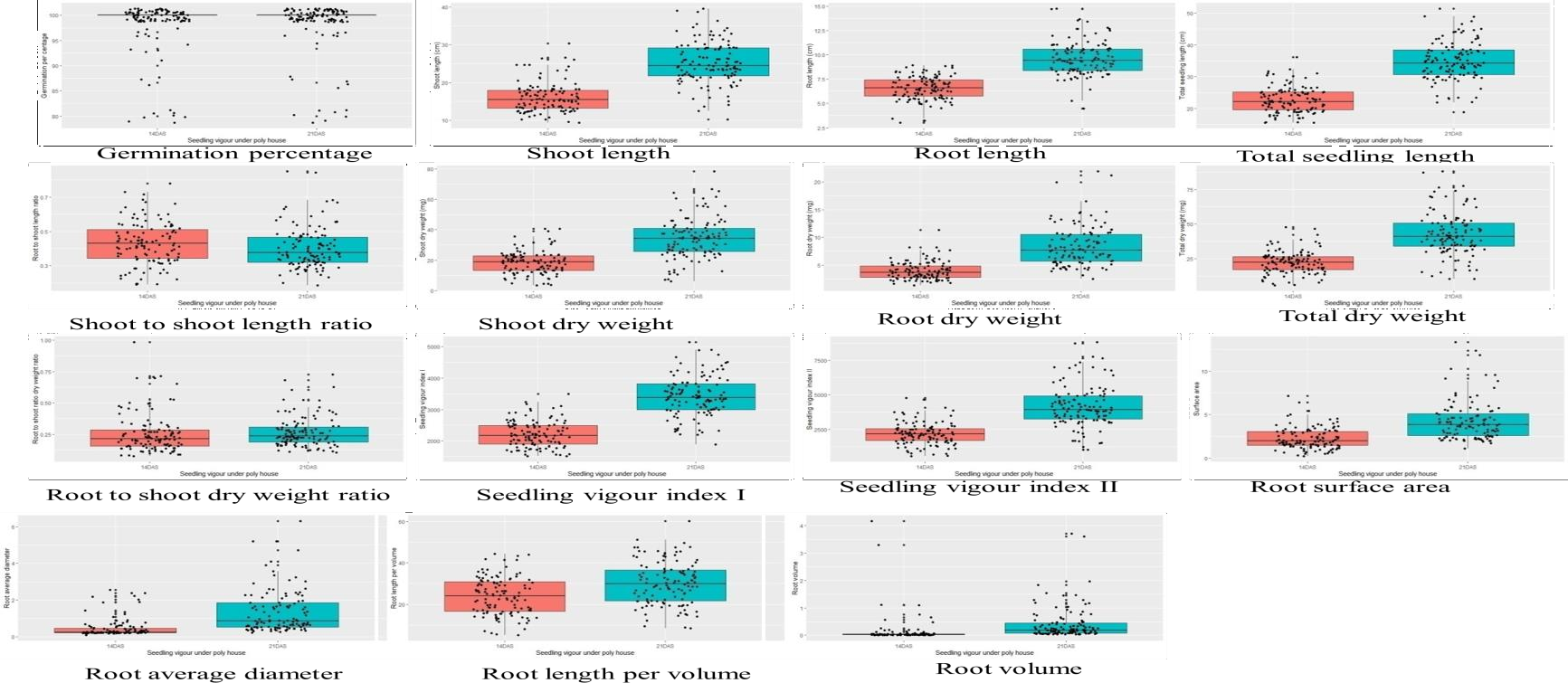


Additional Fig 2 Box plots showing frequency distribution for seedling vigour index traits in rice association panel at 14 and 21 DAS under poly house condition during 2019.


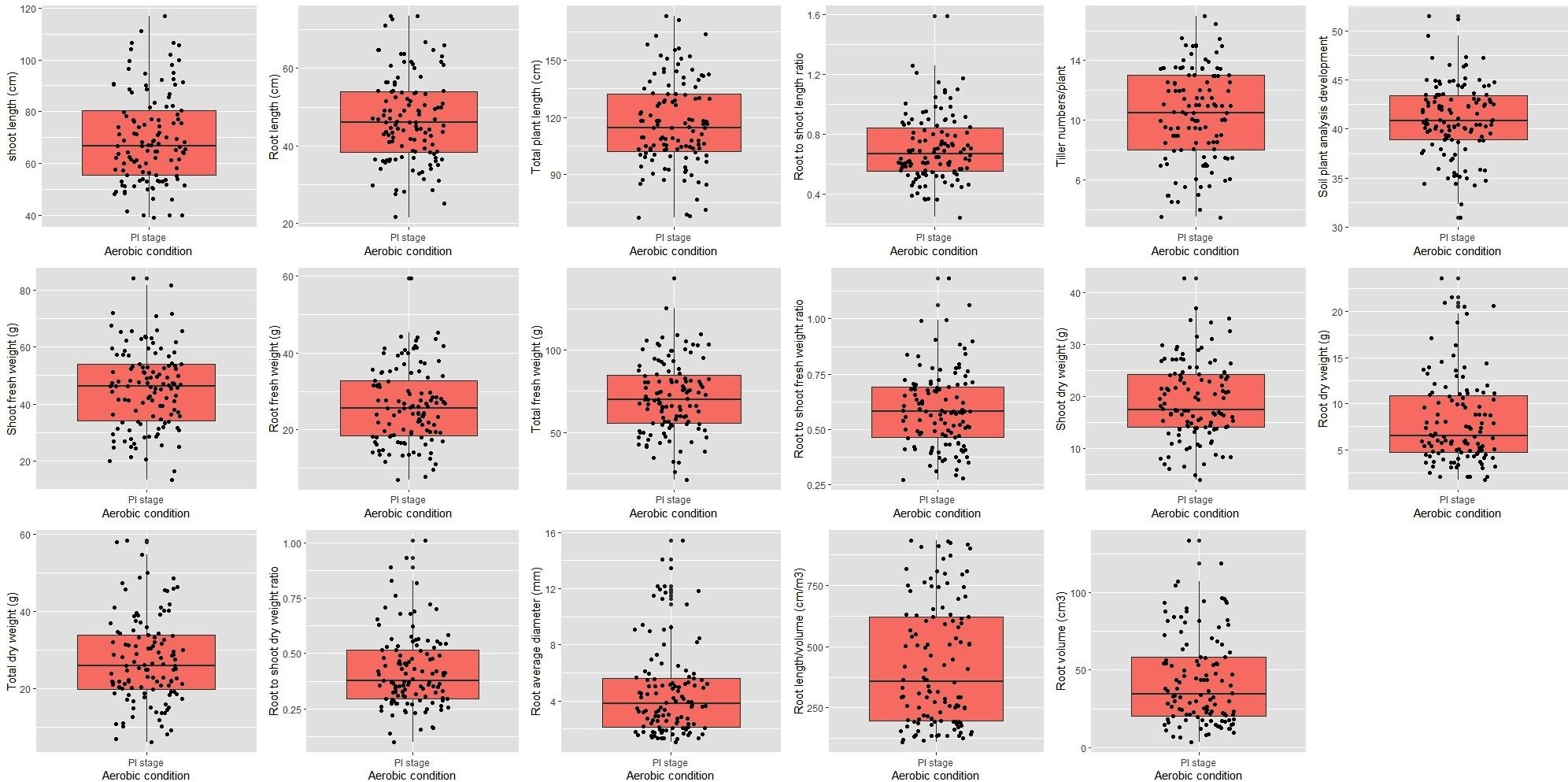


Additional Fig 3 Box plots showing frequency distribution for root traits in rice association panel at panicle initiation stage under polyhouse condition during 2019.


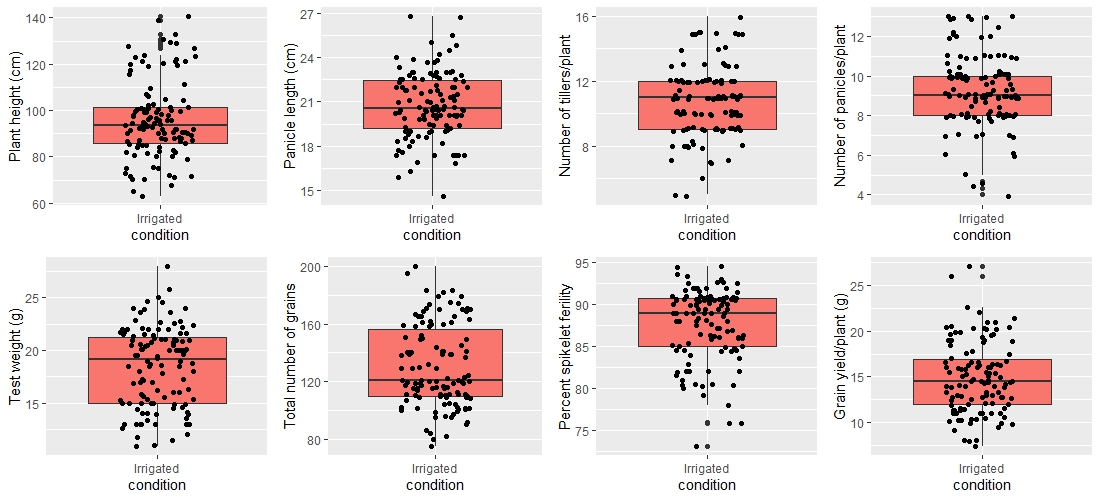


**Additional Fig 4 Box plots showing frequency distribution for yield and yield-related traits in rice association panel for irrigated condition at ICAR-IIRR during *wet* season 2019.**

Panicle length (*dry*)

Plant height (*dry*)

Plant height (*wet*)


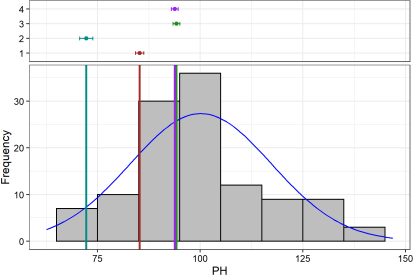

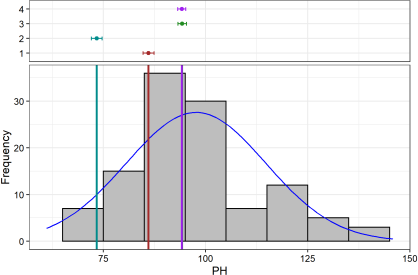

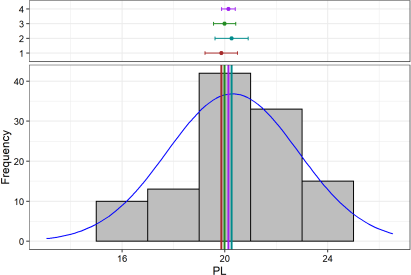


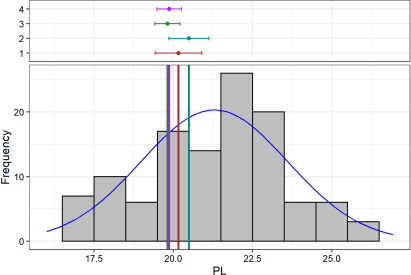


Panicle length (*wet*)


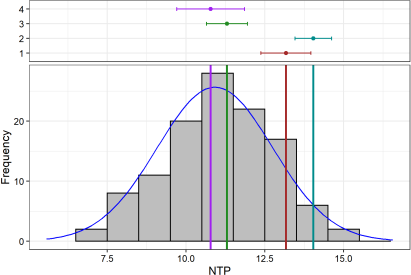

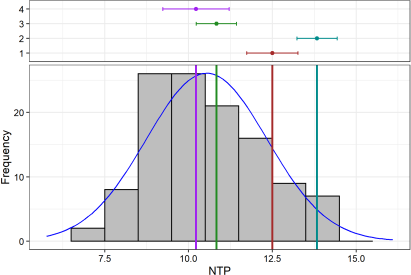

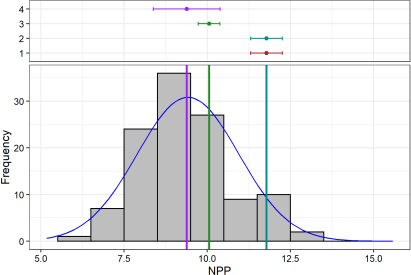

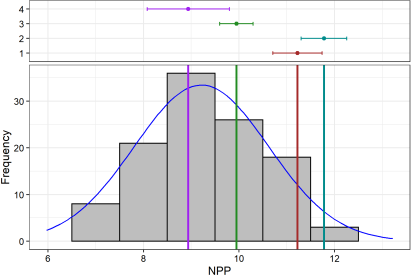


Number of tillers/plant (*wet*)

Number of tillers/plant (*dry*)

Number of panicles/plant (*wet*)

Number of panicles/plant (*dry*)


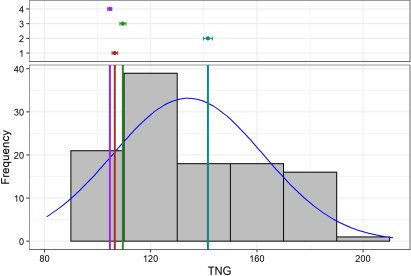

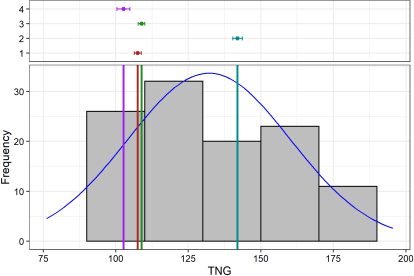

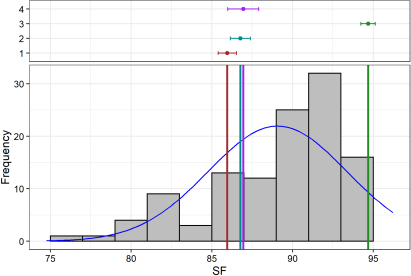

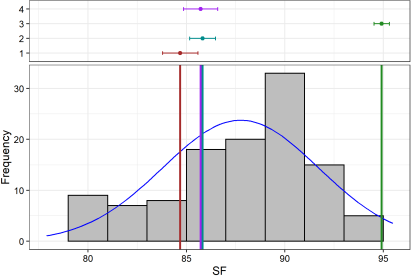


Total number of grains/panicle (*wet*)

Total number of grains/panicle (*dry*)

Spikelet fertility % (*wet*)

Spikelet fertility % (*dry*)


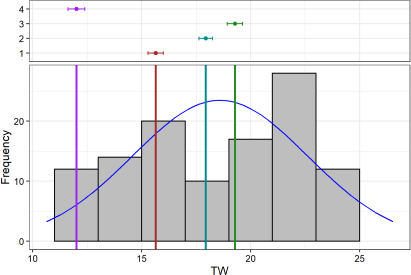

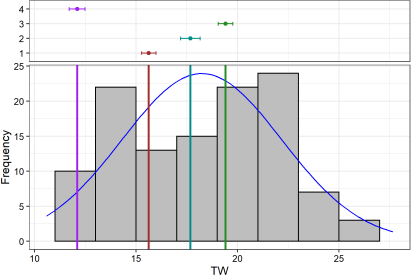

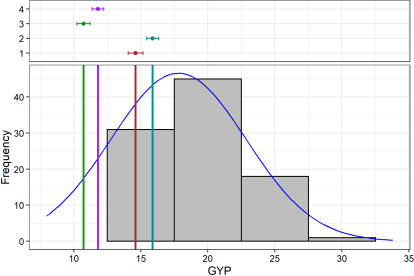

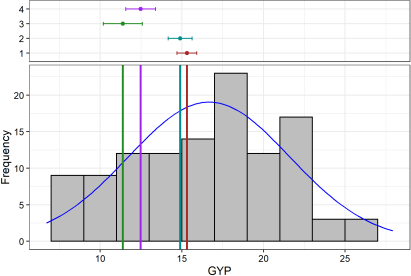


Test weight (*wet*)

Test weight (*dry*)

Grain yield/plant (*wet*)

Grain yield/plant (*dry*)

Additional Fig 5 Box plots showing frequency distribution for yield and yield-related traits in rice association panel for irrigated

condition at ICAR-IIRR during *wet season* and *dry season* 2020.

Checks are marked by red bars: where, the brown bar indicates BPT-5204, dark cyan bar indicates Swarna, the forest green bar indicates

MTU-1010, the purple bar indicates RNR-15048.


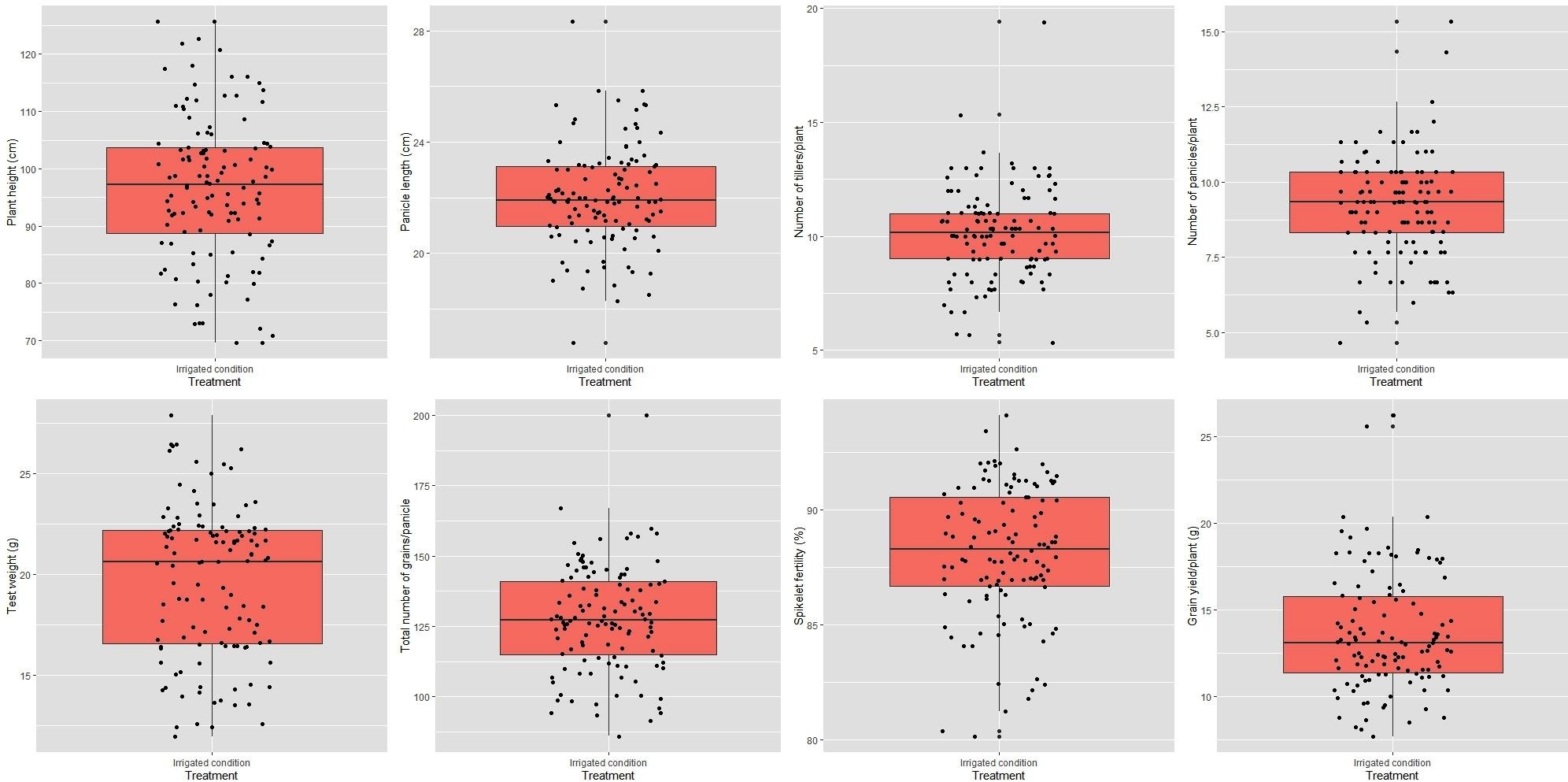


Additional Fig 6 Box plots showing frequency distribution for yield and yield-related traits in rice association panel under irrigated condition at ARS, Dhadesugur during *wet season* 2020.


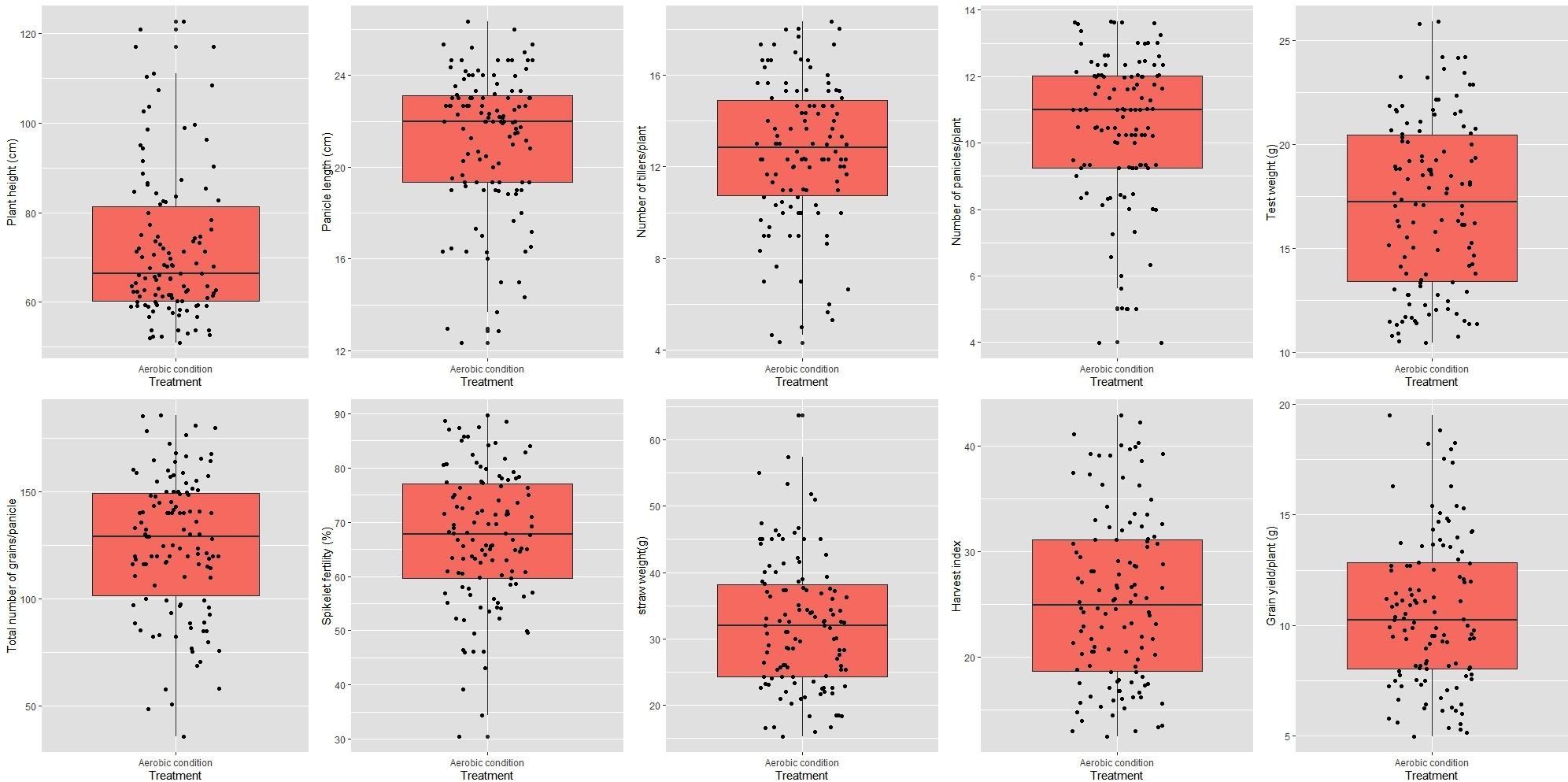


Additional Fig 7 Box plots showing frequency distribution for yield and yield-related traits in rice association panel at ARS, Dhadesugur under aerobic condition during the *wet season* 2020.
