# Supplementary material for: Genome-wide association studies in rice germplasm reveal significant genomic regions for root and yield-related traits under aerobic and irrigated conditions
Source: Front Plant Sci. 2023 Jul 18;14:1143853. doi: 10.3389/fpls.2023.1143853 (PMC10395336; doi:10.3389/fpls.2023.1143853)
Supplement: Supplementary file 1 [file DataSheet_1.zip › Supplementary Figures.docx]

**Supplementary Figures**

B

A


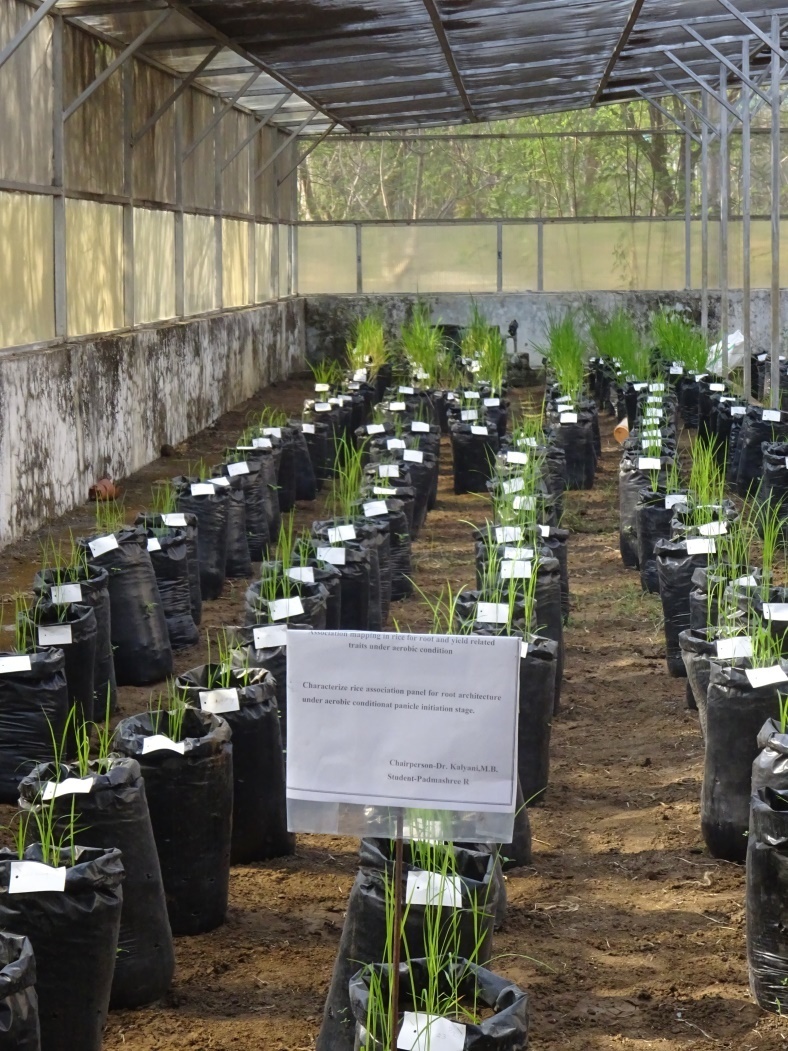

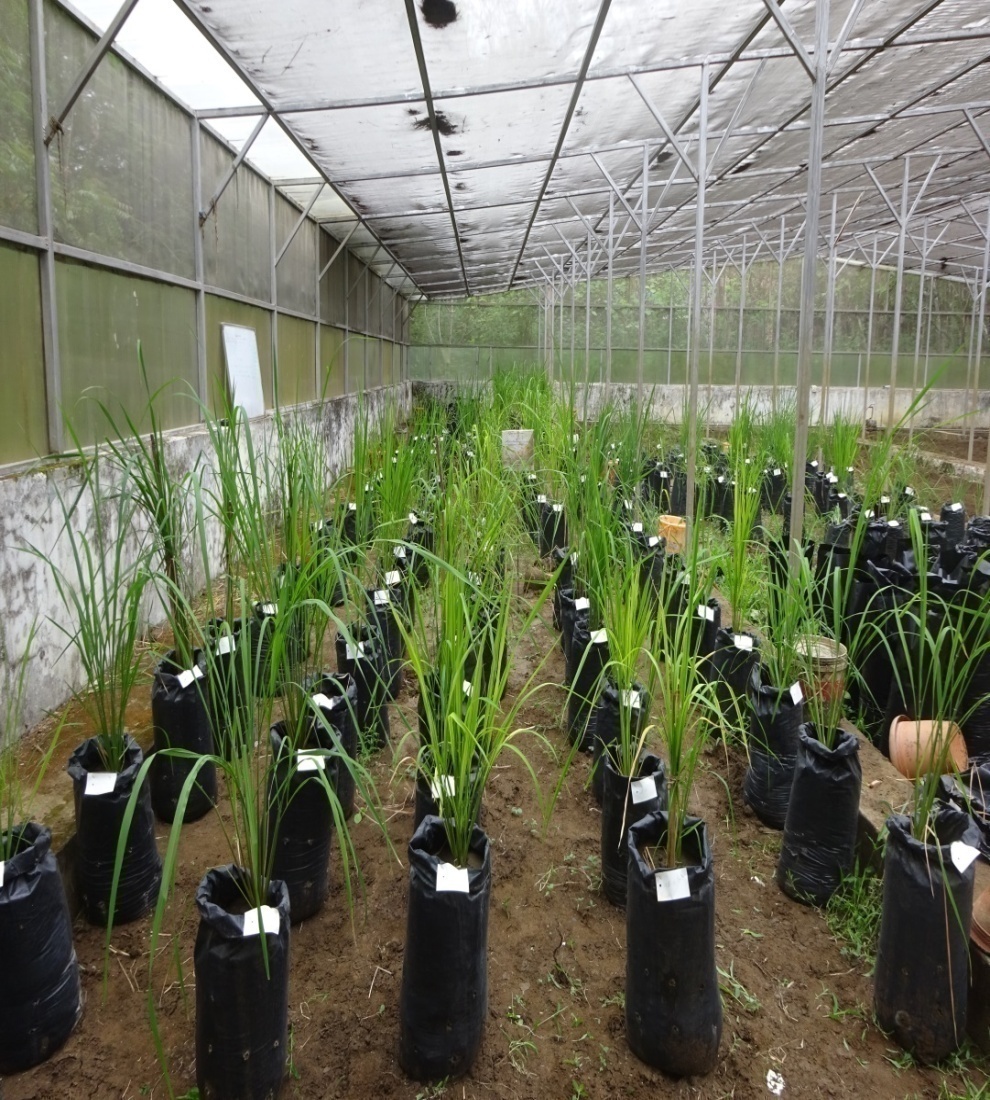


**Supplementary Figure. S1. Experimental set-up for root studies at the polyhouse under aerobic condition (A) At seedling stage (B) At the panicle initiation stage.**


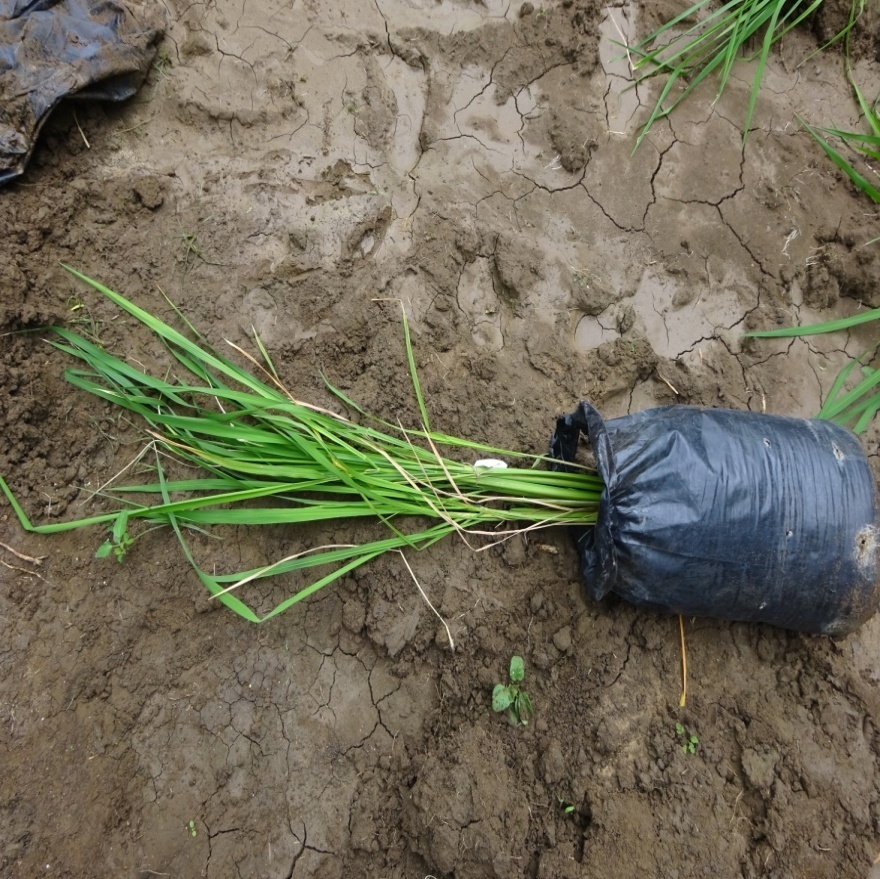

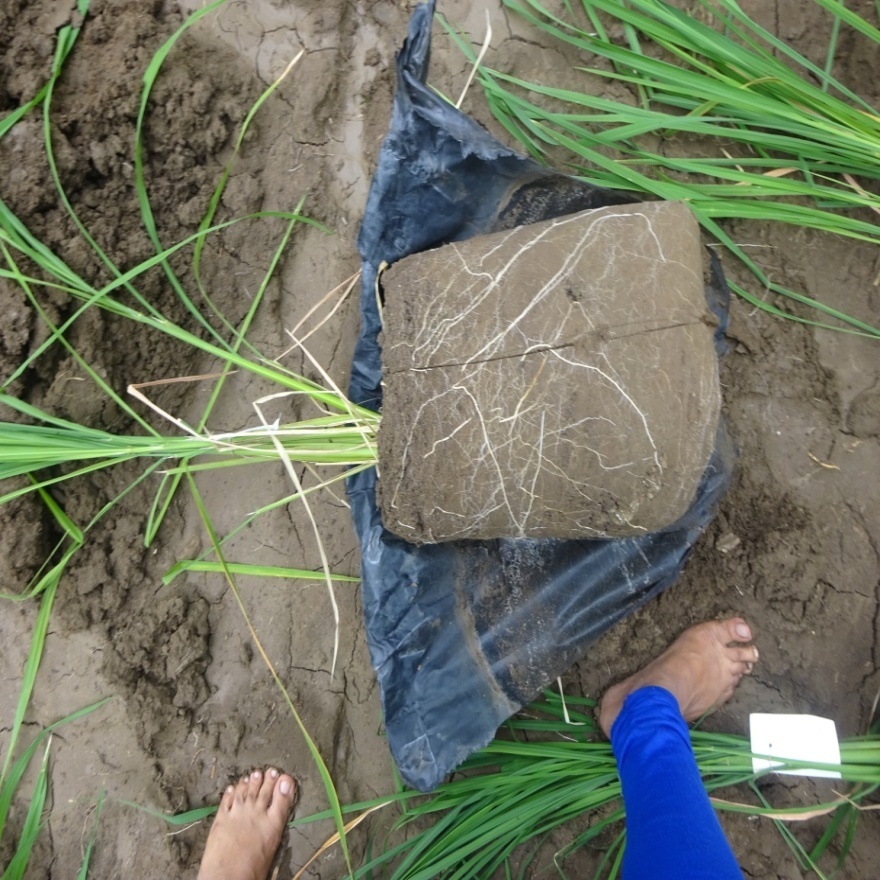


**Supplementary Figure. S2. Root washing and phenotyping at the panicle initiation stage.**

| KJ-214  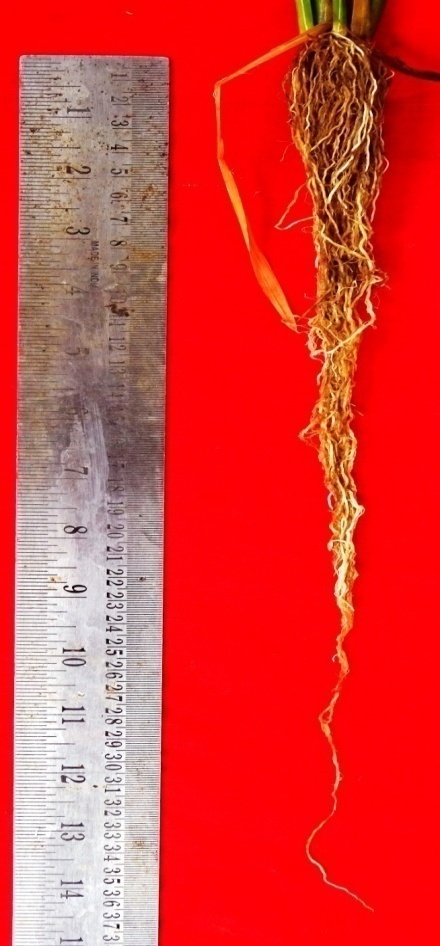 | KJ—216  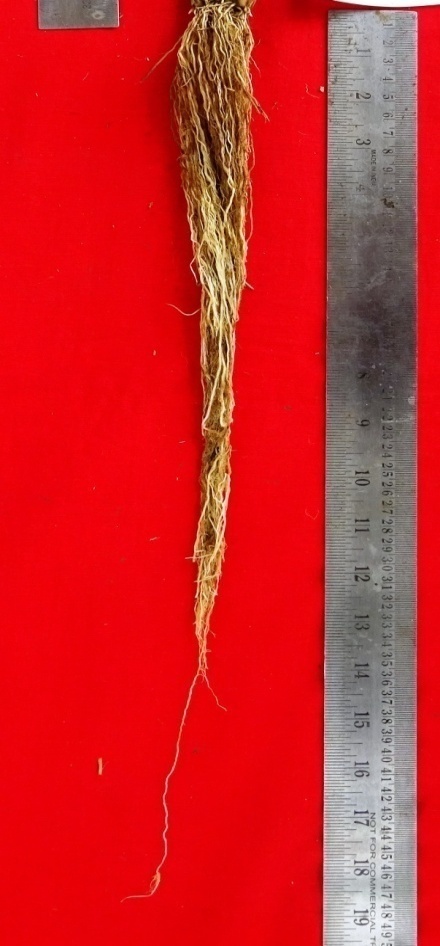 | KJ-219  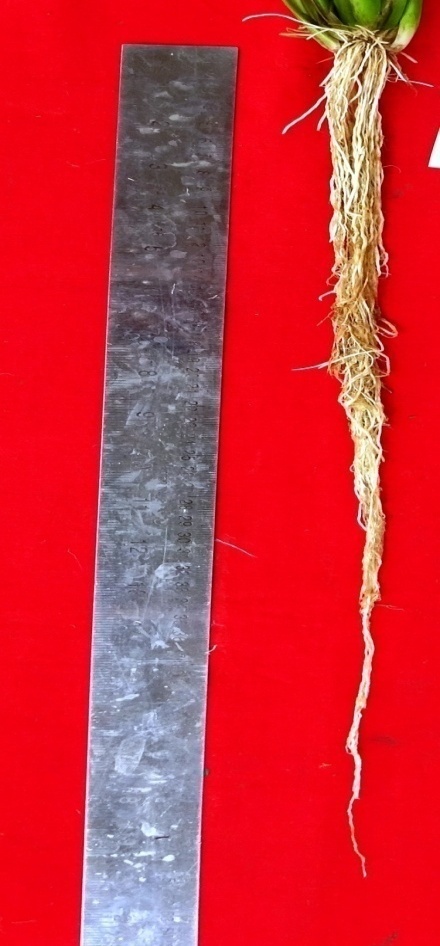 |
| --- | --- | --- |
| KJ-221  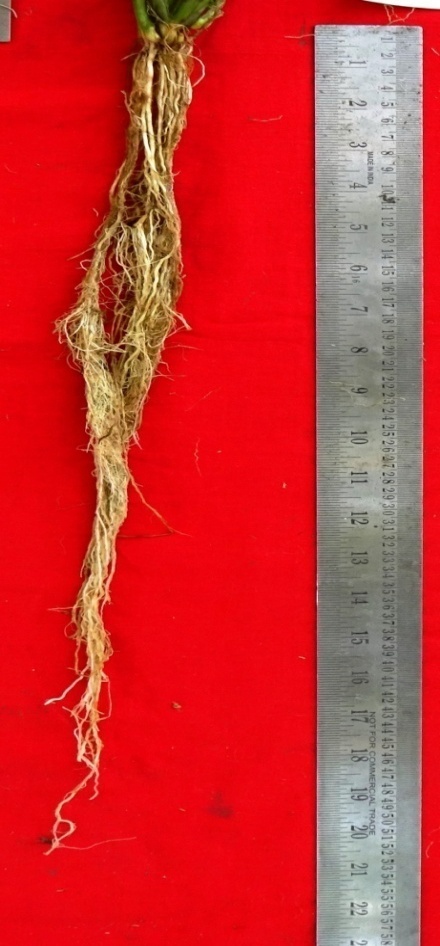 | KJ-222 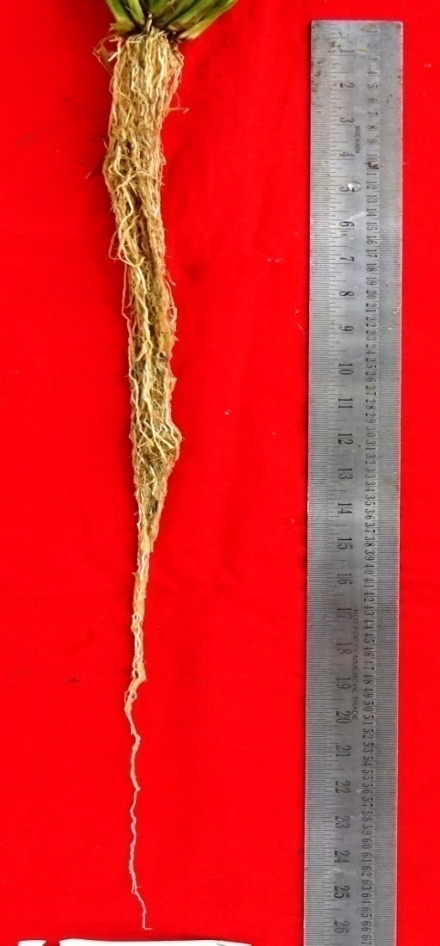 | KJ-226 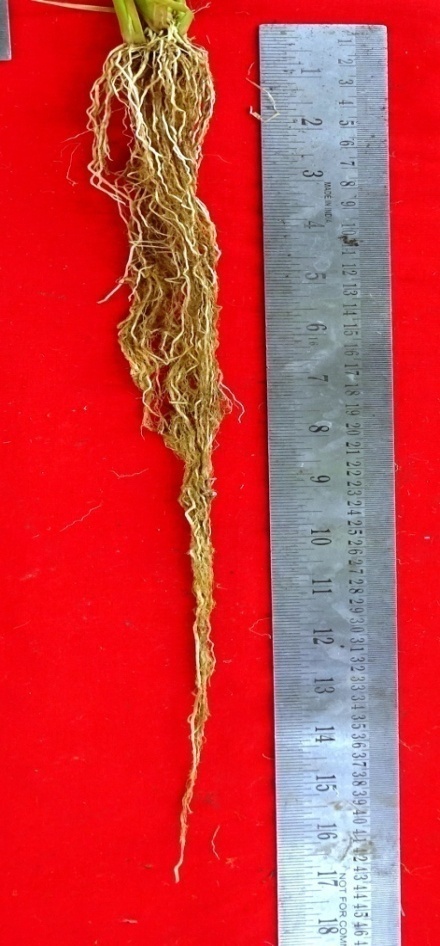 |
| WB-3  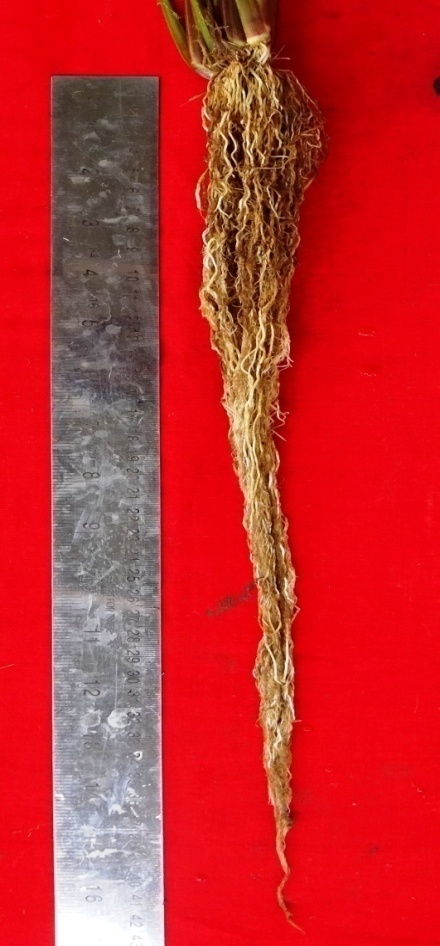 | WB-5  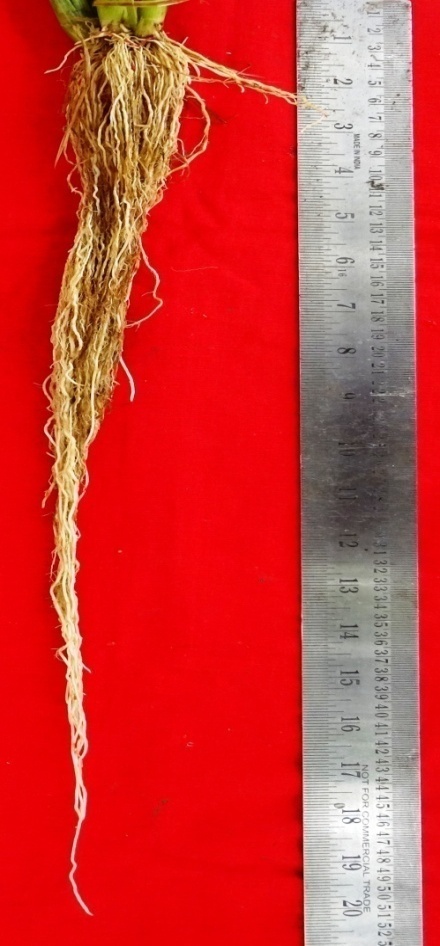 | WB-6  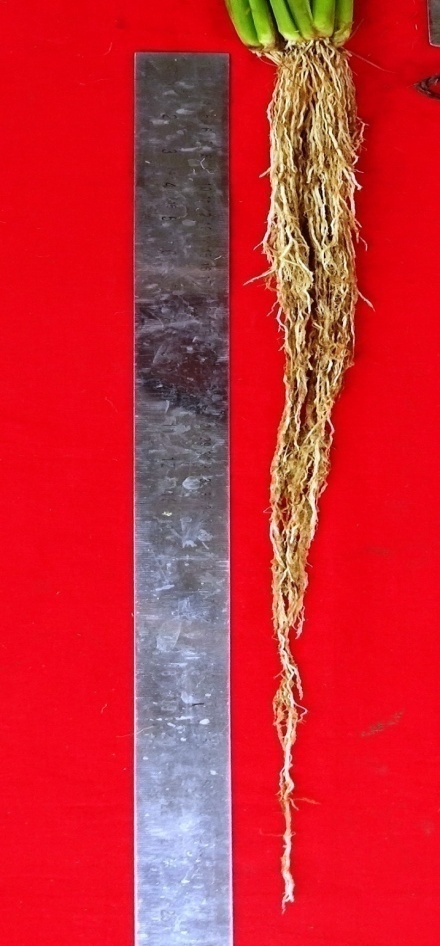 |
| WB-8  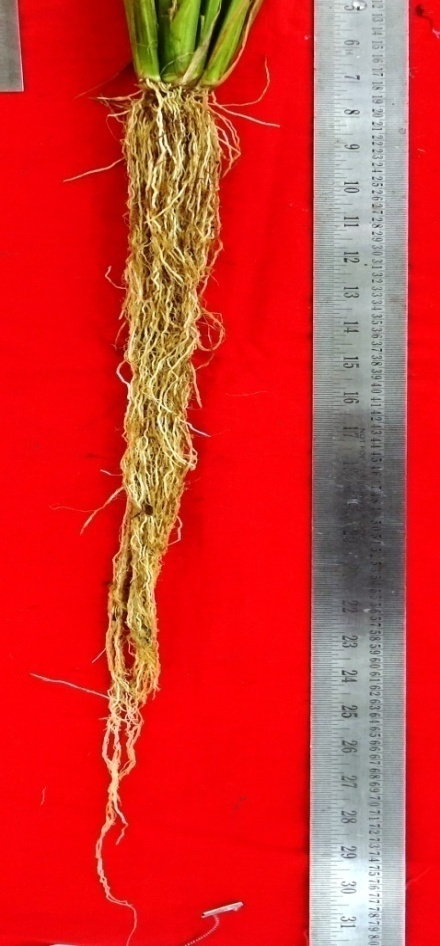 | WB-10 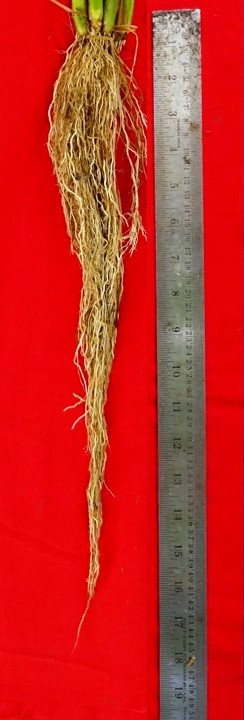 | WB-12 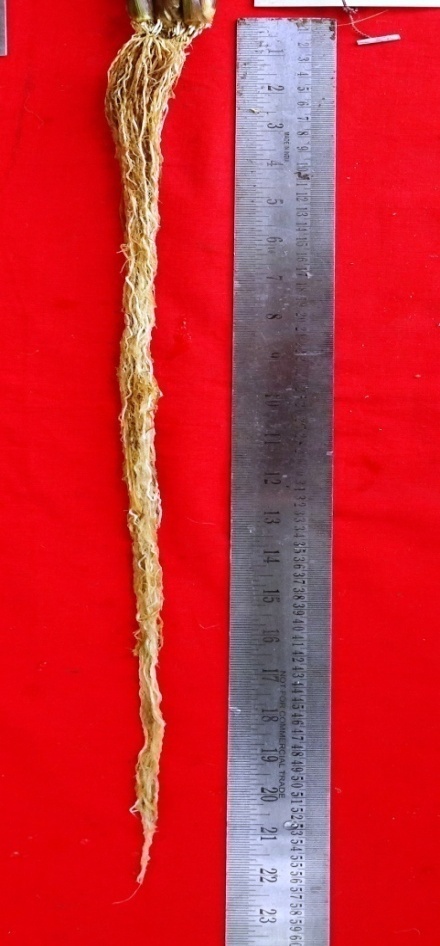 |
| WB-14 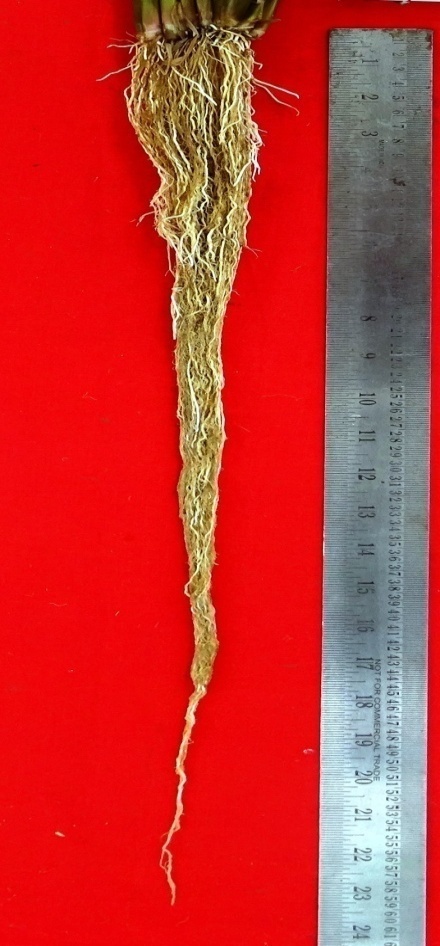 | WB-15  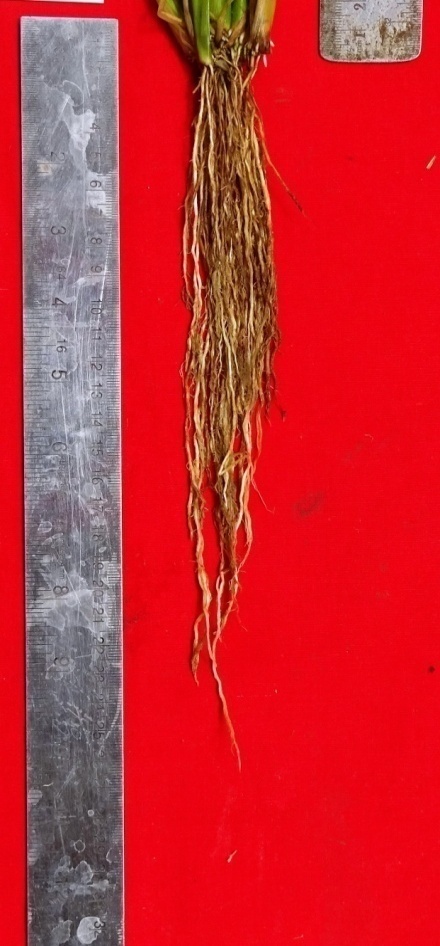 | WB-16 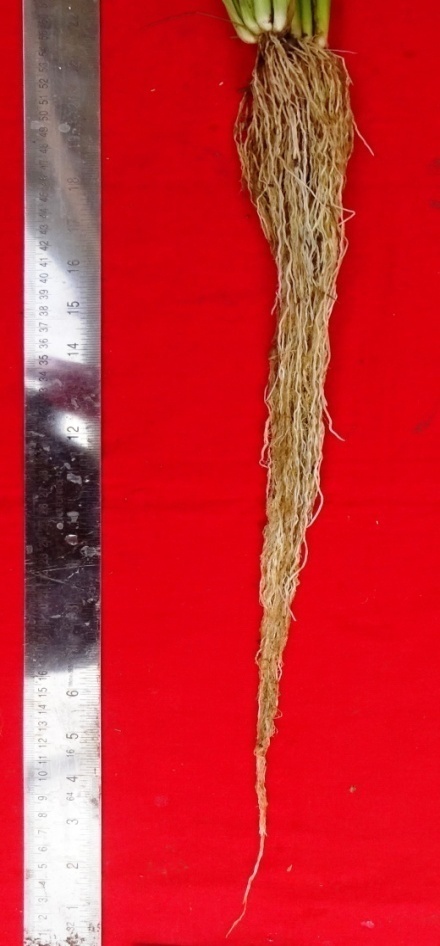 |
| WB-22  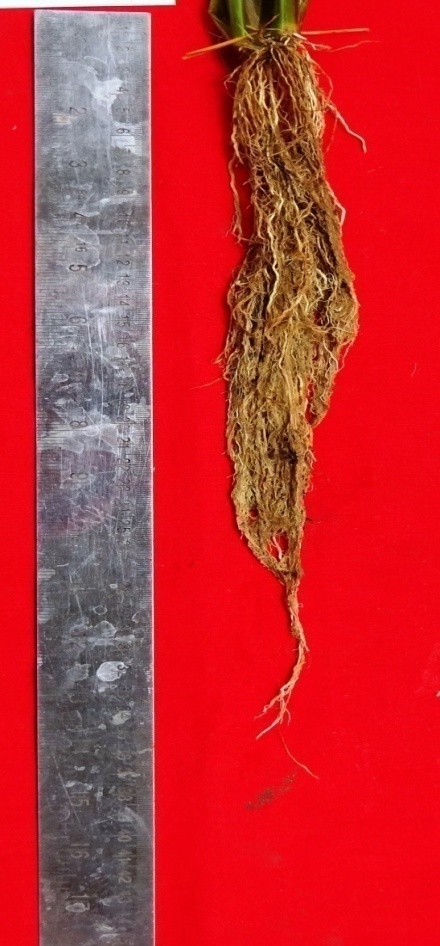 | WB-23 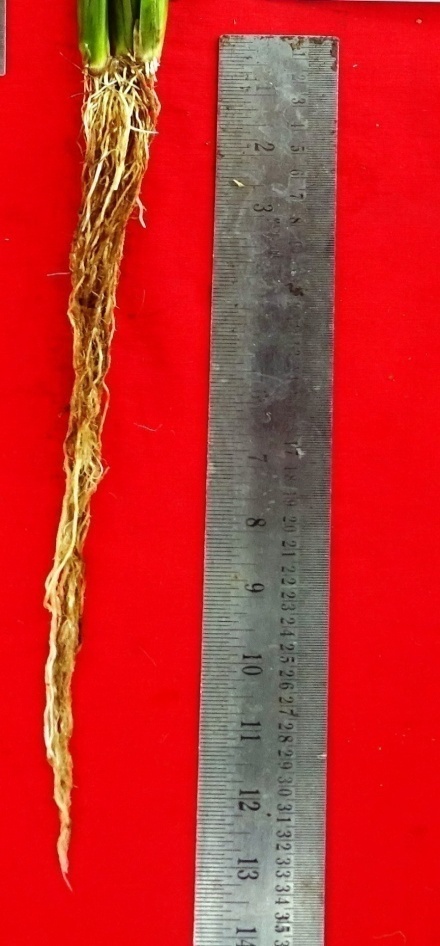 | WB-24  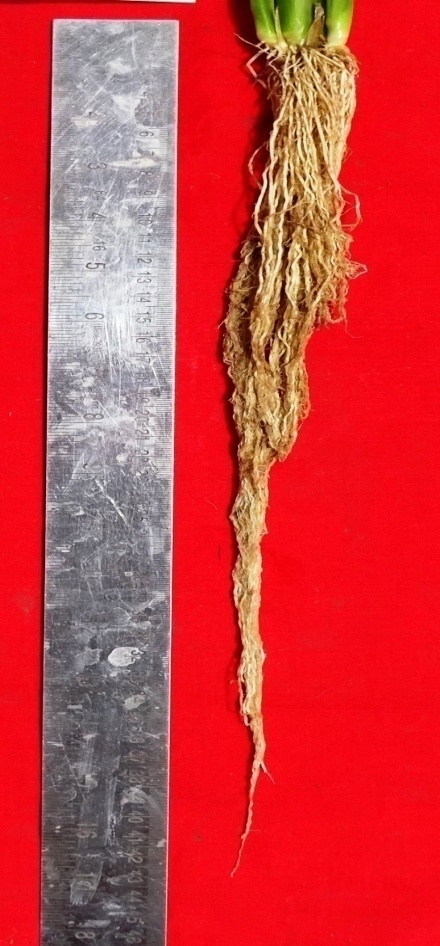 |
| WB-26  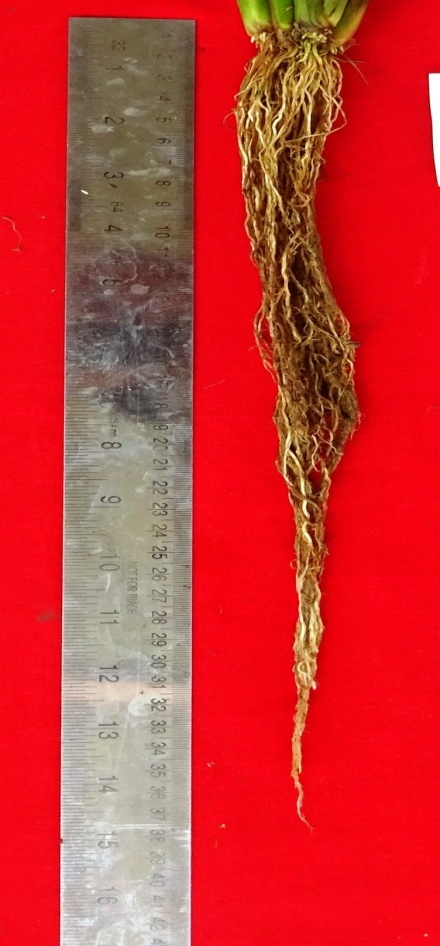 | WB-27 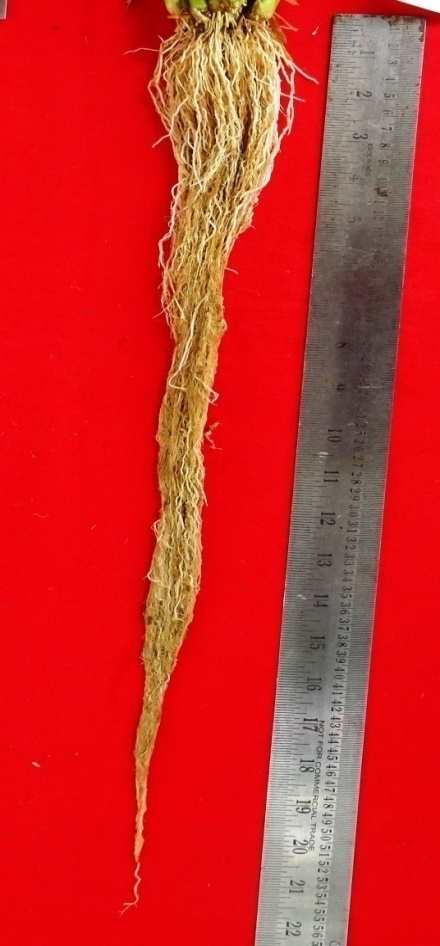 | WB-29 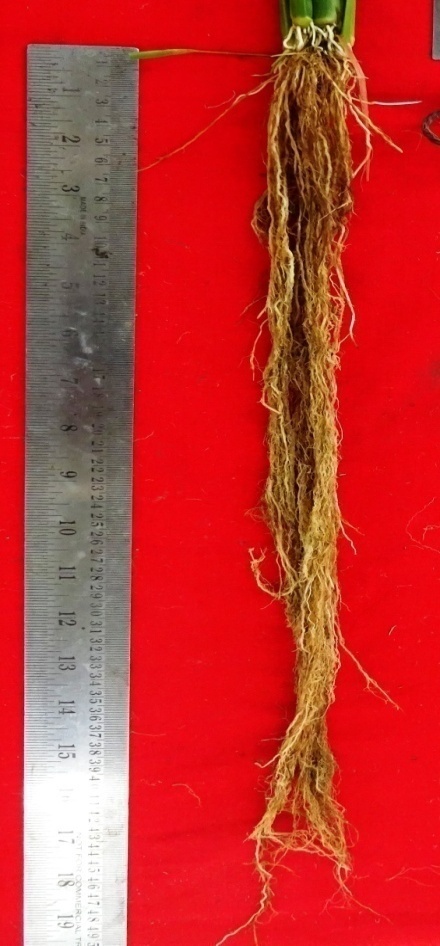 |
| WB-30  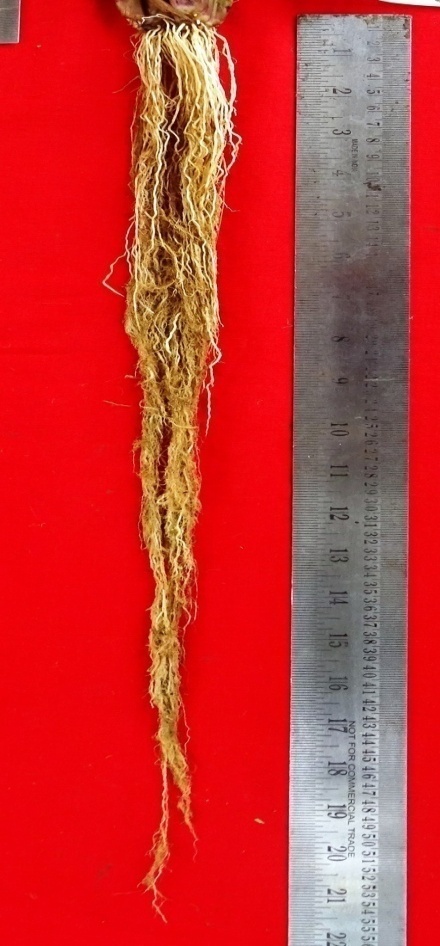 | WB-32 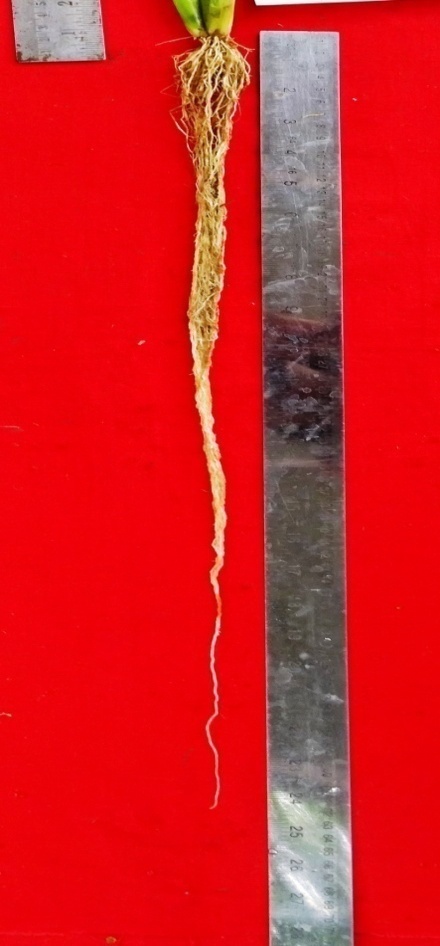 | WB-39  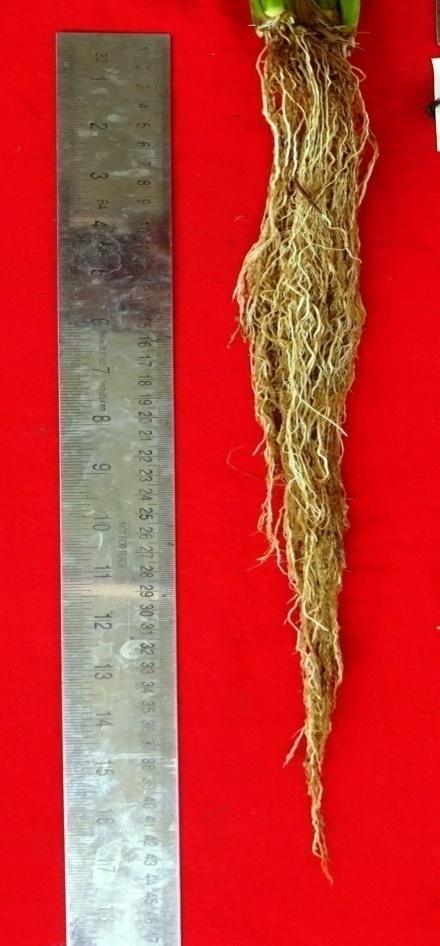 |
| GNV-1109  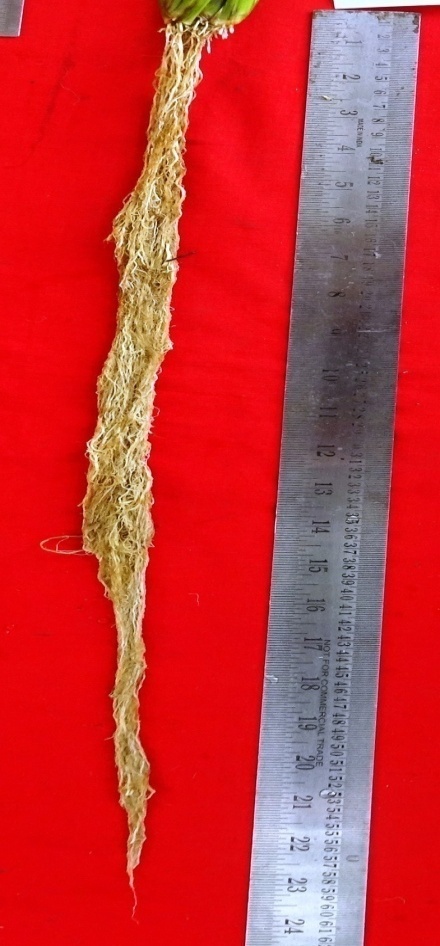 | GNV-1089 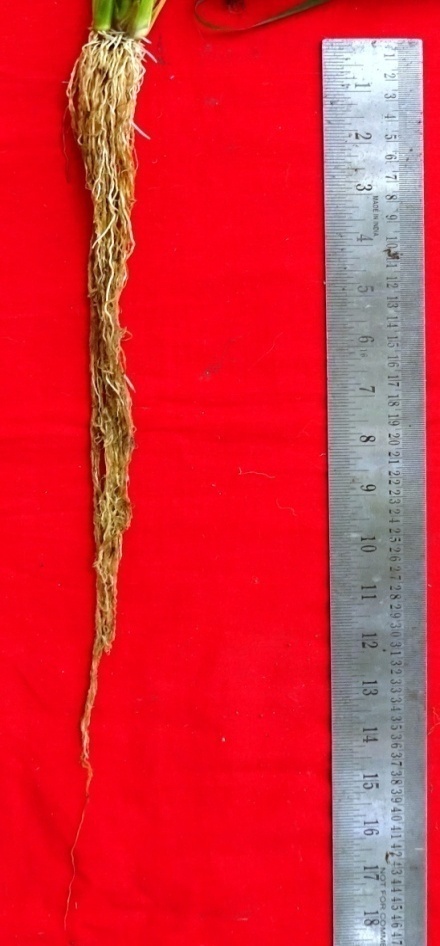 | RNR-15048  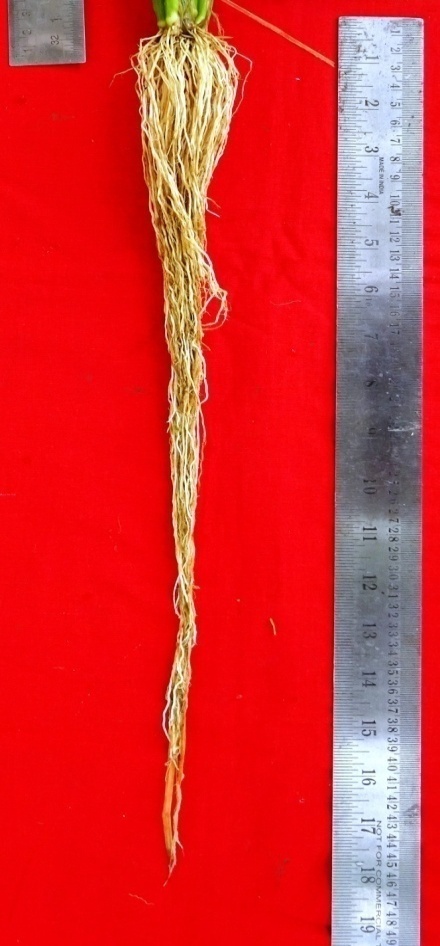 |
| POKKALI  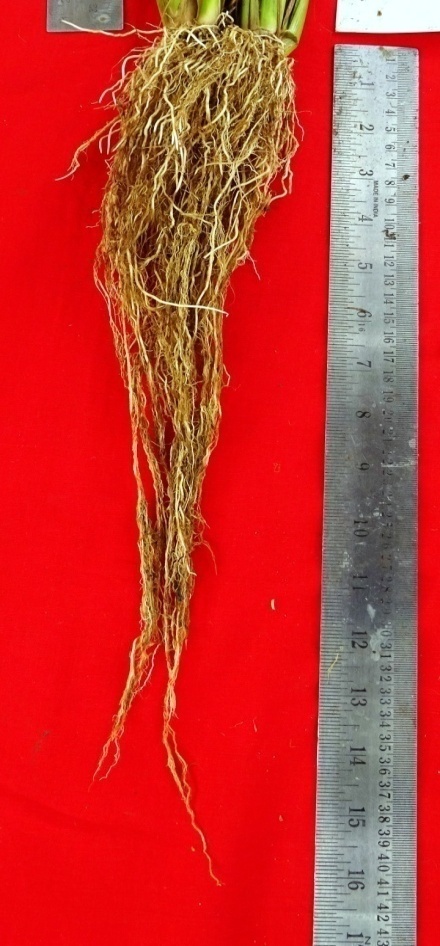 | SIRI-1253 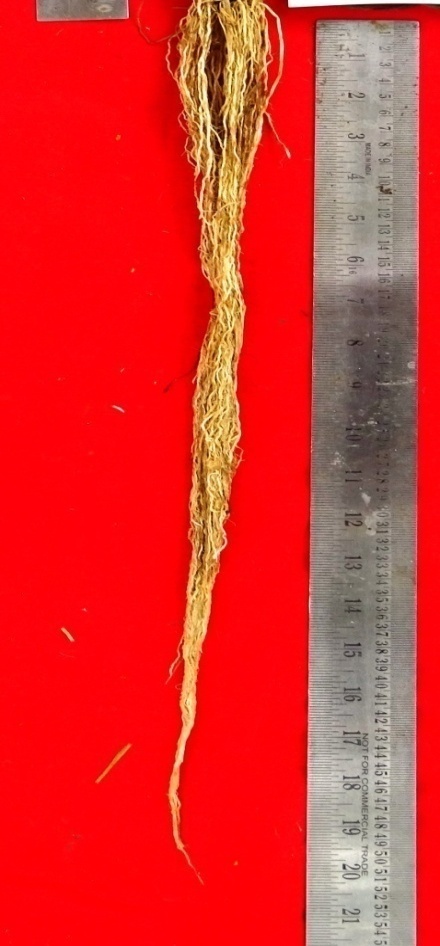 | GNV-14-96-1 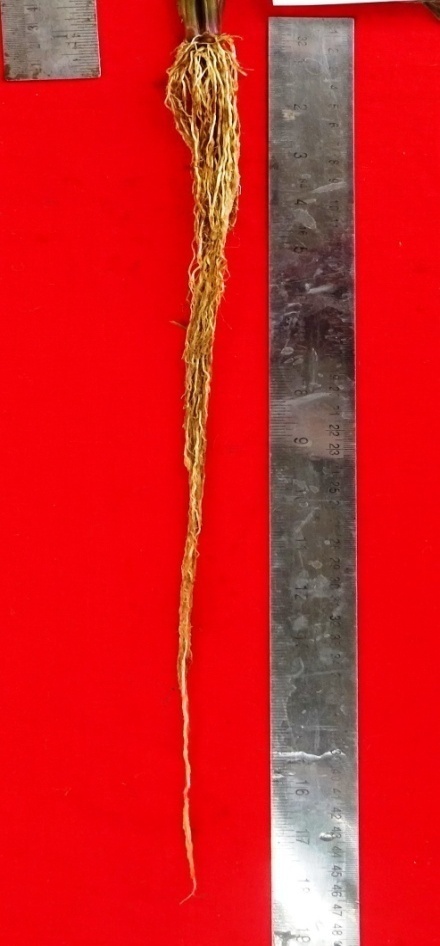 |
| RP-BIO  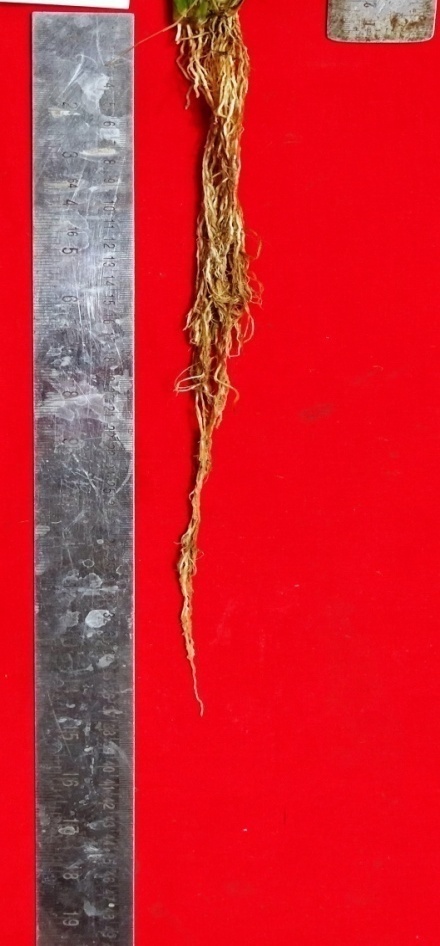 | TELLAHAMSA  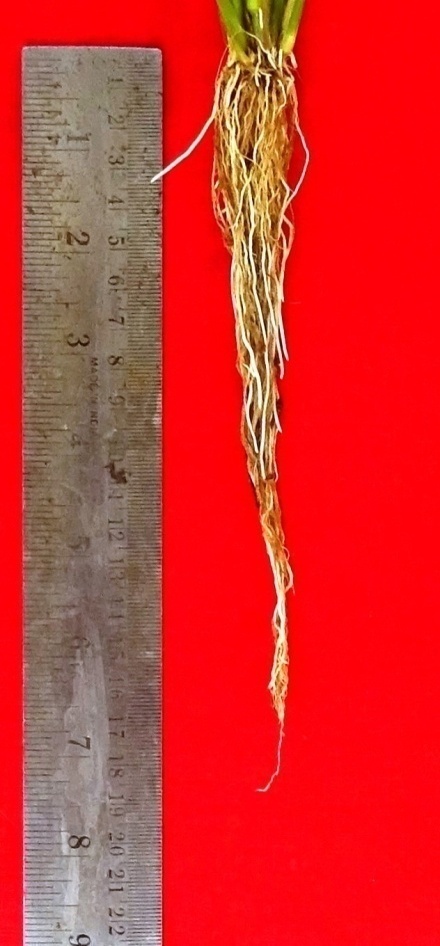 | FL-478 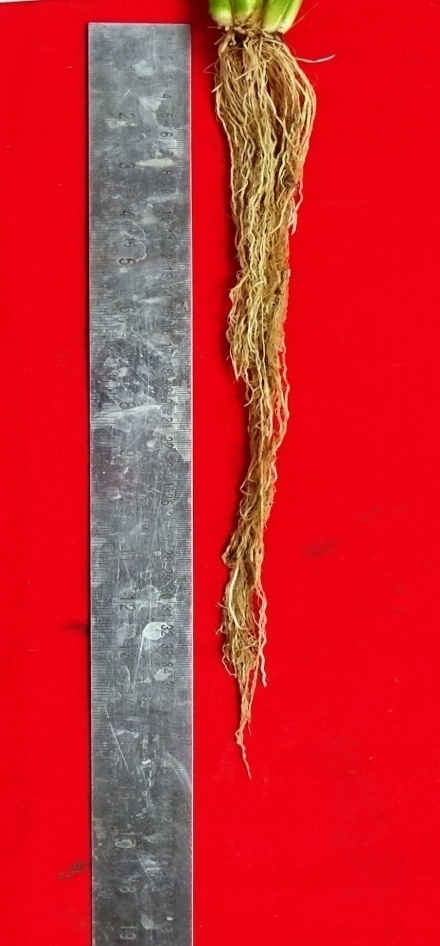 |
| RATNAMUDI  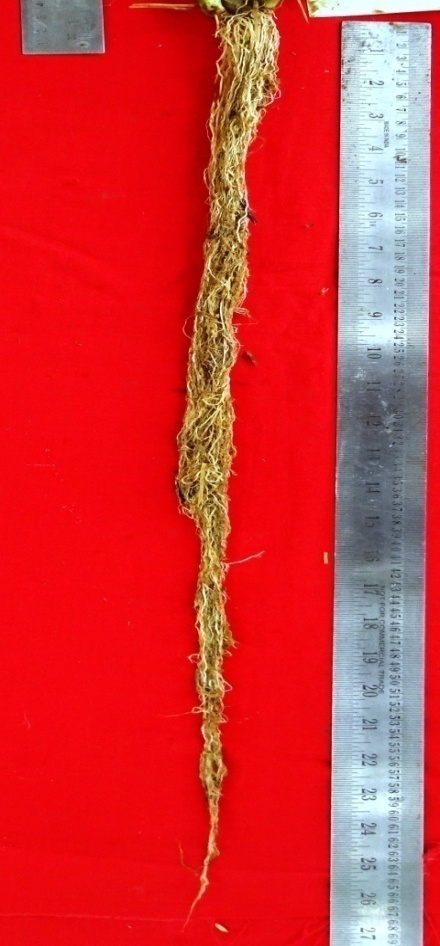 | RATNACHUDI 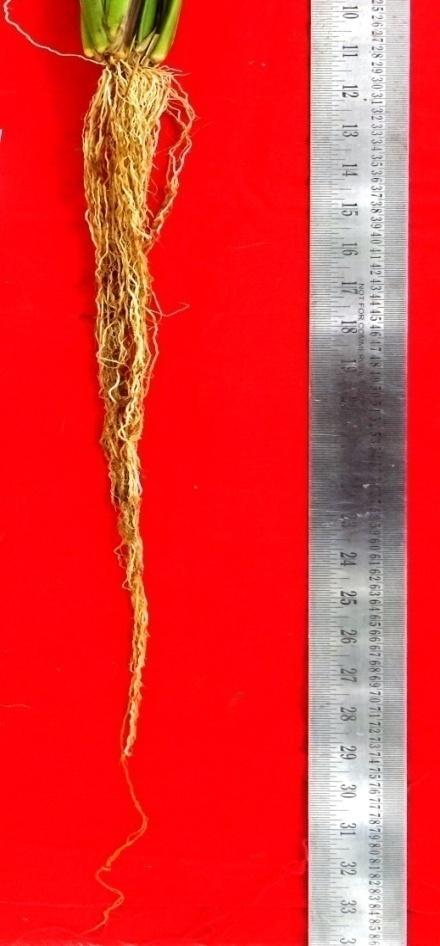 | TANU 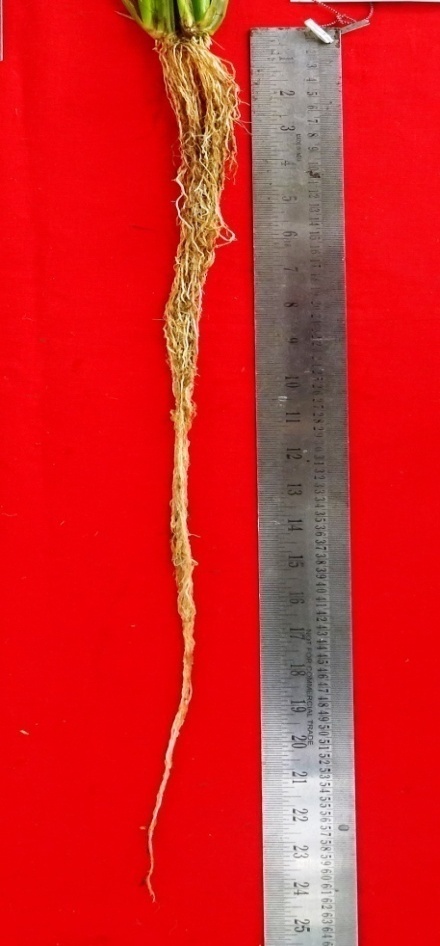 |
| RASI  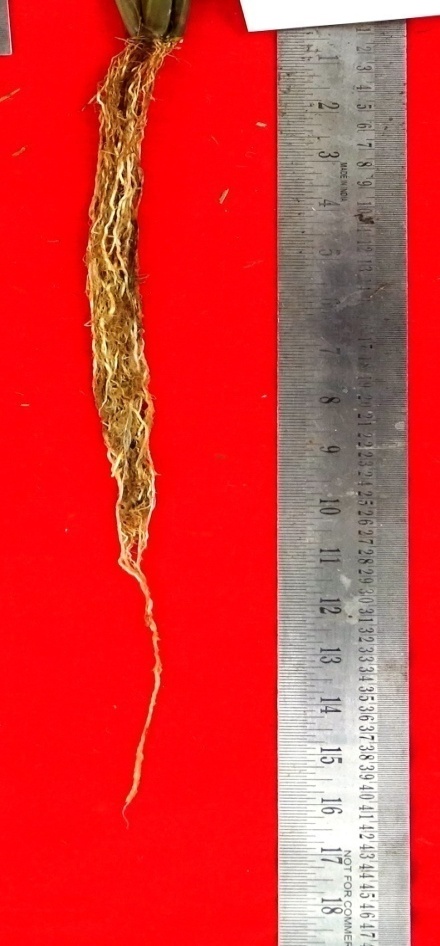 | SWARNA SUB-1 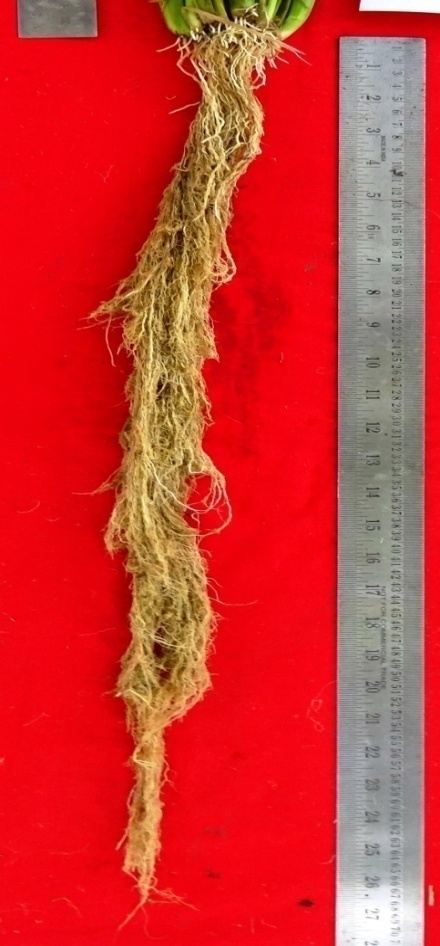 | MTU-1010 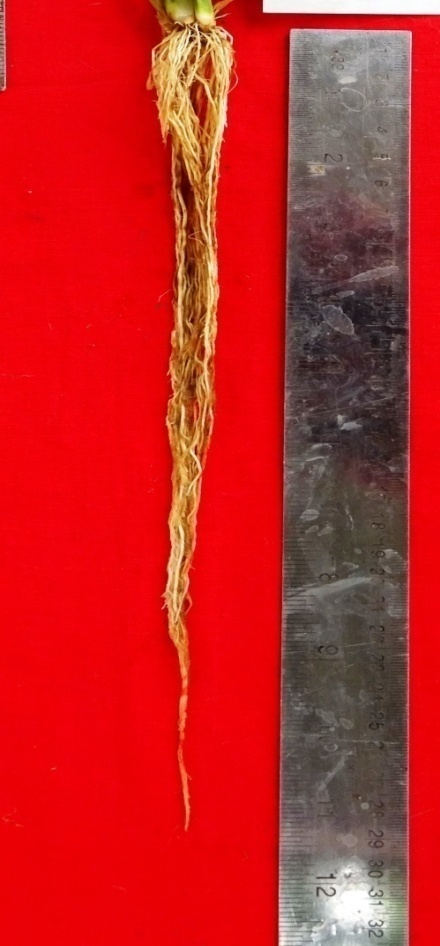 |
| BPT-5204  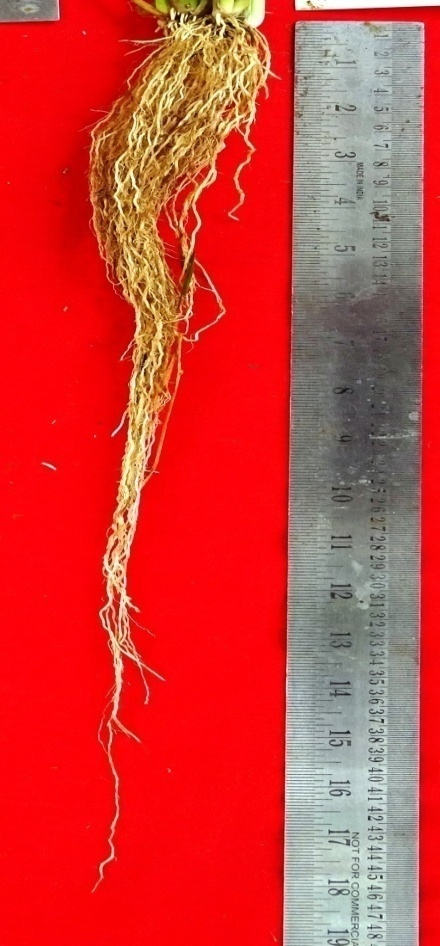 | JAYA 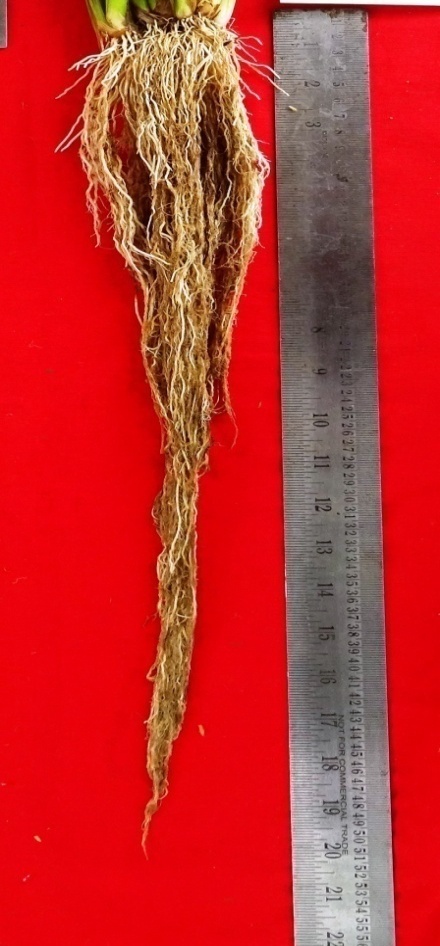 | MTU-1001 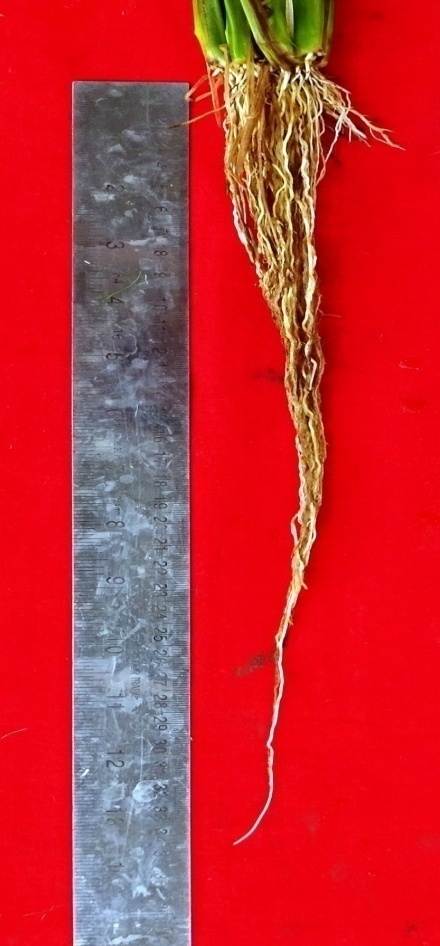 |
| TI-3  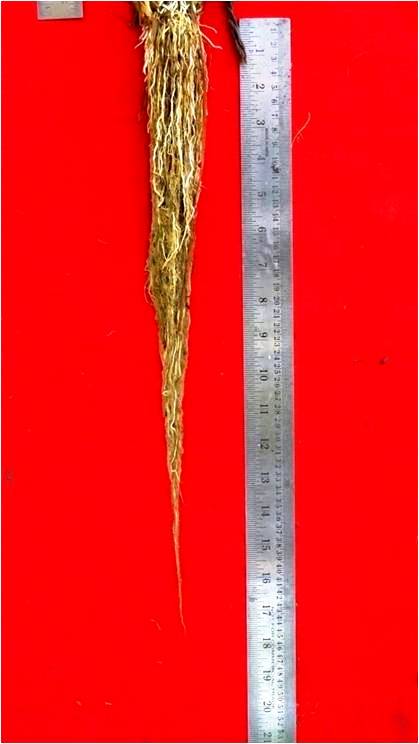 | TI-4 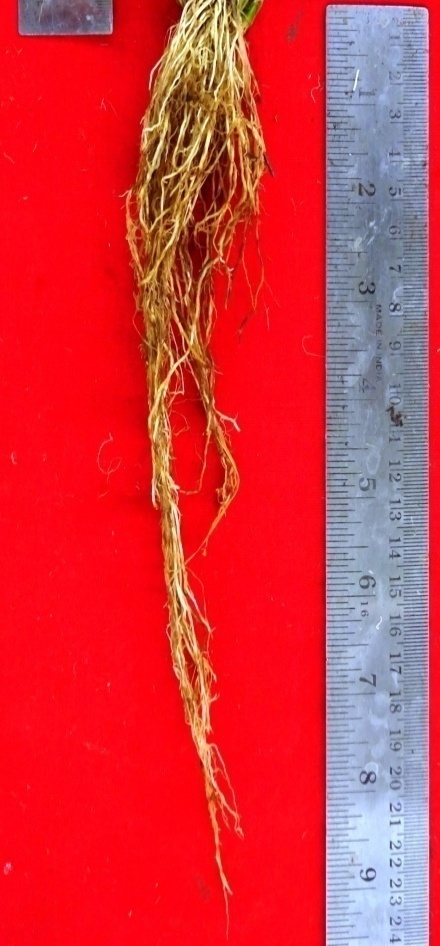 | TI-8 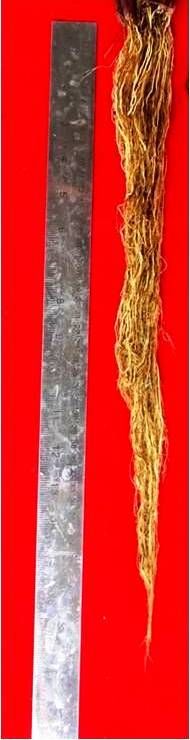 |
| TI-11  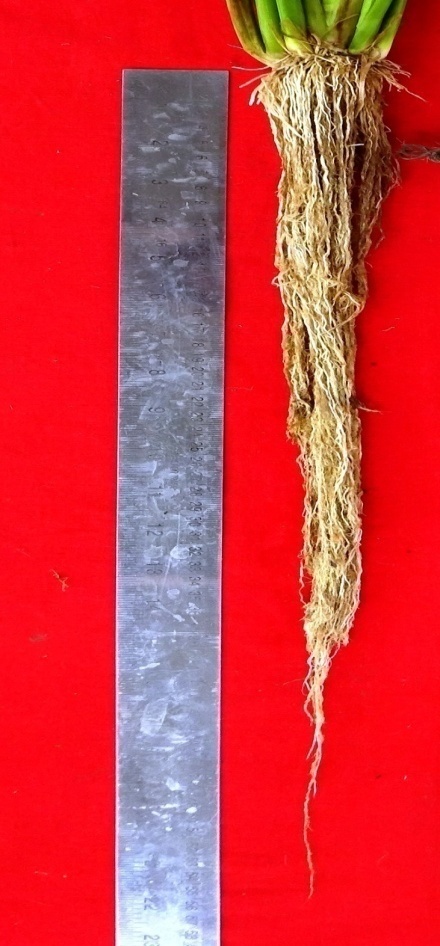 | TI-12  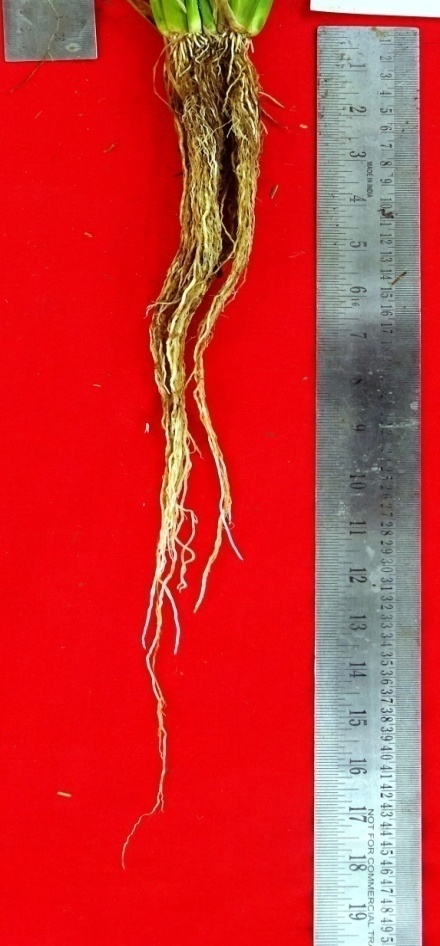 | TI-15  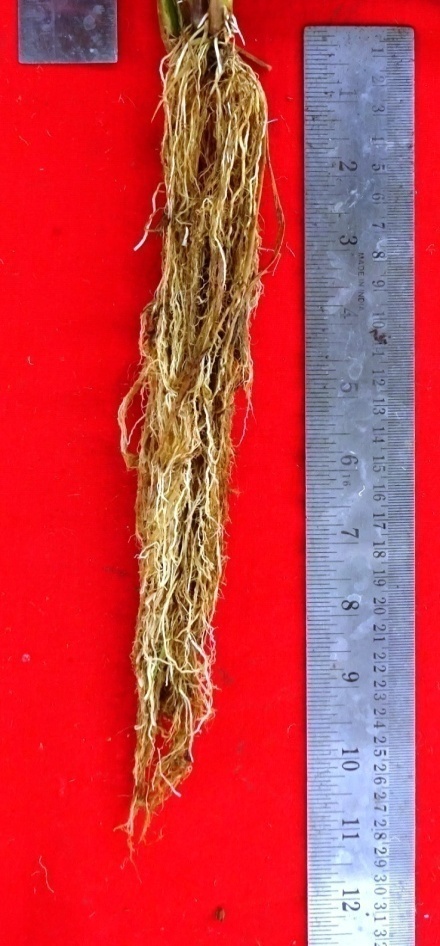 |
| TI-16  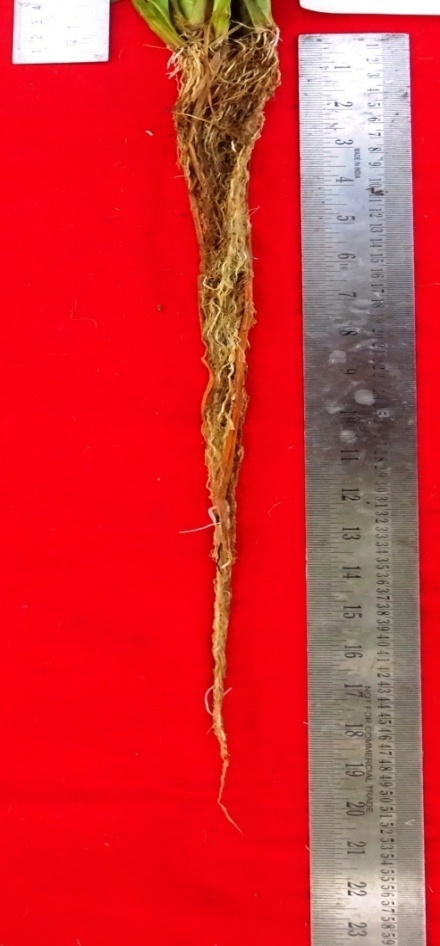 | TI-17 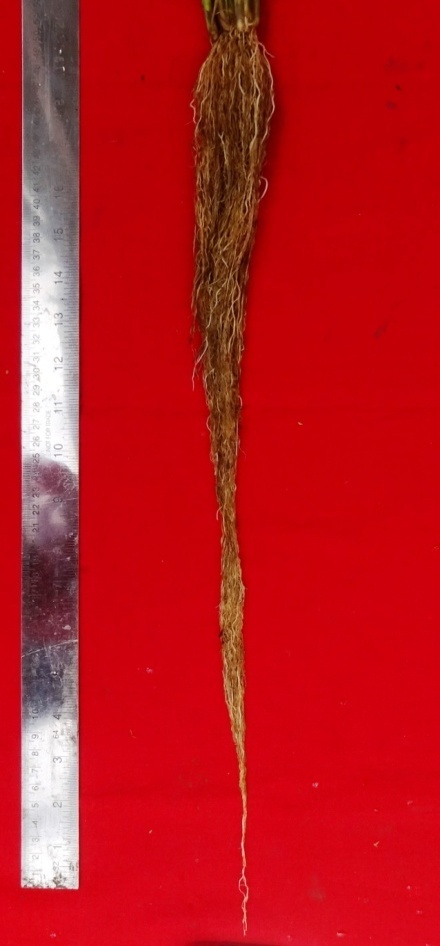 | TI-18 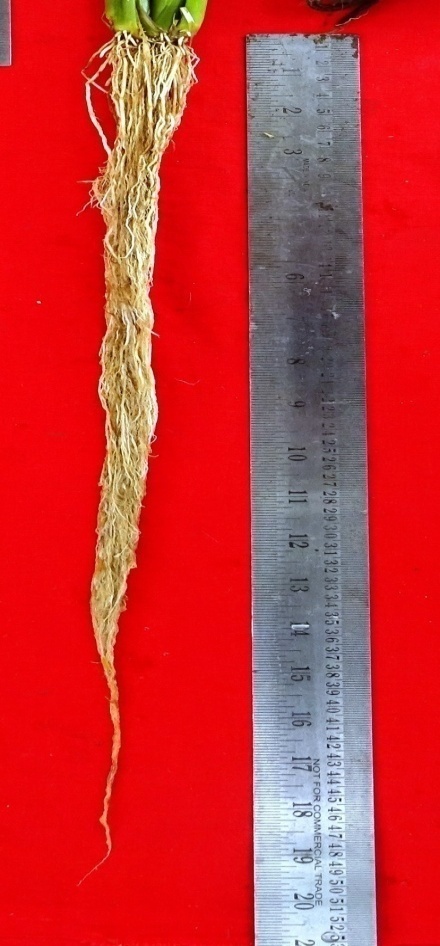 |
| TI-19  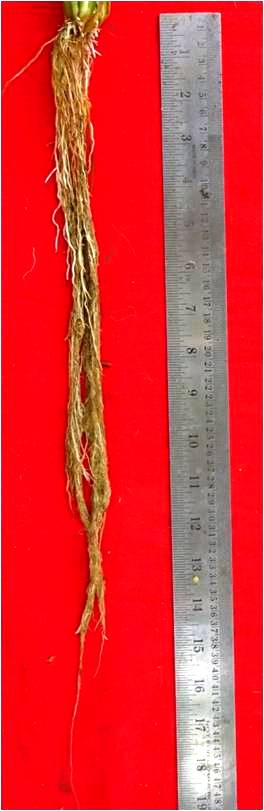 | TI-23 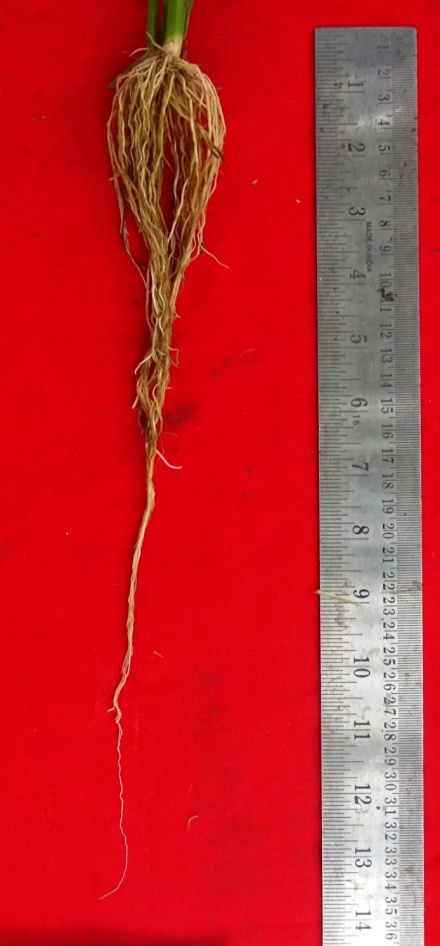 | TI-24  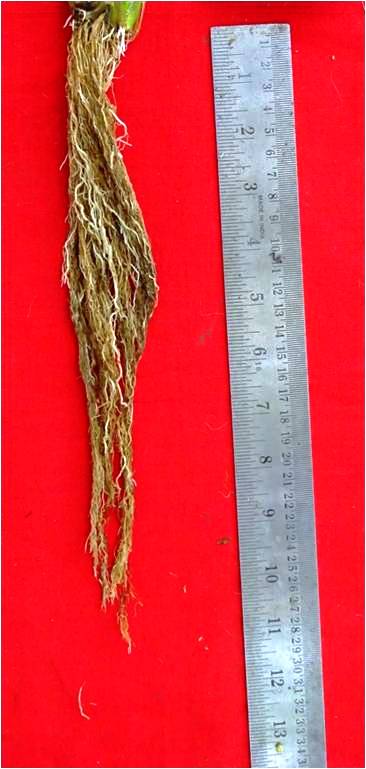 |
| TI-25  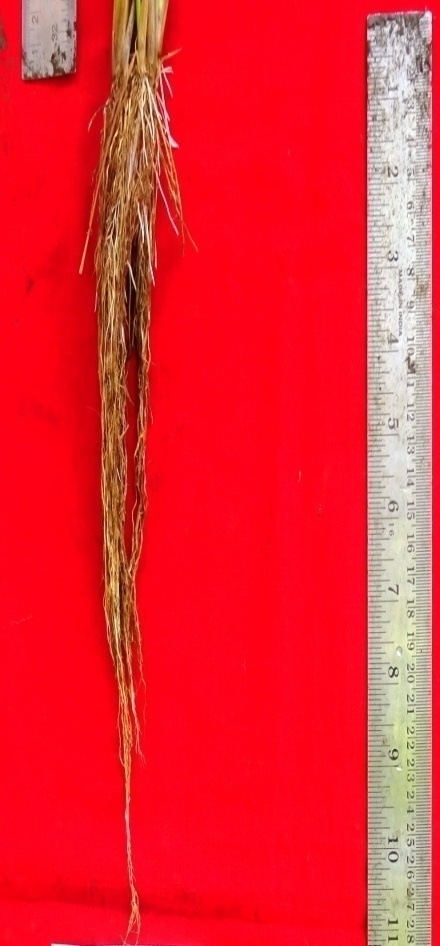 | TI-35 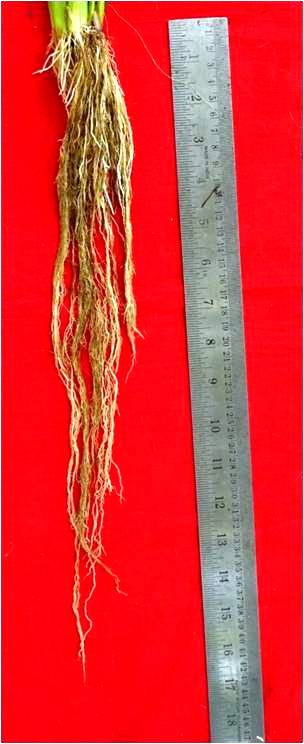 | TI-36  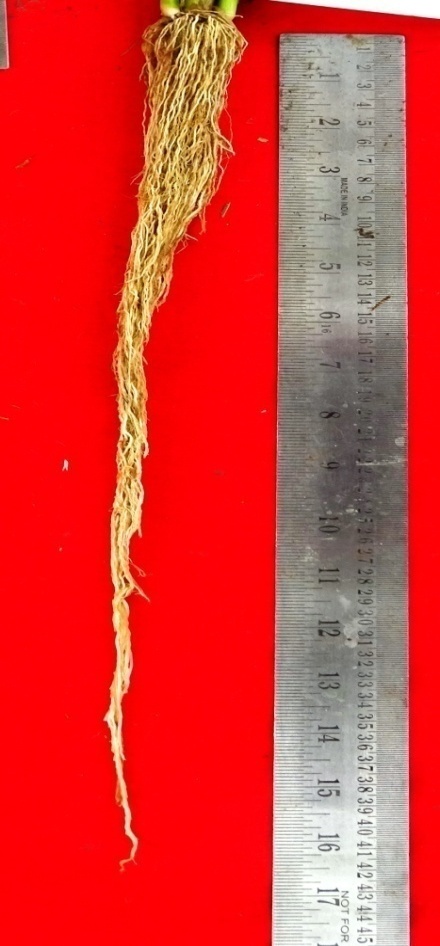 |
| TI-37  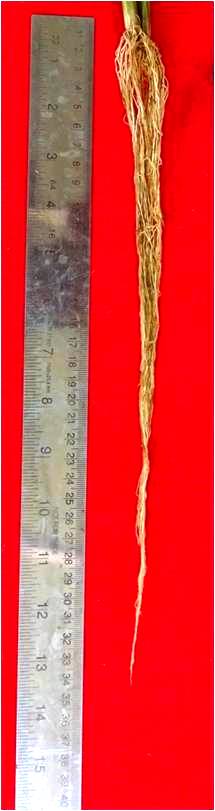 | TI-44 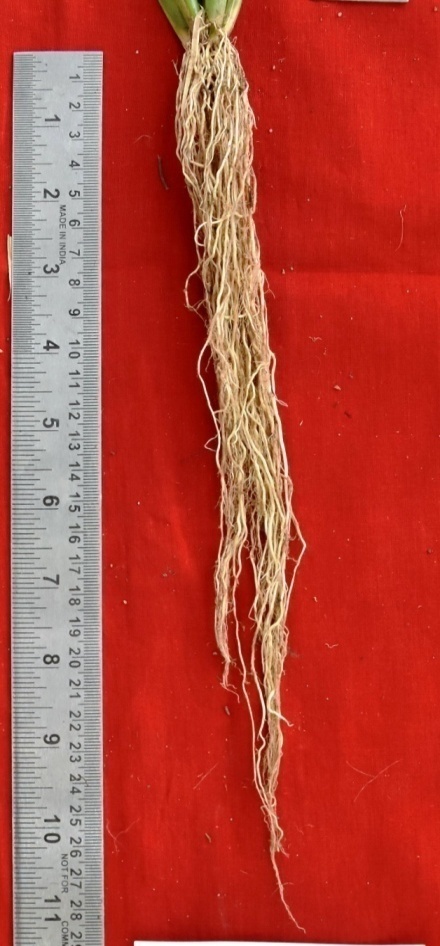 | TI-87  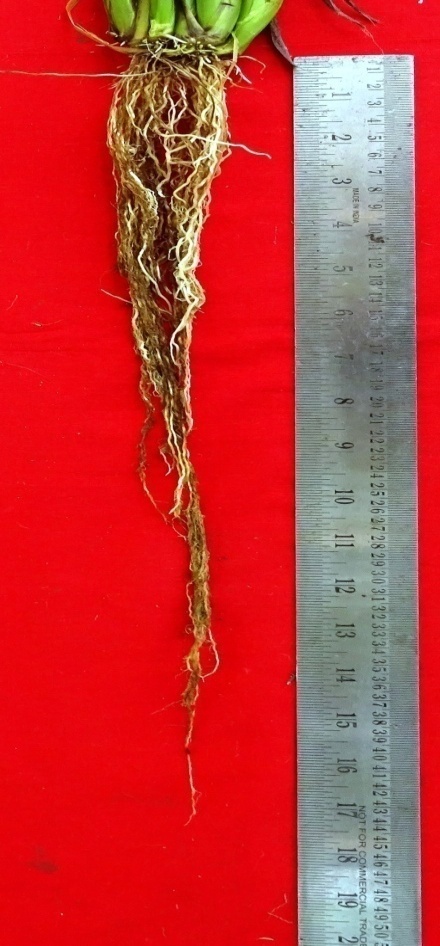 |
| TI-112  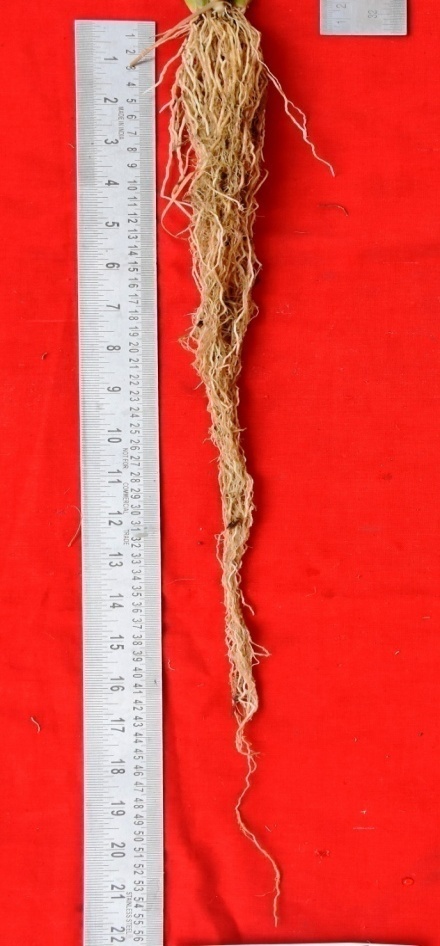 | TI-128 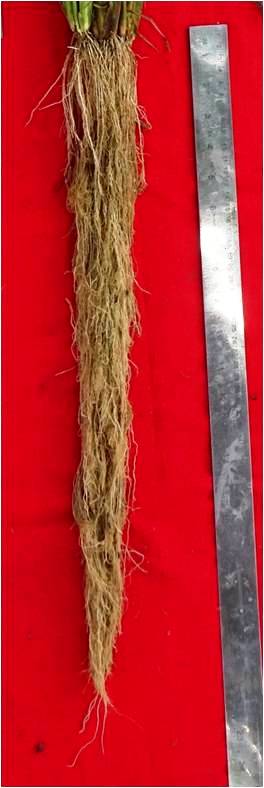 | TI-116  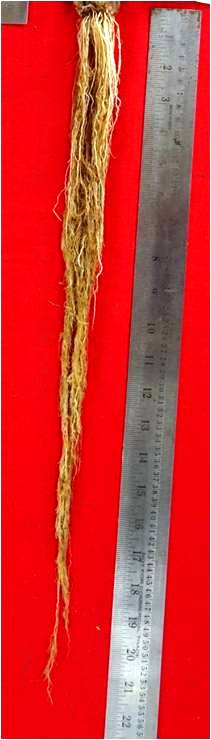 |
| TI-124  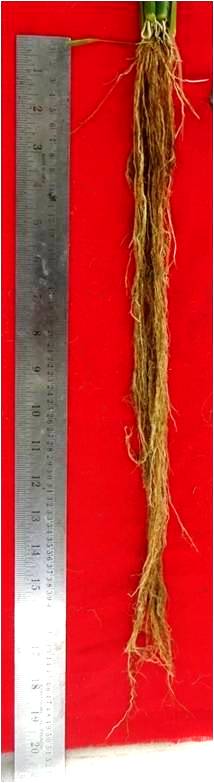 | SWARNA 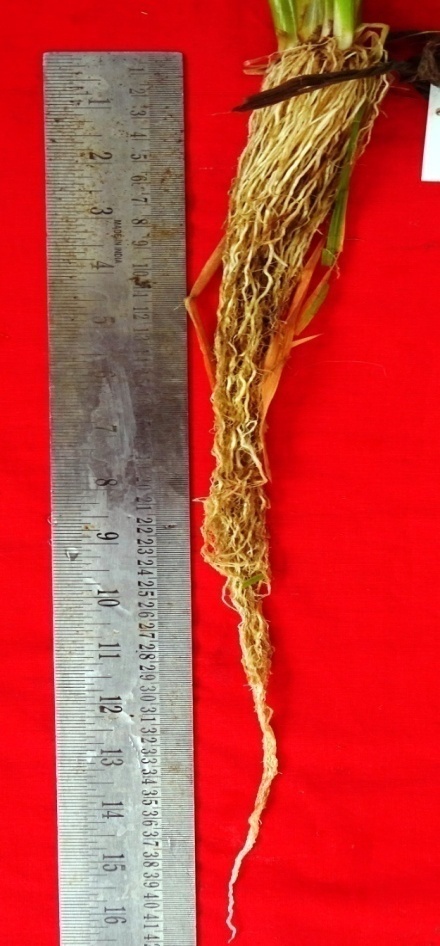 | VANDANA 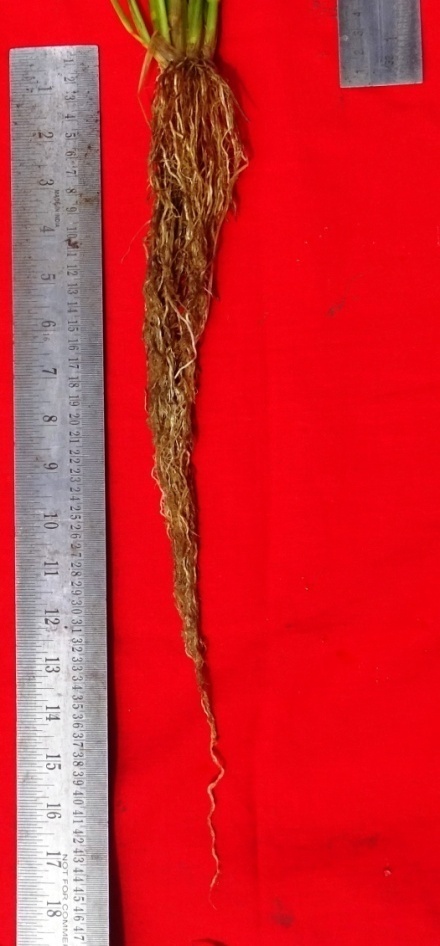 |
| WAZUHOPHEK  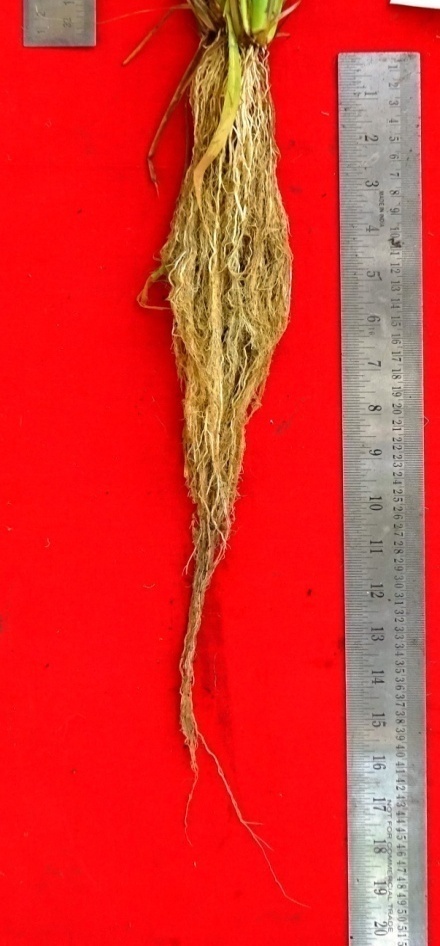 | ISM 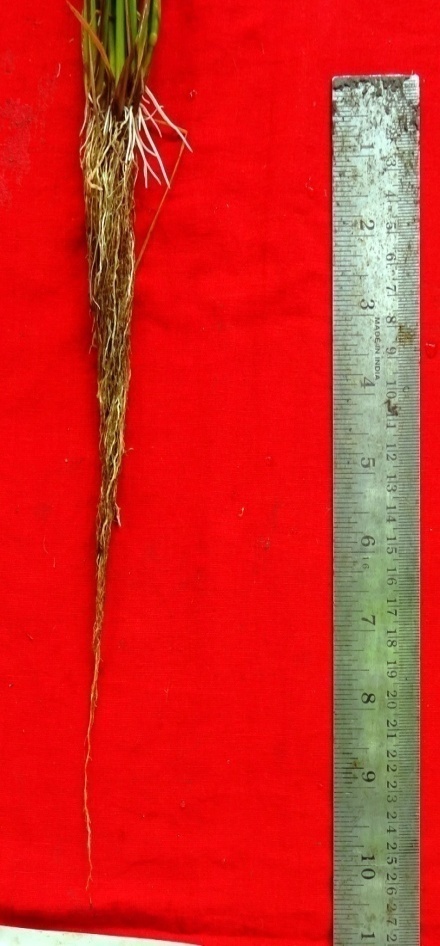 | PUP-225 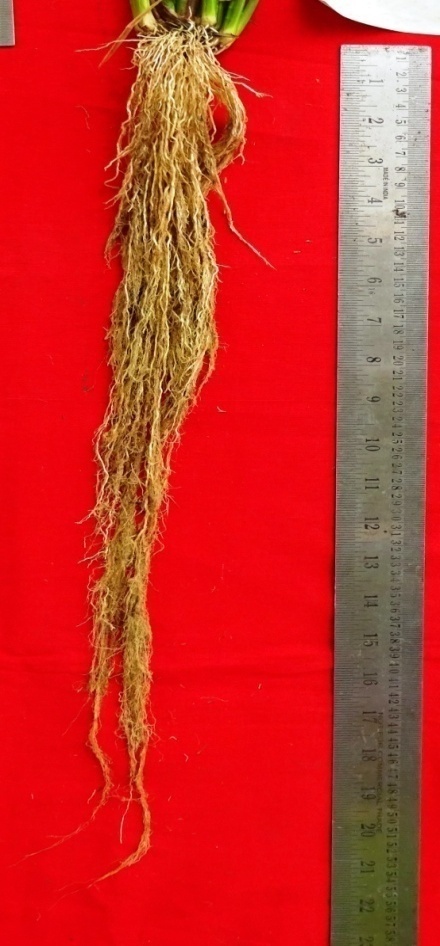 |
| PUP-229 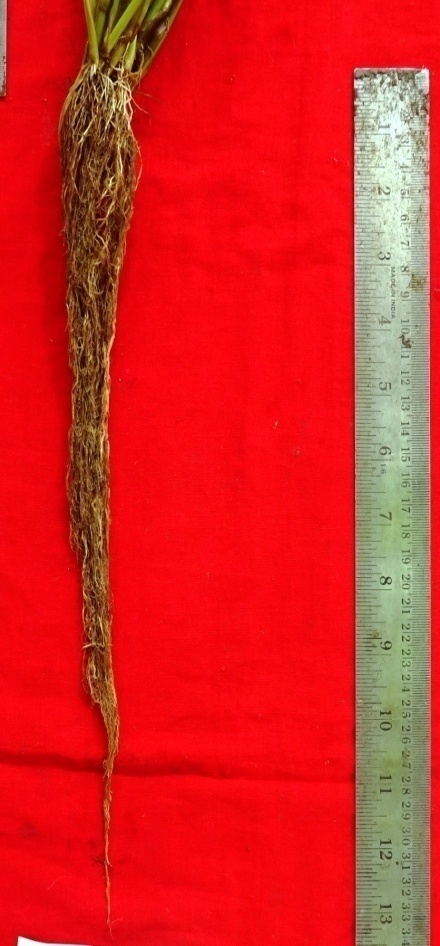 | PUP-230 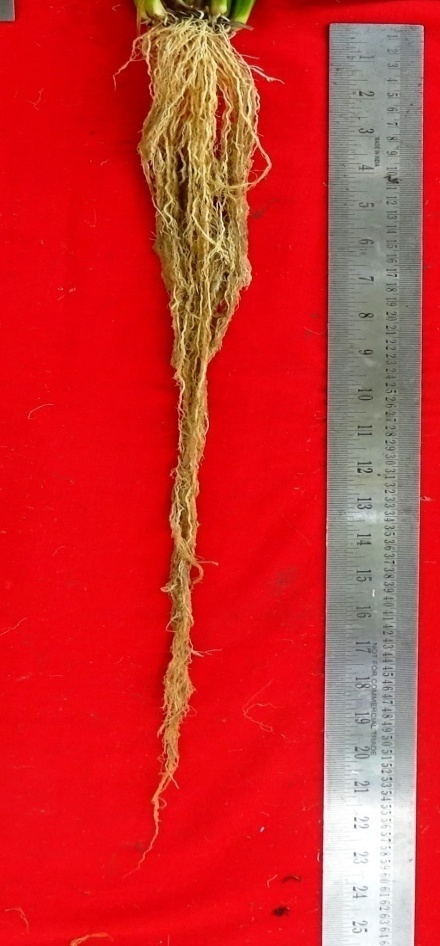 | KR-209 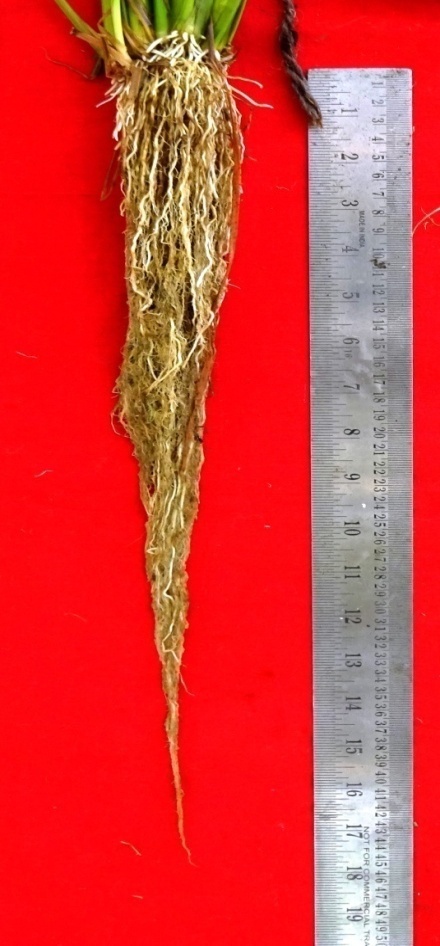 |
| KR-262 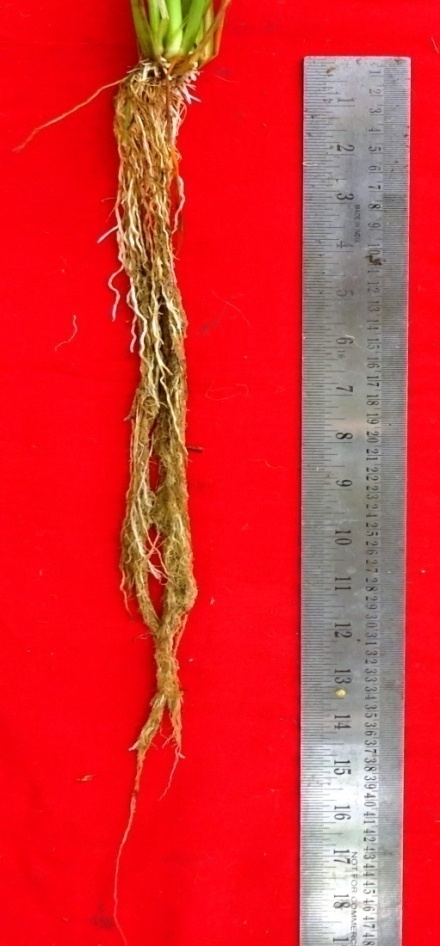 | CR DHAN-202 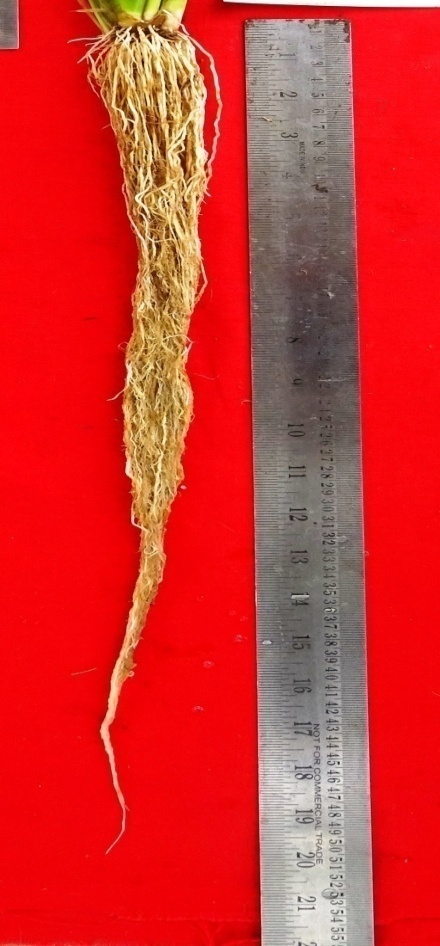 | SR-50 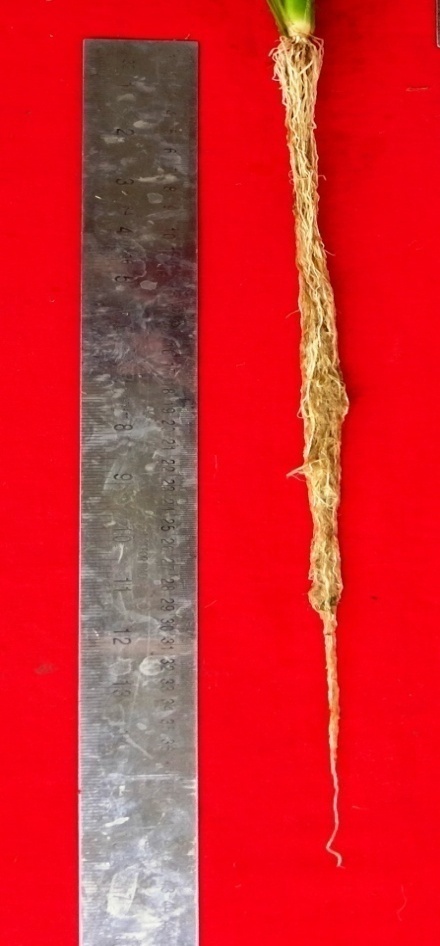 |
| MAS-946-1 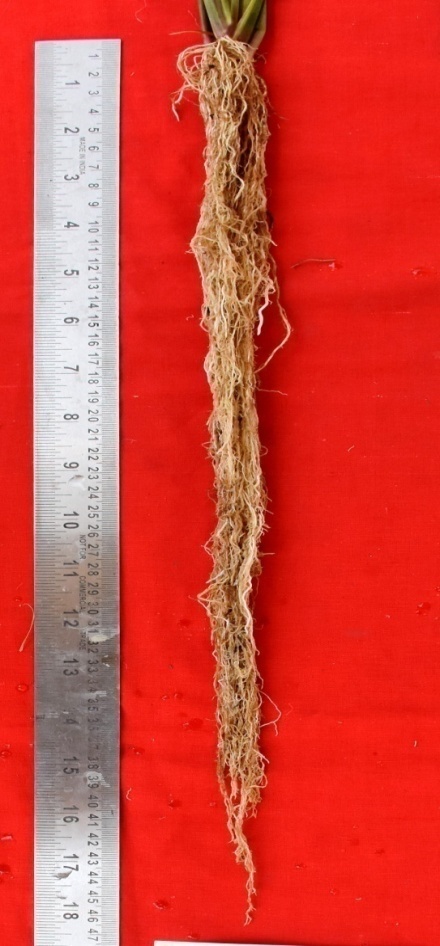 | PB-3 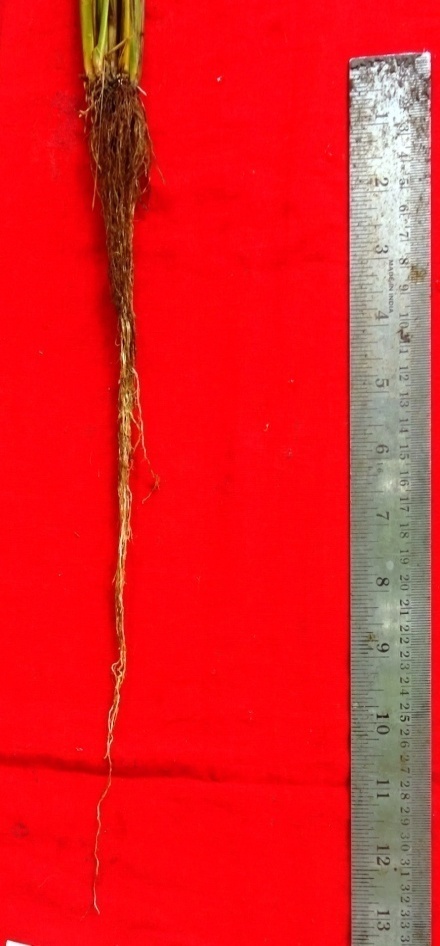 | CR DHAN-201 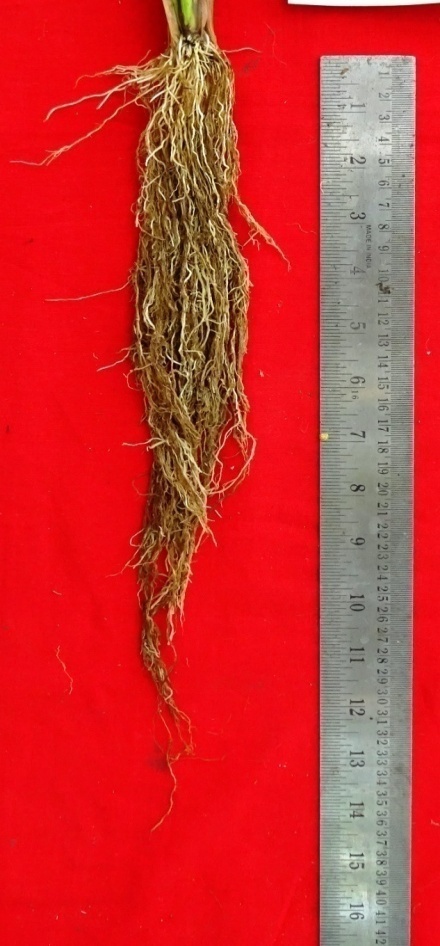 |
| DRR DHAN-42 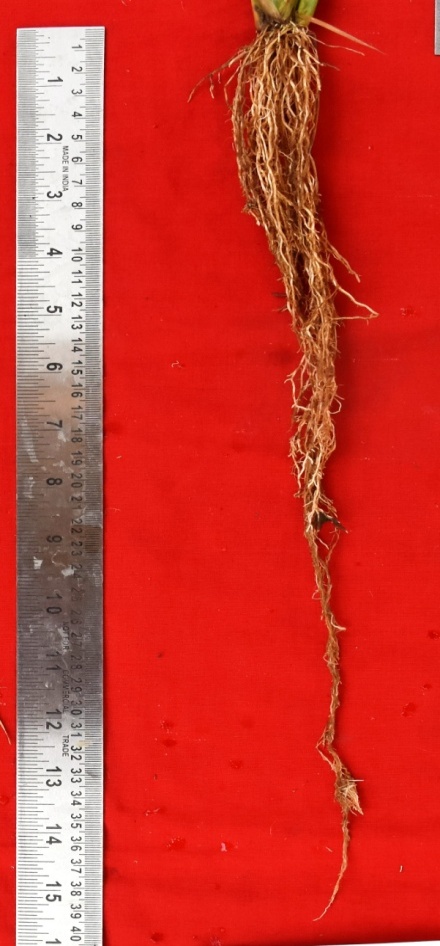 | DRR DHAN-44 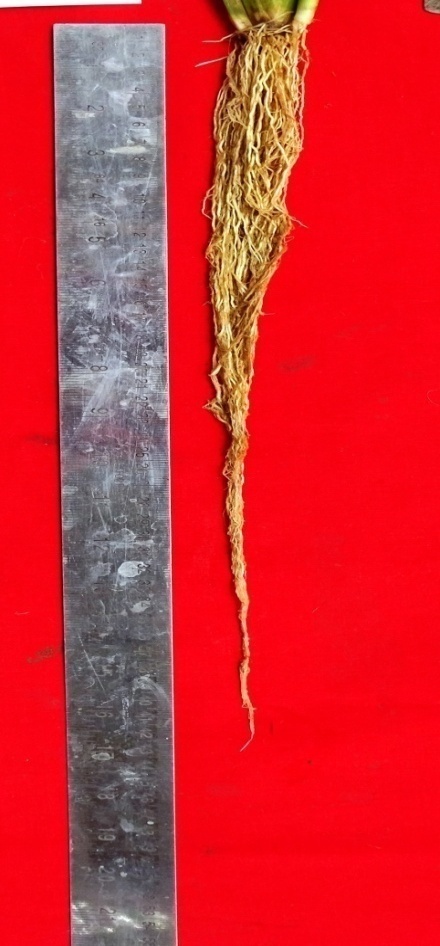 | NPS-24  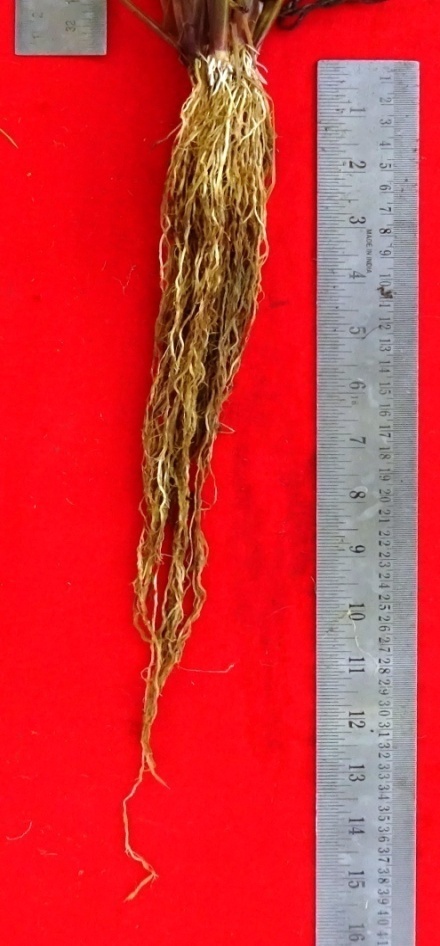 |
| NPS-53 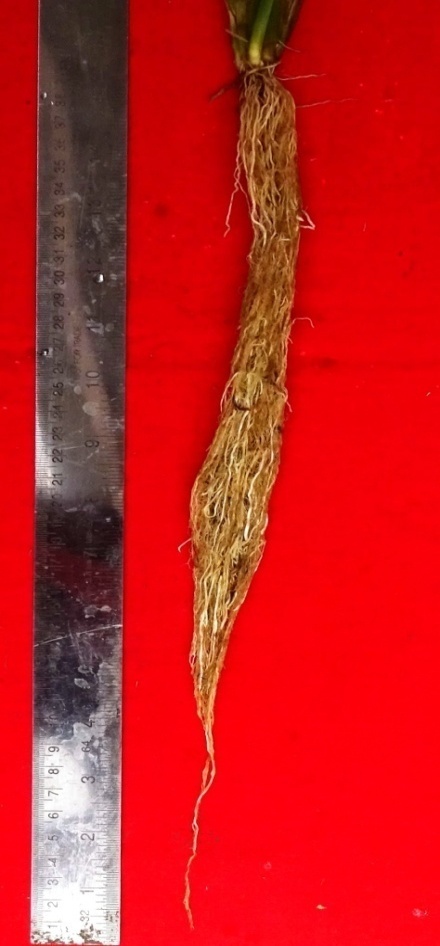 | NPS-25 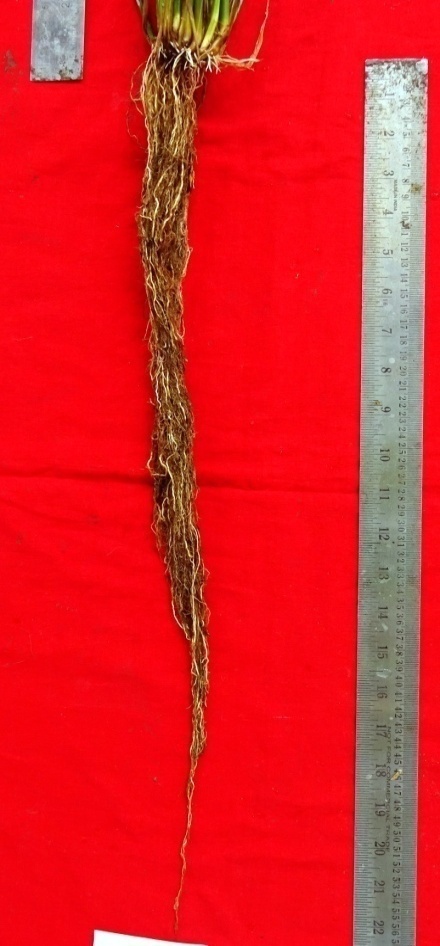 | DB-5 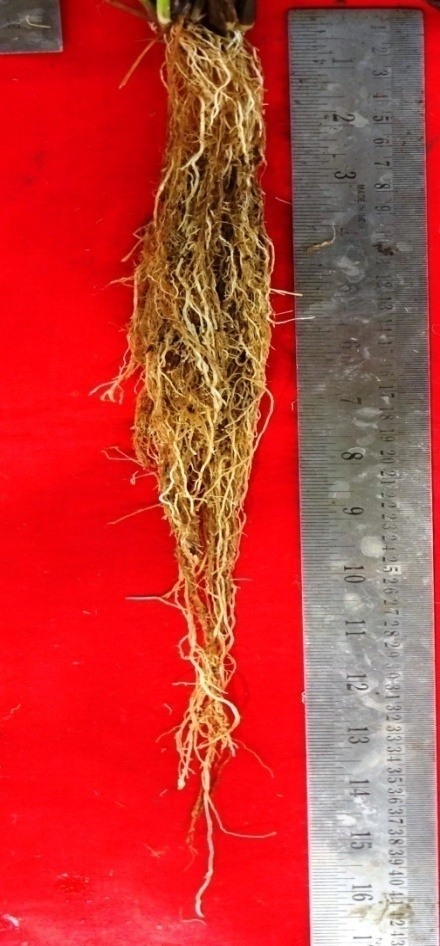 |
| DB-6 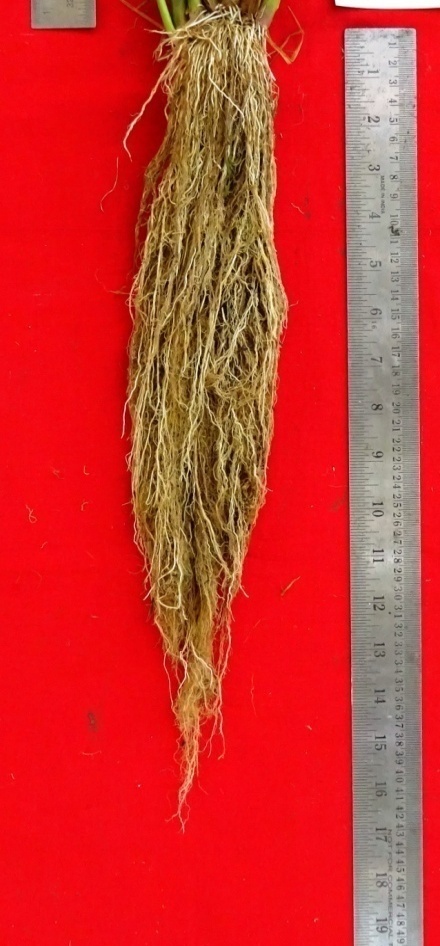 | DB-7 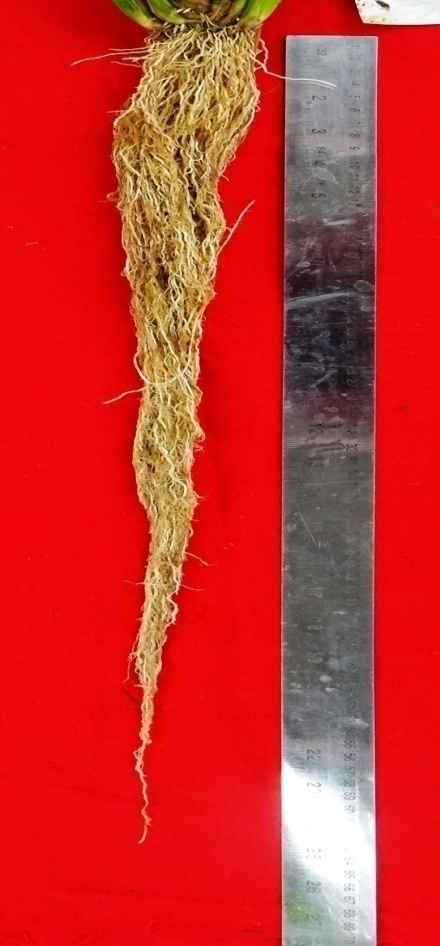 | DB-9 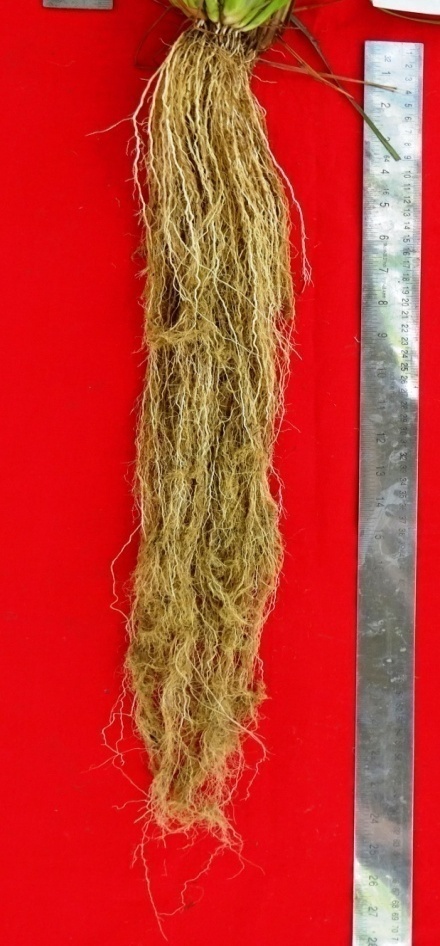 |
| NPK-13 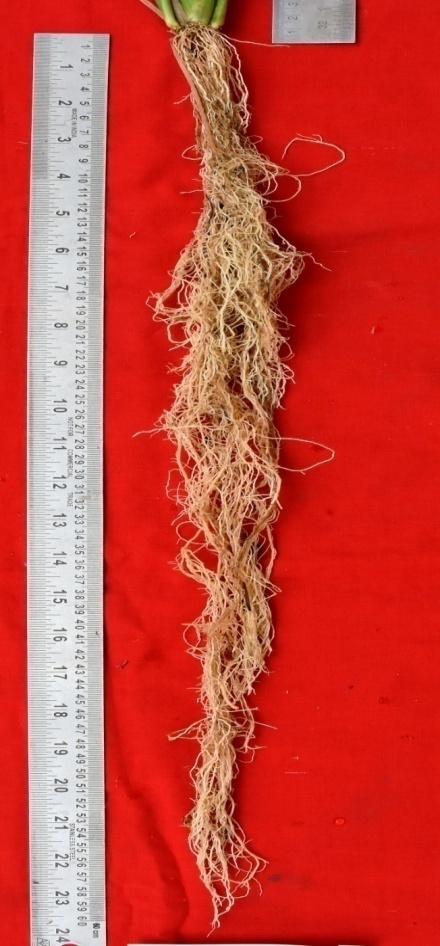 | NPK-27 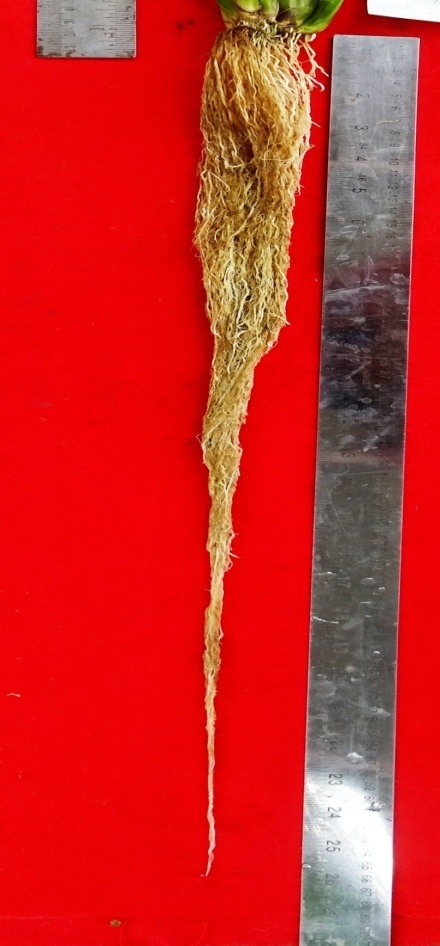 | NPK-40 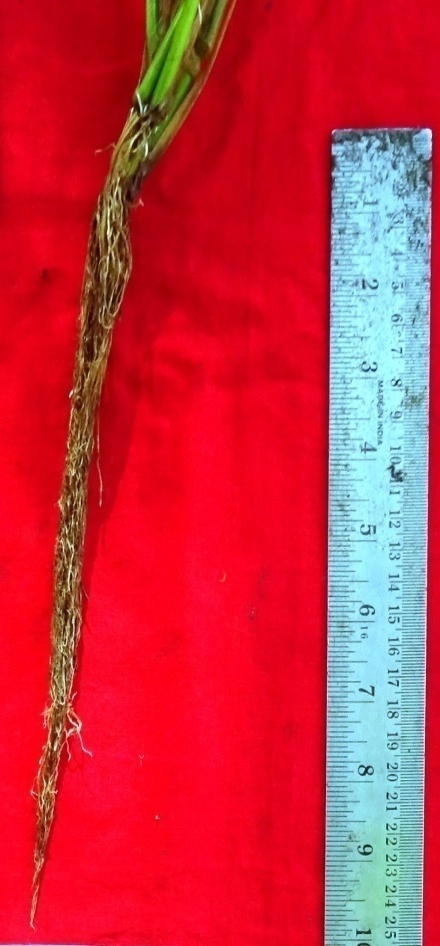 |
| NPK-41 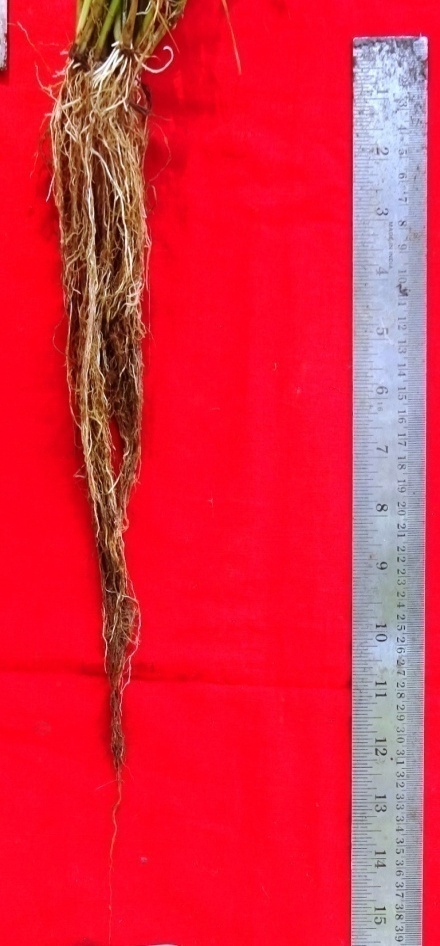 | NPK-43 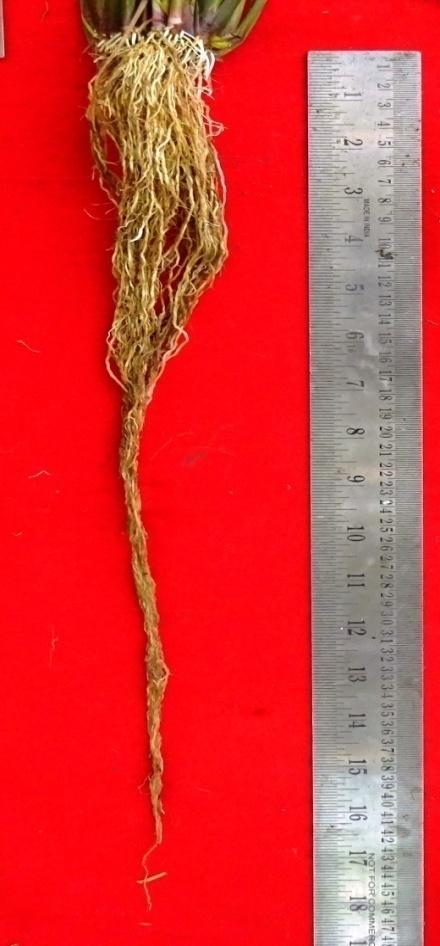 | NPK-45  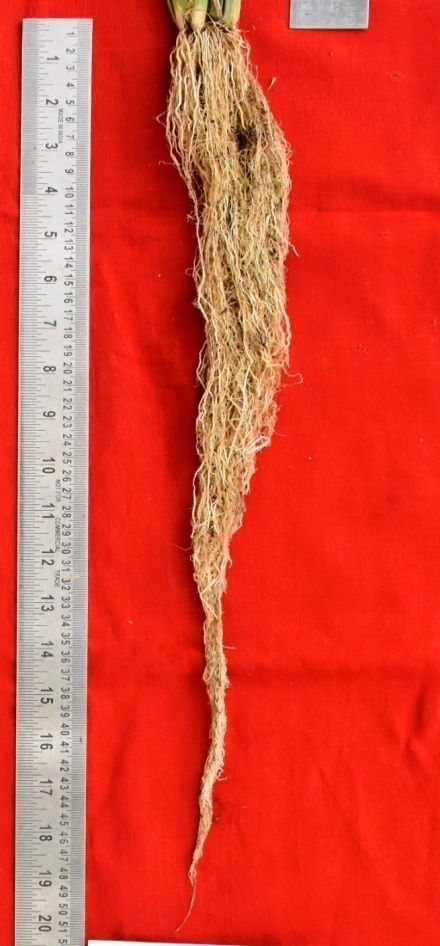 |
| SM-363  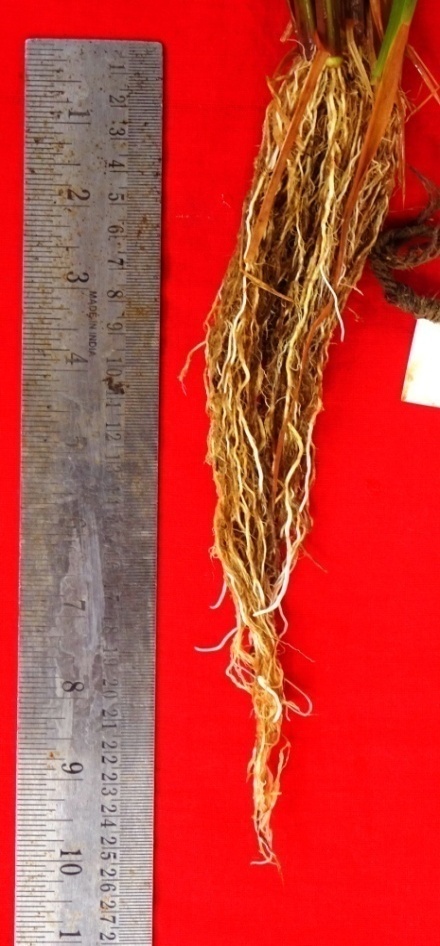 | SM-669 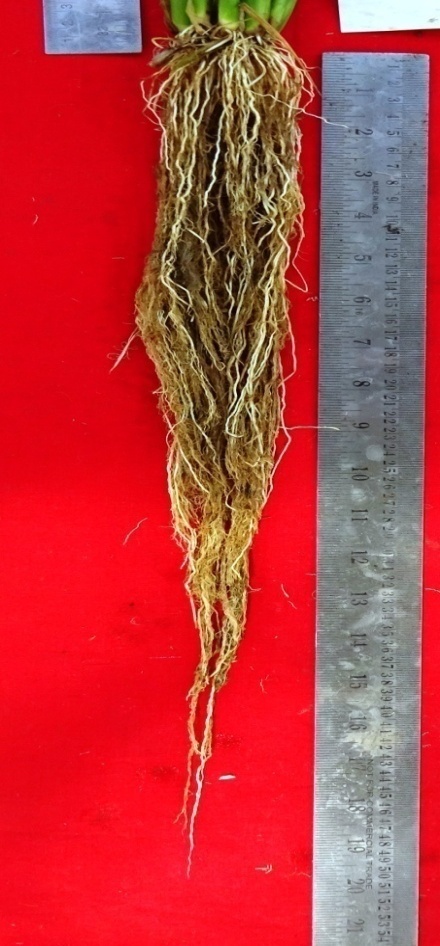 | SM-686  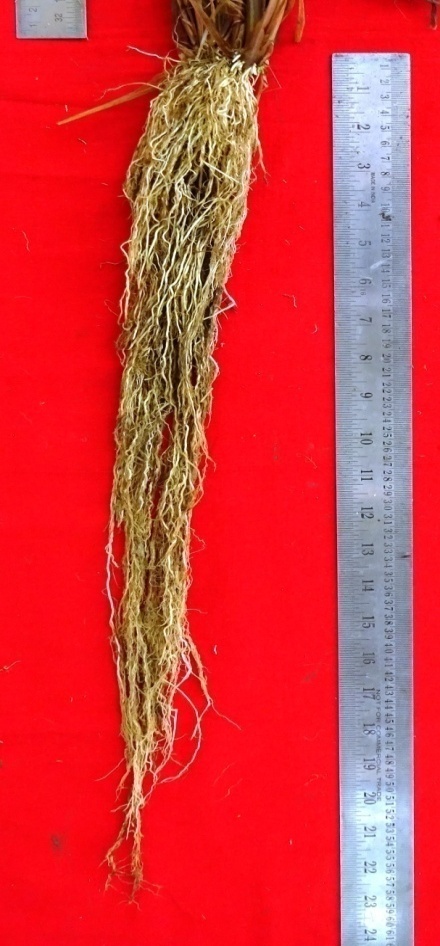 |
| PB-4 | PB-5 | SAHBHAGI DHAN |
| SABITA | DRR DHAN-41 | KMR-3 |
| IR-64 | ATR-486 | ASG-73 |
| ASG-126 | CG-219 | CG-228 |
| CG-242 | CG-243 | BASMATHI-370 |
| THURUR BHOG | D-92 | JBB-661 |
| JBB-610 | JBB-684 | JBB-1325 |
| JBB-631-1 | | |

**Supplementary Figure. S3. Root photographs of the rice association panel at the panicle initiation stage.**

| WB-30 | DB-9 |
| --- | --- |
| NPK-27 | JBB-684 |
| CG-243 | KJ-226 |

**Supplementary Figure. S4. Representative photographs of roots scanned and analyzed in WinRHIZO for root-related traits like volume, root thickness, diameter, etc. at the panicle initiation stage under the aerobic condition in the polyhouse.**

**Supplementary Figure. S5. A) Nursery view of rice association panel under irrigated condition, ICAR-IIRR.**

**Supplementary Figure S5. B) Field view of rice association panel under irrigated condition, ICAR-IIRR.**

**Supplementary Figure. S6. A) Field view of rice association panel under aerobic condition at ARS, Dhadesugur (early growth stage).**

**Supplementary Figure. S6. B) Field view of rice association panel under aerobic condition at ARS Dhadesugur (tillering stage).**

**Supplementary Figure S7. Bi-plot representing principle component analysis (PCA) of the rice association panel.**

**A**

**B**

**Supplementary Fig. S8. Distribution of F_ST_ and alpha values obtained from the structure (K=3). (A) Histogram of distribution of Fst1 (B) Histogram of distribution of Fst2**

**Supplementary Fig. S8. (C) Histogram of distribution of Fst3 (D) Histogram of distribution of Alpha X-axis represents fixation indices and alpha values and Y-axis with bootstrap (alpha >1 indicates admixtures alpha<1 indicates single population).**

| **A** | **B** |
| --- | --- |
| **C** | **D** |
| **E** | **F** |
| **G** | **H** |
| **I** | **J** |
| **K** | **L** |
| **M** | **N** |

**Supplementary Figure S9. Manhattan plots for root traits at the panicle initiation stage. The negative log10-transformed *p-values* of each trait are plotted against the marker position in the rice genome.**

X-axis represents the observed versus expected p-values for each MTAs. (A): Shoot length (cm); (B): Root length (cm); (C): Total plant length (cm); (D): SPAD; (E): Tiller number; (F): Shoot fresh weight (mg); (G): Shoot dry weight (mg); (H): Root fresh weight (mg); (I): Root dry weight (mg); (J): Total fresh weight (mg); (K): Total dry weight (mg); (L): Root average diameter (mm); (M): Root length per volume (cm/m^3^); (N): Root volume (cm^3^).
